# Wallace’s line structures seagrass microbiota and is a potential barrier to the dispersal of marine bacteria

**DOI:** 10.1186/s40793-024-00568-3

**Published:** 2024-04-18

**Authors:** Benjamin J. Wainwright, Josh Leon, Ernie Vilela, K. J. E. Hickman, Jensen Caldwell, Behlee Aimone, Porter Bischoff, Marissa Ohran, Magnolia W. Morelli, Irma S. Arlyza, Onny N. Marwayana, Geoffrey Zahn

**Affiliations:** 1 Yale-NUS College, National University of Singapore, 16 College Avenue West, Singapore, 138527 Singapore; 2 Department of Biological Sciences, National University of Singapore, Singapore, Singapore; 3 Biology Department, Utah Valley University, 800 W University Parkway, Orem, UT 84058 USA; 4 Department of Biology, Woods Hole Oceanographic Institution, Woods Hole, MA USA; 5 Research Center for Oceanography, National Research and Innovation Agency (BRIN), Jl. Pasir Putih I, Ancol Timur, Jakarta, 14430 Indonesia; 6 Research Center for Ecology and Ethnobiology, National Research and Innovation Agency (BRIN), Jl. Raya Jakarta-Bogor KM 46, Cibinong, Bogor, 16911 Indonesia; 7 Department of Ecology and Evolutionary Biology, University of California, Los Angeles (UCLA), 610 Charles E. Young Drive South, Los Angeles, CA 90095 USA

**Keywords:** Marine biogeography, Metabarcoding, Microbial dispersal, Microbiome, Seagrass, Wallace’s line

## Abstract

**Background:**

The processes that shape microbial biogeography are not well understood, and concepts that apply to macroorganisms, like dispersal barriers, may not affect microorganisms in the same predictable ways. To better understand how known macro-scale biogeographic processes can be applied at micro-scales**,** we examined seagrass associated microbiota on either side of Wallace’s line to determine the influence of this cryptic dispersal boundary on the community structure of microorganisms. Communities were examined from twelve locations throughout Indonesia on either side of this theoretical line.

**Results:**

We found significant differences in microbial community structure on either side of this boundary (*R*^2^ = 0.09; *P* = 0.001), and identified seven microbial genera as differentially abundant on either side of the line, six of these were more abundant in the West, with the other more strongly associated with the East. Genera found to be differentially abundant had significantly smaller minimum cell dimensions (GLM: t_923_ = 59.50, *P* < 0.001) than the overall community.

**Conclusion:**

Despite the assumed excellent dispersal ability of microbes, we were able to detect significant differences in community structure on either side of this cryptic biogeographic boundary. Samples from the two closest islands on opposite sides of the line, Bali and Komodo, were more different from each other than either was to its most distant island on the same side. We suggest that limited dispersal across this barrier coupled with habitat differences are primarily responsible for the patterns observed. The cryptic processes that drive macroorganism community divergence across this region may also play a role in the bigeographic patterns of microbiota.

**Supplementary Information:**

The online version contains supplementary material available at 10.1186/s40793-024-00568-3.

## Introduction

Wallace’s line describes a hypothetical boundary that separates Australasian and Asian fauna. First proposed by Alfred Russel Wallace [1] and later modified by Thomas Huxley [2], this idea has since been the subject of continued biogeographical study and debate [3–6]. The observation that multiple distinct ecoregions with sharp boundaries can exist within a close geographic range is of interest to biogeographers because there are presumably few, if any significant environmental barriers or climatic gradients that can easily explain the pronounced divisions in distributions over such short distances. Hypotheses relating to geologic and tectonic histories have been proposed to explain the observed patterns [7], though the intrinsic dispersal ability of a species has been suggested to be more important for modern patterns [8]. However, these trends do not hold consistently for flora and fauna across Wallace’s line [9]. The disparity between flora and fauna could be accounted for if dispersal limitations were the primary driving mechanism for the differences on either side of the line, with plants typically being less limited by dispersal than animals across even narrow stretches of water [10].

The idea that dispersal limitation is a key factor in the endemism of a species has led to the presumption that the geographic range of species with relatively prolific and/or long-distance dispersal adaptations should be more heavily influenced by environmental filtering than dispersal barriers [11]. It is presumed that microbes encounter minimal dispersal restrictions or barriers, attributable to their diminutive size and the dormant nature of their dispersal formations [12]. Spores, for example, have the potential for long-distance aerial and aquatic dispersal [13, 14]. Previous work on microbes in Indonesia has shown contrasting patterns. Polypore fungi showed no discernible biogeographic patterns across Wallace’s Line, providing support for the hypothesis that wind-dispersed spores may be less limited by biogeographic barriers in this region [15]. Different fungal species do have varying dispersal potentials [16] and dispersal limitation may be the norm for many fungal taxa [17]. For instance, seagrass associated fungi have demonstrably different community structure across Wallace's Line and those differences cannot be sufficiently explained by environment or plant genotype [18].

Microbes face a variety of limitations that can restrict their dispersal [19]. These limitations are determined by physical, chemical, and biological characteristics, which can vary widely between bacteria and fungi. The main mechanisms determining biogeographic patterns (e.g., selection, drift, dispersal, and mutation) play roles of varying importance in the distribution of these microorganisms in marine environments [20, 21]. Temperature-driven selection is a significant limitation for prokaryotes [22], whereas fungi produce spores capable of dispersing long distances, which should be limited by few physical barriers.

Understanding microbial dispersal and microbiota community composition provides important insights into ecosystem and human health, and determining how a species will adapt to a changing climate [23]. Without knowing what microbes are currently present in an ecosystem or habitat, it becomes impossible to assess the magnitude of any change and how increased or reduced dispersal will impact these ecosystems. Knowledge of these changes is essential in efforts to predict and manage change in marine ecosystems across the globe [23]. At the same time, climate change is simultaneously expanding and constricting the range of numerous species, including some pathogens [24]. The potential increased spread of disease is particularly relevant for seagrasses. Seagrass wasting disease and other seagrass pathogens are predicted to become more prevalent as climate change advances and dispersal patterns are altered [24, 25].

Seagrasses are ecosystem engineers [26], helping to stabilize particulate matter and protect coastlines from erosion [27]. They provide essential nursery habitat for many animals [28] and are able to sequester significant amounts of carbon [29]. Yet, despite these benefits, seagrass meadows are rapidly succumbing to anthropogenic stressors including climate change, poor land use practices, eutrophication, and habitat loss [27, 30]. Given the important role that microbes play in maintaining host health and the predicted changes in microbial distributions as climate change advances, understanding the seagrass microbiota and the processes that influence its structure will become increasingly important [31]. In this study, we examined whether the bacterial microbiota of the seagrass *Syringodium isoetifolium* are subject to the biogeographic trends associated with Wallace’s Line, and hypothesize that the microbial community patterns across this line are consistent with treating it as a potential dispersal barrier. To do this we examined seagrass-associated bacteria at 12 islands on either side of this line within the Indonesian archipelago. We chose to work with *S. isoetifolium* because it has a widespread distribution and can be readily located and identified on both sides of Wallace’s line [32, 33]. In performing this work we sought to understand whether the biogeographical patterns observed in megafauna and seagrass associated fungal distributions across Wallace’s line also structure bacterial communities, and in doing so we provide insights into the seagrass microbiota in an area of the world that is at the epicentre of global seagrass diversity, but one that remains largely unstudied [33].

## Materials and methods

### Study design and sampling

**Fig. 1:**
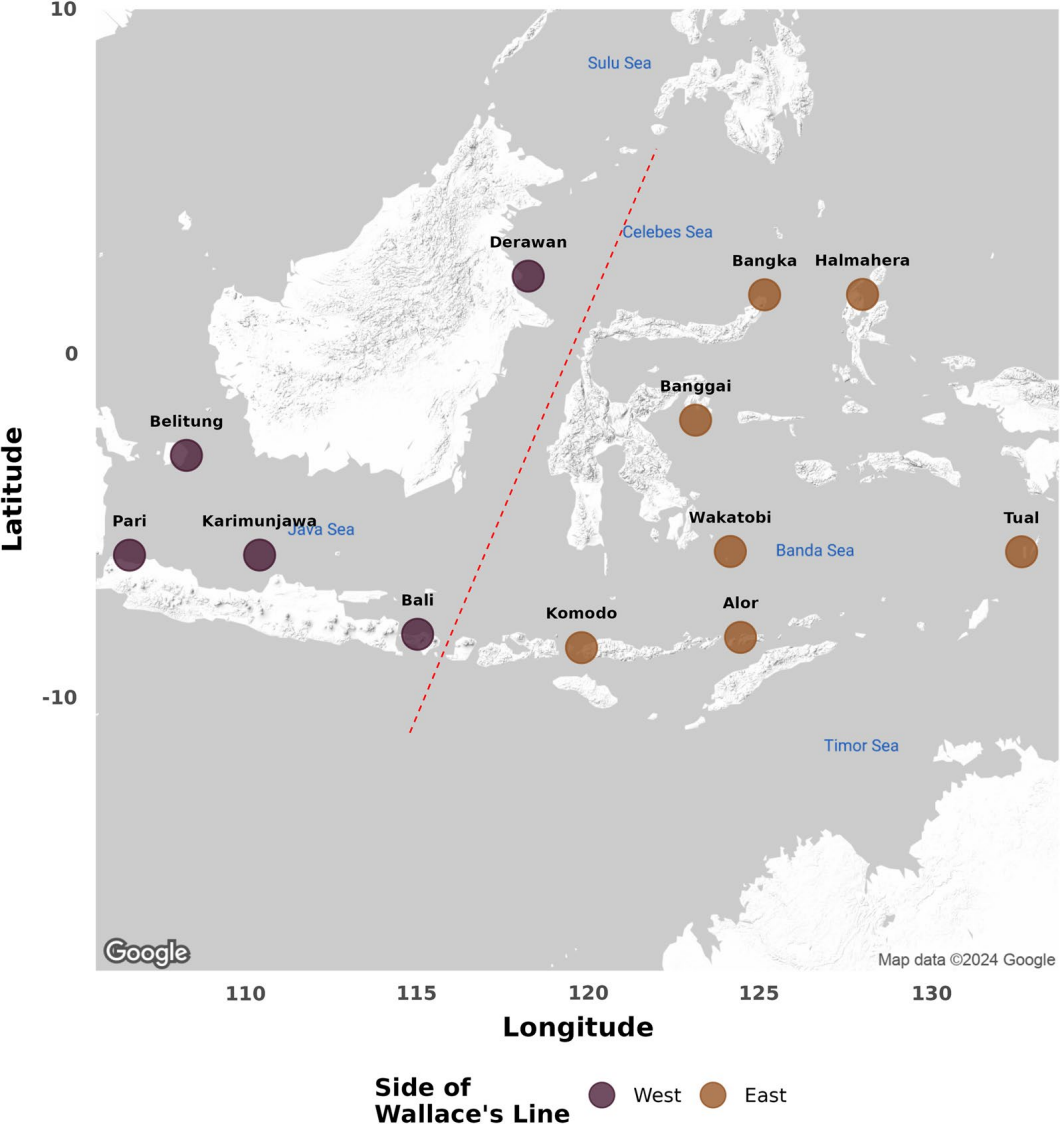
Map of study locations. Map of sampling locations for this study. Colour indicates side of Wallace’s line. Dashed line indicates approximate position of Wallace’s line. Each location was subsampled 16 times. Exact GPS coordinates are available in Additional file 1: Appendix S6

We collected the widespread seagrass, *S. isoetifolium*, from twelve sampling locations throughout the Indonesian Archipelago, and on both sides of Wallace’s line (Fig. 1). All collected seagrass blades appeared healthy, with no visible signs of disease and were free of epiphytes. To ensure all seagrass blades were similar in age, all collected samples were the same length, measuring approximately 15 cm from base to tip [34]. Full details of collection locations, sample sizes and collection dates have been reported previously [32] and are also found in the Supporting Information. To avoid collecting the same individual twice due to clonality, all seagrass blades were collected at least 20 m apart from each other. Once collected, blades were immediately placed in individual sterile tubes containing silica gel and remained unopened until DNA extraction was performed.

### Molecular methods and sequencing

DNA extraction was performed on an entire blade and all extraction procedures followed those described in the NucleoSpin Plant II CTAB protocol, Machery Nagel GmbH and Co. (Bethlehem, PA, USA). Briefly, an entire blade was homogenised using a rotor–stator homogenizer, and approximately 200 mg of this homogenate was used in DNA extraction. To determine whether the manufactures CTAB or SDS based extraction protocol was most efficient, we initially tried both techniques and compared the results on a 1% agarose gel. The CTAB protocol was the most efficient, yielding high molecular weight DNA with the highest yield (determined by band intensity). Using the 515F (GTGYCAGCMGCCGCGGTAA) [35] and 806R (GGACTACNVGGGTWTCTAAT) [36] primer set we amplified the 16S rRNA gene V4 region. Forward and reverse primers were modified to include linkers, Illumina flow cell adaptors and a unique barcode [37]. To prevent preferential amplification of chloroplast and mitochondrial DNA we included the following peptide nucleic acids (PNAs) (mPNA: GGC AAG TGT TCT TCG GA; pPNA: GGC TCA ACC CTG GAC AG) (PNAGENE, Daejeon, South Korea) [38]. Each reaction was performed in 25 µl containing 12.5 μl KAPA PCR buffer, 0.75 μl of each primer at 10 μM, 2.5 μl of mPNA and 2.5 μl pPNA at 50 μM, 1.5 μl of 1.5 mg/ml bovine serum albumin, 0.1 μl of KAPA 3G Enzyme (Kapa Biosystems, Inc, Wilmington, MA, USA), 1 μl of undiluted template DNA and water to 25 μl. Amplification success was verified using a 1% agarose gel in TAE buffer. PCR products were normalized to equal molarities and cleaned using SequalPrep normalization plates (Invitrogen, Frederick, Maryland, USA). Sequencing was performed on the Illumina MiSeq platform (600 cycles, V3 chemistry, 300-bp paired-end reads) with a 30% PhiX spike (Macrogen, Inc).

### Bioinformatics and statistical analyses

Full details of all bioinformatics steps and statistical analyses can be found in the archived GitHub repository [39]. Quality filtration and statistical analyses were performed in R v 4.2.2 [40]. We utilized ‘cutadapt’ v 4.2 [41] to remove primers from the demultiplexed fastq files. Reads were then filtered and trimmed using the ‘DADA2’ R package, v 1.24.0 [42] to infer amplicon sequence variants (ASVs). This process included removing any reads with ambiguous bases, truncating reads when the first quality score dropped below 2 (a quality score of 2 represents low quality reads of Q15 or less), and truncating all reads at 250 bases. After denoising and filtration, we removed any reads with fewer than 100 bases after trimming, and removed singletons. Potential contaminant sequences were inferred via the prevalence method from sequenced PCR negatives using the ‘decontam’ R package v 1.18.0 [43].

ASV sequences were aligned with the 'DECIPHER' R package v 2.26.0 [44] using the profile-to-profile method and a UPGMA guide tree. The alignment was used to estimate a maximum likelihood phylogenetic tree using the TN93 model within the 'phangorn' R package v 2.11.1 [45]. ASVs were assigned taxonomy against the SILVA training set v 138.1 [46] with the RDP Classifier algorithm [47] within 'DADA2.' All analyses were performed at the ASV level. Since exact matching against the 16S region, as opposed to a typical 97% threshold, is preferable [48] we did not assign species-level taxonomy except to those with exact matches in the SILVA training set. Any ASVs not unambiguously assigned to Kingdom Bacteria, as well as those assigned to mitochondrial and chloroplast lineages, were removed from analyses, as were any putative chimeras.

### Diversity measures

The ASV table, metadata, phylogenetic tree, and taxonomic assignments were imported into the ‘phyloseq’ package v 1.40.0 [49], within R for downstream analyses. Alpha diversity was estimated for relative abundance-transformed ASV counts [50], using both Shannon diversity and taxon richness, and fit with a mixed-effects model using island as a random factor nested within Wallace’s Line as predictors. Beta diversity was estimated with both Bray–Curtis dissimilarity and a weighted Unifrac distance [51]. Non-metric multidimensional scaling (NMDS) and permutational analysis of variance (PerMANOVA) were performed using the ‘vegan’ R package v 2.6.4 [52] using the same model structure as alpha diversity. Distance decay of community similarity was tested both with a Mantel Test using the 'vegan' R package and with multiple regression on distance matrices (MRM) using the 'ecodist' R package v 2.0.9 [53] with 1000 permutations.

### Differential abundance

We employed four methods to detect ASVs that showed significant differential abundance across Wallace’s line. First, we used a beta-binomial model with side of Wallace’s line as a predictor for both abundance and variance to detect differential abundance using the 'corncob' R package [54]. Second, we performed a multi-level pattern analysis using the 'indicspecies' R package [55] to look for taxa that were significantly associated to the East or West of Wallace’s line. Thirdly, we fit a random forest classification model with 999 permutations using the 'ranger' R package [56] wherein the relative abundances of all taxa were used to predict 'East' or 'West' and then selected predictive taxa using the 'vip' R package [57]. Finally, we fit the Sloan Neutral Community Model for Prokaryotes to the distributions of ASVs in our data [58, 59] and identified ASVs that differed significantly from neutral model predictions. ASVs were only determined to have significantly differential abundance on either side of Wallace’s line if they were identified by all four methods. Taxonomic classifications of differentially abundant ASVs are reported to the genus level since only 2% of our ASVs could be unambiguously assigned to a species name.

### Bacterial morphology

The BacDive database [60] was accessed with the 'BacDive' R package v 0.8.0 [61] to extract cell morphology for each taxon in the study. All taxa associated with a given genus in the study were retrieved and cell dimensions, surface area, and shape were extracted with a custom R script. Distributions of quantitative morphological features (minimum dimension and surface area) were compared between significant taxa and the overall distributions for all detected taxa using generalized linear models. Cell shape was compared similarly between significant and non-significant taxa using a Chi-squared goodness-of-fit test. All cell morphology data are included in the Additional file 1: Appendix S5.

All raw sequence data associated with this study is available in the Sequence Read Archive (SRA) under accession PRJNA944167. All sample metadata and fully documented analysis code is provided as a Zenodo release of the project GitHub repository [40].

## Results

### Sequencing and taxonomy

Sequencing yielded 10,399,720 raw reads. After quality control, read pair merging and chimera removal, 5,785,919 reads were left with a mean read count of 53,732 per sample (max = 75,235 & min = 12,524). These reads were sorted into 3022 amplicon sequence variants (ASVs) representing 309 bacterial genera. Only 65 ASVs (2%) were unambiguously assigned to bacterial species by exact 16S matching. Therefore, in this work we report taxonomic information at the genus level.

### Diversity measures

**Fig. 2:**
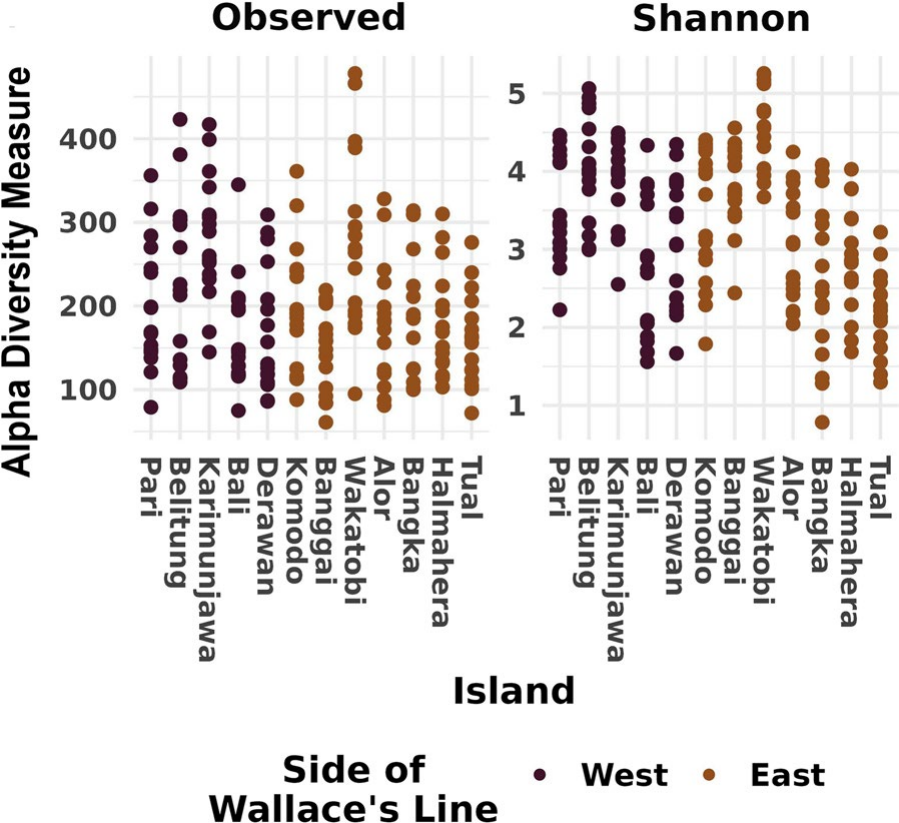
Alpha diversity measures. Observed ASV richness (left panel) and Shannon diversity (right panel) estimates. Locations arranged from West to East

**Fig. 3:**
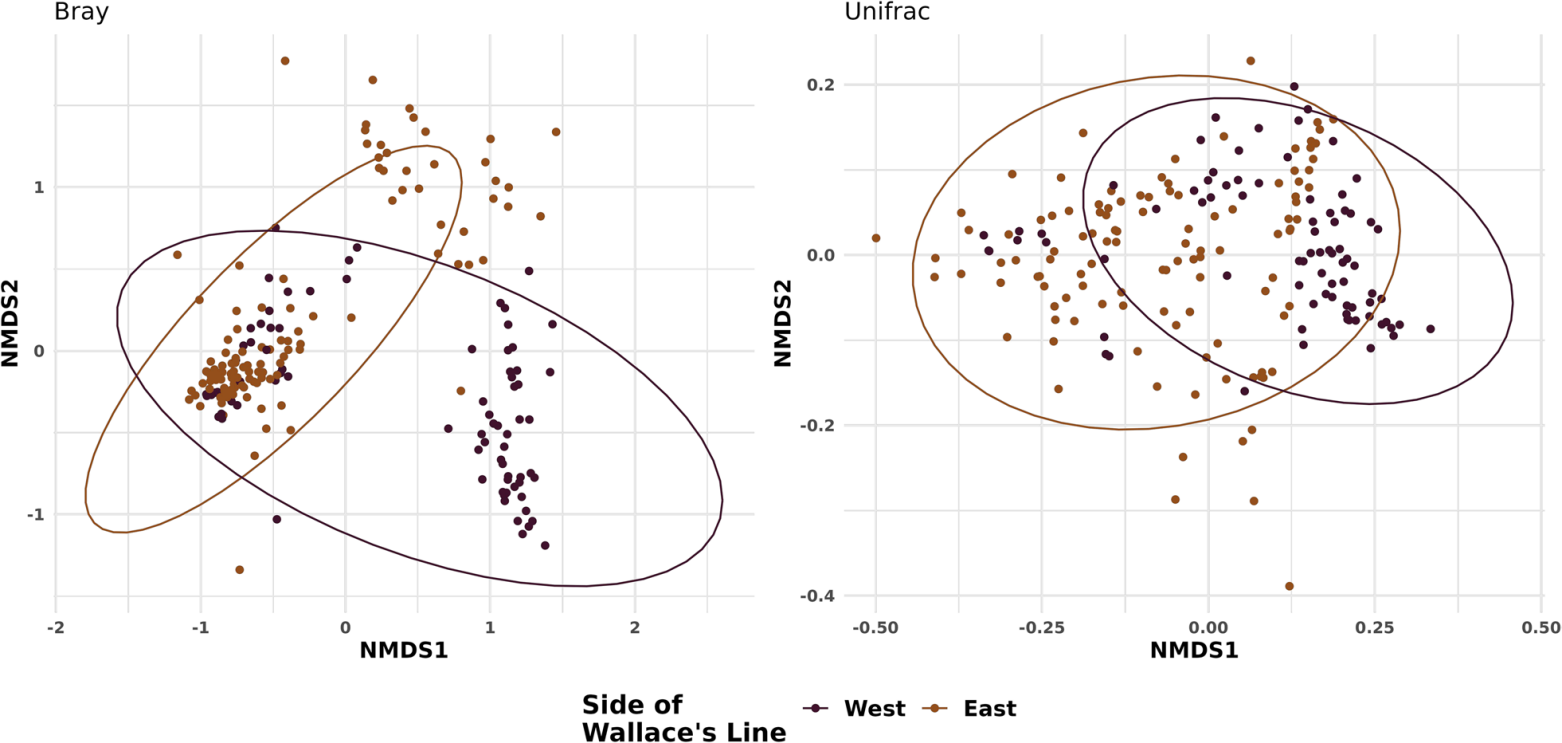
Ordination plots. Ordination plots for community structure of all samples using both Bray–Curtis (left panel) and UniFrac (right panel) dissimilarity indices. Samples are coloured by side of Wallace’s line (West = Purple; East = Orange)

Shannon diversity and richness estimates varied significantly by island (*P* < 0.001), but not across Wallace’s line (See Additional file 1: Appendix S1). The mean number of ASVs per sampling location was 198, with a minimum of 72 and a maximum of 478 (Fig. 2). Ordinations indicate that samples collected on either side of Wallace’s line tend to be more similar to each other. For example, samples from the East are more similar to other samples from the East in comparison to those from the West and vice versa (Fig. 3). This pattern was then confirmed by a PermANOVA test performed on the UniFrac community similarity metric. Community structure was significantly explained by location (*R*^2^ = 0.41; *P* = 0.001) and side of Wallace’s line (*R*^2^ = 0.11; *P* = 0.001; See Additional file 1: Appendix S2). Both the Mantel test and MRM found significant correlations between increasing spatial distance and community distance (*P* = 0.001 for both tests; See Additional file 1: Appendix S3).

### Differential abundance

**Fig. 4:**
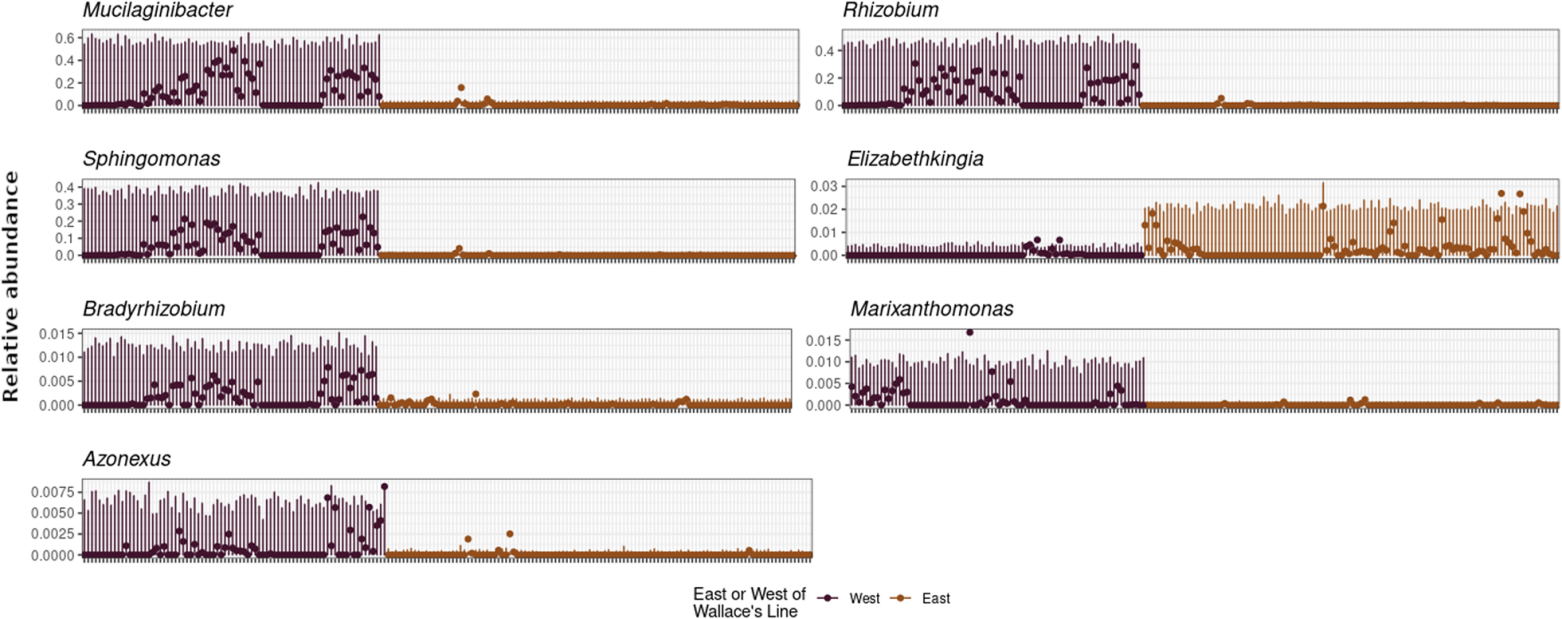
Differential abundance plots. Differential abundance and dispersion both East (Orange) and West (Purple) of Wallace’s Line. Points represent relative abundance of a given taxon in each sample. Bars represent dispersion estimates. Only taxa identified as significantly different between East and West by all four methods (corncob, indicspecies, random forest, and Sloan Neutral Model) are shown

Seven ASVs were found to be differentially abundant across Wallace’s line by all four methods used (Fig. 4; See Additional file 1: Appendix S4). Due to the stringent requirements that necessitated agreement between all four methods, these genera likely represent a conservative, but reliable estimate of those that are potentially differentiated across Wallace’s line. The Neutral Community Model analysis indicates that the relative abundances of these taxa differ significantly from the neutral model expectations (See Additional file 1: Appendix S4). The genera were *Mucilaginabacter*, *Sphingomonas*, *Bradyrhizobium*, *Marixanthomonas*, *Rhizobium*, *Elizabethkingia, and Azoneuxus.* The majority of these genera were preferentially found West of Wallace’s line with only *Elizabethkingia* being more abundant east of the line.

Taxa found to be differentially abundant across Wallace’s line belonged to genera that, based on BacDive records, have been shown to have significantly smaller minimum cell dimensions (0.8 µm) (GLM: t_923_ = 59.50, *P* < 0.001; See Additional file 1: Appendix S5) though the effect size was small. These same taxa belonged to genera that do not vary significantly in known cell shape (X-sq: df = 10, *P* = 0.675), or average surface area (GLM: t_928_ = − 0.31, *P* = 0.757) from all other taxa present.

## Discussion

Though not all ecological processes or physical phenomena that shape macroorganism biogeography can necessarily be usefully applied to microorganisms, this work shows that the cryptic biogeographic barrier known as Wallace's Line has a significant influence on the geographic structure of seagrass bacterial microbiota. Even though location was a stronger predictor of microbiota community structure, there was a significant distance-decay relationship between geography and community similarity, with a relatively large amount of community variation (11%) explained by Wallace’s line (See Appendices S2 & S3). We show that the microbiota of samples collected from either side of Wallace’s line are more similar to each other than they are to those collected on the opposite side. For example, the microbiota of the two closest islands on opposite sides of Wallace’s Line, Bali and Komodo, were more different from each other than either was to its most distant island on the same side of the line.

Previous work on microbial distributions across this line are sparse, but studies have shown that populations of the taxon *Burkholderia pseudomallei* can be differentiated on either side of the line, with two distinct subpopulations existing on opposing sides [62]. It is suggested that the complex geological and tectonic history of the region is responsible for these distributions, with the movement of tectonic plates isolating populations and facilitating genetic divergence. Other studies investigating liverworts and seagrass associated fungi across Wallace’s Line found that dispersal was also limited by this biogeographical boundary [18, 63]. Similar to ours, these finding suggest that even taxa with adaptions for long-distance dispersal may have difficulty crossing some biogeographic barriers. Additionally, other microbes have been found to follow this boundary, but they are obligate symbionts with host organisms that are themselves dispersal-limited by this line [64, 65]. Nevertheless, this still demonstrates a degree of microbial dispersal limitation.

The seagrass examined here, *S. isoetifolium*, is readily found throughout the entire Southeast Asian region and consequently it is abundant throughout the Indonesian Archipelago and on either side of Wallace’s Line [33]. Given this distribution, it is clear that bacterial microbiota are not just following the distribution of their host. Rather, it appears that dispersal across Wallace’s Line is limited. But, it should be noted that environmental conditions have been shown to influence seagrass associated microbiota in Southeast Asia [66–68]. Samples collected west of the line were made on the Sunda Shelf, where average water depths are approximately 70 m [69], whereas the region east of this line is dominated by deep water that can exceed 7000 m [70]. It is conceivable that these differences in environmental conditions may influence the observed differences in microbial community structure, but given that all collections were made in the top 5 m of water at all sites we think this is unlikely and the environment experienced at all locations is comparable and essentially homogeneous with respect to climate. Further supporting the idea that Wallace’s Line is a barrier to dispersal, we show that the microbiota from the two closest islands on opposite sides of the line, Bali and Komodo, are more different from each other than either was to its most distant island on the same side. However, future work could further examine the effects of additional environmental variables on microbial community structure.

### Traits of differentially abundant taxa

We identified seven ASVs that demonstrated statistically significant differential abundances in the seagrass microbiota on either side of the line (See Additional file 1: Appendix S4). Notably, the genera to which these ASVs were assigned have all been demonstrated to be non-spore forming [60]. Though we did not take any direct morphological measurements of cell isolates, using data on cell morphology (e.g., cell dimensions, surface area, and shape) extracted from the BacDive database [60], we note that the differentially abundant taxa on either side of Wallace’s Line belong to genera that have significantly smaller cell dimensions than those not differentially abundant on either side of the line, though the effect size was small (0.8um). This was unexpected since numerous studies suggest anything smaller than 1 mm is unlikely to show biogeographic patterns [71], and that for microscopic organisms dispersal is assumed to be rarely, if ever, limited by geographical barriers [72]. However, work in Southeast Asia examining mangrove [73, 74], seagrass [66, 75], and coral associated microbiota [76–78] shows that microbial biogeographic structure can exist over comparatively small spatial scales (< 6 km in some instances). Further supporting these observations, work by Jenkins et al., [79] similarly showed that a smaller size does not lead to further dispersal and a more cosmopolitan distribution. Additional work is needed to fully investigate the reasons behind this, but it is possible that smaller cells create less drag in an aqueous or atmospheric environment; consequently, they do not disperse as far as something that is larger and possibly has increased drag. Or, as a consequence of the smaller cell size, these cells have less energy reserves which limits their ability to disperse long distances before these reserves are fully utilised and death results.

Six out of the seven taxa with significantly differential abundance were more prevalent west of Wallace's Line (SI Table S5). *Mucilaginibacter* displayed the most striking difference across the line where it had a relative abundance of just over 20% west of the line, but less than 1% in the east.

The genus *Mucilaginibacter* has a non-motile and non-spore forming taxa that are found in both marine and fresh water environments [80]. Species from this genus have been identified as cellulose degraders [81] and potential plant pathogens [82]. *Sphingomonas* is a genus that contains motile species [60], this genus was a dominant community member west of Wallace’s Line. It has previously been associated with seagrasses where it was found to be dominant on healthy leaves [83] and species in this genus are thought to be major degraders of cellulose and play a significant role in carbon cycling [84]. Additionally this genus is known for its ability to degrade organometallic compounds [85]. Members of the genus *Bradyrhizobium* contain gram negative, non-spore forming, nitrogen-fixing species that have previously been isolated from seagrasses [60, 86]. It was found more frequently on the eastern side of Wallace’s Line in this study. *Marixanthomonas* contains gram negative, anaerobic, rod-shaped, non-motile, and non-spore forming species [60]. It has been isolated from tropical sediments, but little is known about any associations it forms with seagrasses. *Rhizobium* is another genus with gram negative, motile, non-spore forming species that had relative abundance of nearly 5% west of the line. Members of this genus have been identified as nitrogen fixers that are abundant in seagrass meadows, and it has been proposed as an indicator taxon that can be used to rapidly assess seagrass health, with the presence of *Rhizobium* thought to be indicative of healthy seagrass beds [86]. Members of the genus *Azonexus* have been shown to be Gram negative, non-spore forming, and highly motile [60]. They have possible roles in the production of plant growth promoting properties through the production of auxin, and have been identified as having roles in denitrification and nitrogen fixation [87]. The single taxon identified as more abundant East of Wallace’s Line belonged to *Elizabethkingia*, this taxa is ubiquitous in many human-associated aquatic environments, and it is an emerging opportunistic pathogen that is non-motile and can have detrimental consequences for human health [88–90]. *Elizabethkingia* outbreaks can be a consequence of contact with sewage and other untreated water sources [91].

Notable in the genera that represent these seven differentially abundant taxa is the lack of known spore formation; it is likely this lack of spore formation reduces long-distance dispersal viability. This is especially relevant to dispersal across Wallace’s Line as it follows the Indonesian throughflow (ITF) current. This current forms the only low latitude, warm water connection between the Indian and Pacific oceans and consequently this current moves a huge volume of water, estimated to be 10.5 × 10^6^ m^3^s^−1^ [92]. This formidable barrier will limit dispersal across it, and these dispersal limitations could be more apparent in taxa that do not produce spores and are therefore already more limited in dispersal ability in comparison to their spore forming counterparts.

Dispersal ability may not be the primary factor in determining the distribution of the *Elizabethkingia* genus. This genus has been associated with sewage and untreated wastewater [91]. At all of the sample sites located to the west of the line, rudimentary sanitation treatment facilities, pit latrines, or septic tanks existed. Whereas, east of this line, long drops directly above the water were the most frequently encountered form of sanitation. Considering the associations that *Elizabethkingia* has with sewage, the increased likelihood of human waste in the water resulting from the frequent use of long drops may in part be responsible for the increased prevalence of this genus on the eastern side of Wallace’s Line.

Similarly, the distribution of the genus *Rhizobium* may not be entirely a consequence of Wallace’s Line. This genus has been suggested as an indicator taxon associated with healthy seagrass meadows. Thus, if the increased abundance of *Elizabethkingia* to the east of Wallace’s Line is in fact a consequence of human waste, it is reasonable to hypothesize that eutrophication, one of the main drivers of seagrass loss [93], could be causing a decline in seagrass health and cover. These declines may be responsible for the lower prevalence of *Rhizobium* east of Wallace’s Line [87]. Much more work is required to confirm this trend, but it could in part be responsible for the observed distributions.

## Conclusions

With this work we show that seagrass associated microbiota are significantly different on either side of Wallace’s Line. We propose that dispersal limitations are one major driver of this difference. This reinvigorates questions about biogeographic barriers and the importance of dispersal barriers and environmental filtering across relatively short geographic distances. If cryptic dispersal barriers exist for bacteria in this region, this may have implications for the biogeography of macro-organisms that depend on these microbes. It also suggests that many broad-scale factors that are known to influence macroorganism distributions might also be shaping the distributions of microbiota in surprising ways.

### Supplementary Information

**Additional file 1**. Supplementary Material.

## Data Availability

All raw sequence data associated with this study is available in the Sequence Read Archive (SRA) under accession PRJNA944167. All sample metadata and fully documented analysis code is provided as a Zenodo release of the project GitHub repository [39].
