# Supplementary material for: Wallace’s line structures seagrass microbiota and is a potential barrier to the dispersal of marine bacteria
Source: Environ Microbiome. 2024 Apr 18;19:23. doi: 10.1186/s40793-024-00568-3 (PMC11027274; doi:10.1186/s40793-024-00568-3)
Supplement: Supplementary file 1 — Additional file 1. Supplementary Material. [file 40793_2024_568_MOESM1_ESM.html]

Supporting Information


- Appendix S1:
  Alpha-Diversity
- Appendix S2:
  Beta-Diversity
- Appendix S3: Distance
  Decay
- Appendix S4:
  Differential Abdundance
- Appendix S5:
  Bacterial morphology
  - Cell
    size
  - Cell
    shape
  - Cell morphology data
- Appendix S6: Sample
  metadata

# Supporting Information

---

# Appendix S1: Alpha-Diversity

```
##  [1] "Linear mixed model fit by REML. t-tests use Satterthwaite's method ['lmerModLmerTest']"                                           
##  [2] "Formula: Observed ~ east_west + (1 | island)"                                                                                     
##  [3] "   Data: alpha"                                                                                                                   
##  [4] ""                                                                                                                                 
##  [5] "REML criterion at convergence: 2213.6"                                                                                            
##  [6] ""                                                                                                                                 
##  [7] "Scaled residuals: "                                                                                                               
##  [8] "     Min       1Q   Median       3Q      Max "                                                                                    
##  [9] "-2.16837 -0.77293 -0.09447  0.57422  2.83527 "                                                                                    
## [10] ""                                                                                                                                 
## [11] "Random effects:"                                                                                                                  
## [12] " Groups   Name        Variance Std.Dev."                                                                                          
## [13] " island   (Intercept) 1619     40.23   "                                                                                          
## [14] " Residual             5859     76.54   "                                                                                          
## [15] "Number of obs: 192, groups:  island, 12"                                                                                          
## [16] ""                                                                                                                                 
## [17] "Fixed effects:"                                                                                                                   
## [18] "              Estimate Std. Error     df t value    Pr(>|t|)    "                                                                 
## [19] "(Intercept)     189.31      16.84  10.00  11.242 0.000000538 ***"                                                                 
## [20] "east_westWest    20.70      26.09  10.00   0.793       0.446    "                                                                 
## [21] "---"                                                                                                                              
## [22] "Signif. codes:  0 ‘***’ 0.001 ‘**’ 0.01 ‘*’ 0.05 ‘.’ 0.1 ‘ ’ 1"                                                                   
## [23] ""                                                                                                                                 
## [24] "Correlation of Fixed Effects:"                                                                                                    
## [25] "            (Intr)"                                                                                                               
## [26] "east_wstWst -0.645"                                                                                                               
## [27] "We fitted a linear mixed model (estimated using REML and nloptwrap optimizer) to predict Observed with east_west (formula:"       
## [28] "Observed ~ east_west). The model included island as random effect (formula: ~1 | island). The model's total explanatory power is" 
## [29] "moderate (conditional R2 = 0.23) and the part related to the fixed effects alone (marginal R2) is of 0.01. The model's intercept,"
## [30] "corresponding to east_west = East, is at 189.31 (95% CI [156.09, 222.53], t(188) = 11.24, p < .001). Within this model:"          
## [31] ""                                                                                                                                 
## [32] "  - The effect of east west [West] is statistically non-significant and positive (beta = 20.70, 95% CI [-30.76, 72.16], t(188) =" 
## [33] "0.79, p = 0.428; Std. beta = 0.24, 95% CI [-0.36, 0.85])"                                                                         
## [34] ""                                                                                                                                 
## [35] "Standardized parameters were obtained by fitting the model on a standardized version of the dataset. 95% Confidence Intervals"    
## [36] "(CIs) and p-values were computed using a Wald t-distribution approximation."
```

---

# Appendix S2: Beta-Diversity

### PermANOVA Tables:

#### Bray-Curtis Distance

SI Table 2. Bray-Curtis PermANOVA. Bold indicates P-value less than 0.05

| term | df | SumOfSqs | R2 | statistic | p.value |
| --- | --- | --- | --- | --- | --- |
| east\_west | 1 | 5.451782 | 0.0927267 | 32.14091 | 0.001 |
| location | 10 | 22.810467 | 0.3879722 | 13.44788 | 0.001 |
| Residual | 180 | 30.531824 | 0.5193011 | NA | NA |
| Total | 191 | 58.794072 | 1.0000000 | NA | NA |

#### UniFrac Distance

SI Table 3. UniFrac PermANOVA. Bold indicates P-value less than 0.05

| term | df | SumOfSqs | R2 | statistic | p.value |
| --- | --- | --- | --- | --- | --- |
| east\_west | 1 | 2.270203 | 0.1105812 | 42.06473 | 0.001 |
| location | 10 | 8.545070 | 0.4162288 | 15.83321 | 0.001 |
| Residual | 180 | 9.714469 | 0.4731900 | NA | NA |
| Total | 191 | 20.529742 | 1.0000000 | NA | NA |

---

### Beta dispersion:

```
## vegan::betadisper(d = UF, group = ps@sam_data$east_west, type = "centroid")
```

Table 4. Distance to centroid summaries for beta-dispersion.

| group | mean | sd |
| --- | --- | --- |
| East | 0.3090 | 0.0696 |
| West | 0.2853 | 0.0811 |

---

# Appendix S3: Distance Decay

### Multiple Regression on Matrices

SI Table 5. Call: MRM(asv\_dist ~ haversine\_dist,nperm=1000)

|  | coef.asv\_dist | coef.pval | r.squared | F.test |
| --- | --- | --- | --- | --- |
| Int | 0.694 | 1.000 | 0.048 | 919.5758 |
| haversine\_dist | 0.000 | 0.001 | 0.001 | 0.0010 |

### Mantel Test

SI Table 6. Mantel Test statistics.

| call | test.stat | P.value | N.perm |
| --- | --- | --- | --- |
| vegan::mantel(xdis = asv\_dist, ydis = haversine\_dist) | 0.2185436 | 0.001 | 999 |

### Distance decay plot

---

# Appendix S4: Differential Abdundance

### ASV-level

SI Table 7a. BBDML model estimates from all significant ASVs (including
ASV sequence).

| taxon | estimate | std\_error | t\_value | p\_value | ASV |
| --- | --- | --- | --- | --- | --- |
| Bacteria\_Proteobacteria\_Betaproteobacteria\_Burkholderiales\_Burkholderiaceae\_Burkholderia | 1.0456 | 0.2026 | 5.1617 | 0.0000 | TACGTAGGGTGCGAGCGTTAATCGGAATTACTGGGCGTAAAGCGTGCGCAGGCGGTTTGCTAAGACCGATGTGAAATCCCCGGGCTCAACCTGGGAACTGCATTGGTGACTGGCAGGCTAGAGTATGGCAGAGGGGGGTAGAATTCCACGTGTAGCAGTGAAATGCGTAGAGATGTGGAGGAATACCGATGGCGAAGGCAGCCCCCTGGGCCAATACTGACGCTCATGCACGAAAGCGTGGGGAGCAAACAGG |
| Bacteria\_Bacteroidetes\_Sphingobacteriia\_Sphingobacteriales\_Sphingobacteriaceae\_Mucilaginibacter | -1.3829 | 0.1780 | -7.7668 | 0.0000 | TACGGAGGATCCAAGCGTTATCCGGATTTATTGGGTTTAAAGGGTGCGTAGGTGGCTTTTTAAGTCAGGGGTGAAAGACGGCAGCTTAACTGTCGCAGTGCCCTTGATACTGAAGAGCTTGAATCCACTAGAGGTAGGCGGAATGTGACAAGTAGCGGTGAAATGCATAGATATGTCACAGAACACCAATTGCGAAGGCAGCTTACTATGGTGGTATTGACACTGAGGCACGAAAGCGTGGGGATCAAACAGG |
| Bacteria\_Proteobacteria\_Alphaproteobacteria\_Rhizobiales\_Rhizobiaceae\_Rhizobium | -1.3656 | 0.1859 | -7.3474 | 0.0000 | TACGAAGGGGGCTAGCGTTGTTCGGAATTACTGGGCGTAAAGCGCACGTAGGCGGATCGATCAGTCAGGGGTGAAATCCCAGGGCTCAACCCTGGAACTGCCTTTGATACTGTCGATCTGGAGTATGGAAGAGGTGAGTGGAATTCCGAGTGTAGAGGTGAAATTCGTAGATATTCGGAGGAACACCAGTGGCGAAGGCGGCTCACTGGTCCATTACTGACGCTGAGGTGCGAAAGCGTGGGGAGCAAACAGG |
| Bacteria\_Proteobacteria\_Alphaproteobacteria\_Sphingomonadales\_Sphingomonadaceae\_Sphingomonas | -1.4540 | 0.2051 | -7.0888 | 0.0000 | TACGGAGGGAGCTAGCGTTGTTCGGAATTACTGGGCGTAAAGCGCACGTAGGCGGCTTTGTAAGTCAGAGGTGAAAGCCTGGAGCTCAACTCCAGAACTGCCTTTGAGACTGCATCGCTTGAATCCAGGAGAGGTGAGTGGAATTCCGAGTGTAGAGGTGAAATTCGTAGATATTCGGAAGAACACCAGTGGCGAAGGCGGCTCACTGGACTGGTATTGACGCTGAGGTGCGAAAGCGTGGGGAGCAAACAGG |
| Bacteria\_Cyanobacteria/Chloroplast\_Cyanobacteria | -0.7081 | 0.1551 | -4.5653 | 0.0000 | TACGGGGGATGCAAGCGTTATCCGGAATCACTGGGCGTAAAGAGTCTGTAGGTGGCTTAACAAGTCTACTGTTAAATATTAAAGCTTAACTTTGAAACAGCAGTAGAAACTATTATGCTTGAGTATGGTAGGGGTAGAGGGAATTCCCAGTGTAGCGGTGAAATGCGTAGATATTGGGAAGAACACCGGTGGCGAAAGCGCTCTACTGGGCCATTACTGACACTCAGAGACGAAAGCTAAGGTAGCAAATGGG |
| Bacteria\_Proteobacteria\_Gammaproteobacteria\_Pseudomonadales\_Moraxellaceae\_Acinetobacter | 0.7958 | 0.1511 | 5.2664 | 0.0000 | TACAGAGGGTGCGAGCGTTAATCGGATTTACTGGGCGTAAAGCGTGCGTAGGCGGCTTTTTAAGTCGGATGTGAAATCCCCGAGCTTAACTTGGGAATTGCATTCGATACTGGGAAGCTAGAGTATGGGAGAGGATGGTAGAATTCCAGGTGTAGCGGTGAAATGCGTAGAGATCTGGAGGAATACCGATGGCGAAGGCAGCCATCTGGCCTAATACTGACGCTGAGGTACGAAAGCATGGGGAGCAAACAGG |
| Bacteria\_Bacteroidetes\_Flavobacteriia\_Flavobacteriales\_Flavobacteriaceae\_Aquimarina | -0.9188 | 0.2488 | -3.6928 | 0.0003 | TACGGAGGATCCAAGCGTTATCCGGAATCATTGGGTTTAAAGGGTCCGTAGGCGGGTTTATAAGTCAGTGGTGAAAGTTTTGGGCTCAACCGAAAAATTGCCATTGATACTGTAGATCTTGAATTATTGTGAAGTGGTTAGAATAAGTAGTGTAGCGGTGAAATGCATAGATATTACTTAGAATACCGATTGCGAAGGCAGATCACTAACAATTAATTGACGCTAAGGGACGAAAGCGTGGGTAGCGAACAGG |
| Bacteria\_Bacteroidetes\_Flavobacteriia\_Flavobacteriales\_Flavobacteriaceae\_Cloacibacterium | 0.6284 | 0.1382 | 4.5471 | 0.0000 | TACGGAGGGTGCAAGCGTTATCCGGATTTATTGGGTTTAAAGGGTCCGTAGGCGGACTTATAAGTCAGTGGTGAAAGCCTGTCGCTTAACGATAGAACTGCCATTGATACTGTAAGTCTTGAGTATATTTGAGGTAGCTGGAATAAGTAGTGTAGCGGTGAAATGCATAGATATTACTTAGAACACCAATTGCGAAGGCAGGTTACCAAGATATAACTGACGCTGAGGGACGAAAGCGTGGGGAGCGAACAGG |
| Bacteria\_Proteobacteria\_Gammaproteobacteria | -1.8277 | 0.5041 | -3.6259 | 0.0004 | AACGGGAAGGGTTAGCGTTATTTAAATTAACTGGGCGTAAAGAATACGTAGATGGGAAAATATTTAATTAAAAAAAAGTTAAATTTAACTTTAACATCTTTTTTTAAAATTTTTTTCTAGAGTTTAAAAGAAGATTATAGAACTTTAAATGTAACAGTAAAATGTATTGATATTTAAAAGAATTTCAAAAGCGAAAGCGATAATCTAAATTAAAACTGACATTGAGGTATTAAAGCATGGGTAGCCAAAGGG |
| Bacteria\_Actinobacteria\_Actinobacteria | -0.5942 | 0.2002 | -2.9677 | 0.0034 | GACGTAGGGACCGAGCGTTATCCGGATTTATTGGGCGTAAAGAGCTCGTAGGCGGTTGCGTAAGTCGGATGTGAAAACTTGAGGCTCAACCTCAAGACGCCATCCGATACTGCGCTGACTTGAATCCAGTAGGGGAGTGTGGAATTTGTTGTGTAGCGGTGAAATGCGCAGATATAACAAGGAACACCTATGGCGAAGGCAGCACTCTGGGCTGGTATTGACGCTGAGGAGCGAAAGCGTGGGTAGCAAACAGG |
| Bacteria\_Cyanobacteria/Chloroplast\_Cyanobacteria | 1.1111 | 0.2225 | 4.9943 | 0.0000 | TACGGGGGATGCAAGCGTTATCCGGAATCACTGGGCGTAAAGAGTCTGTAGGTGGTTTAAAAAGTCTGCTGTTAAATATTAAAGCTTAACTTTGAAAAGGCAGTGGAAACTATTATACTAGAGTATGGTAGGGGTAAAGGGAATTCCCAGTGTAGCGGTGAAATGCGTAGATATTGGGAAGAACACCAGTGGCGAAAGCGCTTTACTAGGCCATTACTGACACTCAGAGACGAAAGCTAAGGGAGCGAATGGG |
| Bacteria\_Proteobacteria\_Gammaproteobacteria\_Alteromonadales | -0.7515 | 0.2480 | -3.0300 | 0.0028 | TACGGAGGGTGCAAGCGTTAATCGGAATTACTGGGCGTAAAGCGCGCGTAGGCGGCTACTTAAGCTAGATGTGAAATCCCCGGGCTTAACCTGGGACGTGCATTTAGAACTGGGTAGCTAGAGTACAGAAGAGGAGTGTGGAATTTCAGGTGTAGCGGTGAAATGCGTAGAGATCTGAAGGAACACCAGTGGCGAAGGCGACACTCTGGTCTGATACTGACGCTGAGGTGCGAAAGCGTGGGGAGCAAACAGG |
| Bacteria\_Bacteroidetes\_Flavobacteriia\_Flavobacteriales\_Flavobacteriaceae\_Aquimarina | -1.7990 | 0.3616 | -4.9756 | 0.0000 | TACGGAGGATCCAAGCGTTATCCGGAATCATTGGGTTTAAAGAGTCCGTAGGCGGGTTTATAAGTCAGTGGTGAAAGTTTTGGGCTCAACCGAAAAATTGCCATTGATACTGTAGATCTTGAATTATTGTGAAGTGGTTAGAATAAGTAGTGTAGCGGTGAAATGCATAGATATTACTTAGAATACCGATTGCGAAGGCAGATCACTAACAATTAATTGACGCTAAGGGACGAAAGCGTGGGTAGCGAACAGG |
| Bacteria\_Proteobacteria\_Alphaproteobacteria\_Rhodobacterales\_Rhodobacteraceae\_Aliiroseovarius | -0.7273 | 0.1440 | -5.0489 | 0.0000 | TACGGAGGGGGTTAGCGTTGTTCGGAATTACTGGGCGTAAAGCGCGCGTAGGCGGATTAGTCAGTCAGAGGTGAAATCCCAGGGCTCAACCCTGGAACTGCCTTTGATACTGCTAGTCTTGAGTTCGAGAGAGGTGAGTGGAATTCCGAGTGTAGAGGTGAAATTCGTAGATATTCGGAGGAACACCAGTGGCGAAGGCGGCTCACTGGCTCGATACTGACGCTGAGGTGCGAAAGCGTGGGGAGCAAACAGG |
| Bacteria\_Proteobacteria\_Gammaproteobacteria\_Vibrionales\_Vibrionaceae\_Vibrio | 0.8459 | 0.2193 | 3.8579 | 0.0002 | TACGGAGGGTGCGAGCGTTAATCGGAATTACTGGGCGTAAAGCGCATGCAGGTGGTTTGTTAAGTCAGATGTGAAAGCCCGGGGCTCAACCTCGGAACTGCATTTGAAACTGGCAAACTAGAGTACTGTAGAGGGGGGTAGAATTTCAGGTGTAGCGGTGAAATGCGTAGAGATCTGAAGGAATACCAGTGGCGAAGGCGGCCCCCTGGACAGATACTGACACTCAGATGCGAAAGCGTGGGGAGCAAACAGG |
| Bacteria\_Proteobacteria\_Gammaproteobacteria | 1.0570 | 0.2572 | 4.1088 | 0.0001 | AACGAGAAGGGTTAGCGTTATTCAGATTAATTGGGCGTAAAGGATACGTAGGTTGAAAATTAATTAACTAAAAAAATTTTATGATTAATCCTAAAATATTTTTATTAAAAAATTTTTCTAGAGTTTAGATGAAGATTGTAGAACTTTAAATGTAACGGTAAAATGTGTTGATATTTAAAAGAATTTCAAAAGCGAAAGCAACAATCTAAACTAAAACTGACATTGAGGTATTAAAGCATGGGGAGCAAAGGGG |
| Bacteria\_Proteobacteria\_Alphaproteobacteria\_Rhodobacterales\_Rhodobacteraceae | -0.5893 | 0.1674 | -3.5196 | 0.0005 | TACGGAGGGGGTTAGCGTTGTTCGGAATTACTGGGCGTAAAGCGCACGTAGGCGGGCTGTTAAGTTAGAGGTGAAATCCCGGGGCTCAACCCCGGAACTGCCTTTAATACTGGCAGCCTAGAGTTCGAGAGAGGTGAGTGGAATTCCGAGTGTAGAGGTGAAATTCGTAGATATTCGGAGGAACACCAGTGGCGAAGGCGGCTCACTGGCTCGATACTGACGCTGAGGTGCGAAAGTGTGGGGAGCAAACAGG |
| Bacteria\_Bacteroidetes\_Flavobacteriia\_Flavobacteriales\_Flavobacteriaceae\_Aquimarina\_litoralis | 0.5907 | 0.1997 | 2.9588 | 0.0035 | TACGGAGGATCCAAGCGTTATCCGGAATCATTGGGTTTAAAGGGTCCGTAGGCGGGTTTGTAAGTCAGTGGTGAAAGTTTTCGGCTCAACCGGAAAATTGCCATTGATACTGCAAGTCTTGAATCATTATGAAGTGGTTAGAATAAGTAGTGTAGCGGTGAAATGCATAGATATTACTTAGAATACCGATTGCGAAGGCAGATCACTAATAATGTATTGACGCTAAGGGACGAAAGCGTGGGTAGCGAACAGG |
| Bacteria\_Proteobacteria\_Gammaproteobacteria\_Vibrionales\_Vibrionaceae\_Vibrio | 0.8613 | 0.2125 | 4.0527 | 0.0001 | TACGGAGGGTGCGAGCGTTAATCGGAATTACTGGGCGTAAAGCGCATGCAGGTGGTTTGTTAAGTCAGATGTGAAAGCCCGGGGCTCAACCTCGGAATAGCATTTGAAACTGGCAGACTAGAGTACTGTAGAGGGGGGTAGAATTTCAGGTGTAGCGGTGAAATGCGTAGAGATCTGAAGGAATACCGGTGGCGAAGGCGGCCCCCTGGACAGATACTGACACTCAGATGCGAAAGCGTGGGGAGCAAACAGG |
| Bacteria\_Cyanobacteria/Chloroplast\_Cyanobacteria\_Family\_IV\_GpIV | -0.8648 | 0.1971 | -4.3882 | 0.0000 | GACGGAGGATGCAAGCGTTATCCGGAATTATTGGGCGTAAAGCGTCCGTAGGCGGTTAGATAAGTCAGTTGTTAAAGACTGCAGCTCAACTGTGGGAGAGCAACTGAAACTGTTTGACTAGAGTATGGTAGGGGTAGAGGGAATTCCTAGTGTAGCGGTGAAATGCGTAGATATTAGGAAGAACACCAGTGGCGAAGGCGCTCTACTGGGCCAAGACTGACGCTGATGGACGAAAGCTAGGGGAGCGAAAGGG |
| Bacteria\_Cyanobacteria/Chloroplast\_Cyanobacteria | 0.6824 | 0.2156 | 3.1655 | 0.0018 | TACGGGGGATGCAAGCGTTATCCGGAATCACTGGGCGTAAAGAGTCTGTAGGTGGCTTAGTAAGTCTGCTGTTAAATATTAAAGCTTAACTTTGAAACAGCAGTAGAAACTGCTATGCTCGAGTATGGTAGGGGTAGAGGGAATTCCCAGTGTAGCGGTGAAATGCGTAGATATTGGGAAGAACACCAGTAGCGAAAGCGCTCTACTGGACCATTACTGACACTCAGAGACGAAAGCTAAGGTAGCGAATGGG |
| Bacteria\_Proteobacteria\_Gammaproteobacteria | 1.8473 | 0.5338 | 3.4608 | 0.0007 | AACGAGAAGGGTTAGCGTTATTCGAATTTATTGGGCGTAAAGGATACGTAGGTTGAAAATTAGTTAAATAAAAAAAAACTAAAATTAACTTTAGGTTATTTTTATTAAAAATTTTTTCTAGAGTTTAAATGAAGATTGTAGAACTTTAAATGTAACGGTAAAATGTATTGATATTTAAAAGAATTTCAAAAGCGAAAGCAACAATCTAAATTAAAACTGACATTGAGGTATTAAAGCATGGGTAGCAAAGGGG |
| Bacteria\_Bacteroidetes\_Flavobacteriia\_Flavobacteriales\_Flavobacteriaceae\_Aquimarina | -1.1512 | 0.3695 | -3.1160 | 0.0021 | TACGGAGGATCCAAGCGTTATCCGGAATCATTGGGTTTAAAGGGTCCGTAGGCGGGTCTGTAAGTCAGTGGTGAAAGTTTGCGGCTCAACCGTAAAATTGCCATTGATACTGCAGGTCTTGAATTATTGTGAAGTGGTTAGAATATGTAGTGTAGCGGTGAAATGCATAGATATTACATAGAATACCGATTGCGAAGGCAGATCACTAACAATGTATTGACGCTAAGGGACGAAAGCGTGGGTAGCGAACAGG |
| Bacteria\_Cyanobacteria/Chloroplast\_Cyanobacteria | -0.9096 | 0.2220 | -4.0968 | 0.0001 | TACGGAGGATGCAAGCGTTATTCGGAATTATTGGGCGTAAAGCGTCCGCAGGTGGTACTTCAAGTCTGCTGTCAAAGACCGAAGCTCAACTTCGGGCAGGCAGTGGAAACTGAAGAACTAGAGTACGGTAGGGGTAGAGGGAATTTCCAGTGTAGCGGTGAAATGCGTAGAGATTGGAAAGAACACCAGTGGCGAAGGCGCTCTACTGGACCTGTACTGACACTCAGGGACGAAAGCTAGGGTAGCGAAAGGG |
| Bacteria\_Proteobacteria\_Alphaproteobacteria\_Rhodobacterales\_Rhodobacteraceae\_Roseicyclus | 0.8882 | 0.2063 | 4.3057 | 0.0000 | TACGGAGGGGGCTAGCGTTGTTCGGAATTACTGGGCGTAAAGCGCGCGTAGGCGGGCTGTTAAGTCGGGGGTGAAATCCCGGGGCTCAACCCCGGAACTGCCCTCGATACTGGCAGCCTAGAGATCTGGAGAGGCGAGTGGAATTCCGAGTGTAGAGGTGAAATTCGTAGATATTCGGAGGAACACCAGTGGCGAAGGCGGCTCGCTGGCCAGATACTGACGCTGAGGCGCGAAAGCGTGGGGAGCAAACAGG |
| Bacteria\_Proteobacteria\_Alphaproteobacteria\_Caulobacterales\_Hyphomonadaceae\_Algimonas | -0.6718 | 0.2105 | -3.1917 | 0.0017 | TACGGAGGGGGCTAGCGTTGTTCGGAATTACTGGGCGTAAAGCGTGCGTAGGCGGATTGGAAAGTCAGATGTGAAATCCCGGGGCTCAACCCCGGAACTGCATTTGAAACTATCAGTCTAGAGTTCTGGAGAGGTAAGTGGAATTCCTAGTGTAGAGGTGAAATTCGTAGATATTAGGAGGAACACCAGAGGCGAAGGCGGCTTACTGGACAGATACTGACGCTGAGGCACGAAAGTGTGGGGAGCAAACAGG |
| Bacteria\_Proteobacteria\_Alphaproteobacteria\_Rhodobacterales\_Rhodobacteraceae | -1.2542 | 0.2434 | -5.1538 | 0.0000 | TACGGAGGGGGCTAGCGTTGTTCGGAATTACTGGGCGTAAAGCGCACGTAGGCGGACCAGTCAGTCAGAGGTGAAATCCCGGGGCTCAACCCCGGAACTGCCTTTGATACTGCTGGTCTGGAGTTCGAGAGAGGTGAGTGGAATTCCGAGTGTAGAGGTGAAATTCGTAGATATTCGGAGGAACACCAGTGGCGAAGGCGGCTCACTGGCTCGATACTGACGCTGAGGTGCGAAAGCGTGGGGAGCAAACAGG |
| Bacteria\_Bacteroidetes\_Flavobacteriia\_Flavobacteriales\_Flavobacteriaceae\_Tenacibaculum | -1.0489 | 0.2422 | -4.3305 | 0.0000 | TACGGAGGGTGCAAGCGTTATCCGGAATCATTGGGTTTAAAGGGTCCGCAGGCGGTCAATTAAGTCAGAGGTGAAATCCTGCAGCTTAACTGTAGAACTGCCTTTGATACTGGTTGACTTGAGTTATACGGAAGTAGGTAGAATAAGTAGTGTAGCGGTGAAATGCATAGATATTACTTAGAATACCGATTGCGAAGGCAGCCTACTACGTATATACTGACGCTCATGGACGAAAGCGTGGGGAGCGAACAGG |
| Bacteria\_Cyanobacteria/Chloroplast\_Cyanobacteria | -1.5311 | 0.4719 | -3.2447 | 0.0014 | TACGGGGGATGCAAGCGTTATCCGGAATTATTGGGCGTAAAGAGTCTGTAGGTTGTTTAGAAAGTCCATTGTTAAATATTAGAGCTCAACTCTAAACAAGCACTGGAAACTACTAGACTAGAGTATGGTAGGGGCAAAGGGAATTCCCAGTGTAGCGGTGAAATGCGTAGATATTGGGAAGAACACCAGAAGCGAAAGCGCTTTGCTGGGCCATTACTGACACTCAGAGACGAAAGCTAGGGGAGCGAATGGG |
| Bacteria\_Cyanobacteria/Chloroplast\_Cyanobacteria | 1.3578 | 0.4518 | 3.0055 | 0.0030 | TACGGGGGATGCAAGCGTTATCCGGAATCACTGGGCGTAAAGAGTCTGTAGGTGGCTTAACAAGTCTGCTGTTAAATATTAAAGCTTAACTTTGAAAAAGCAGTAGAAACTATTATGCTTGAGTATGGTAGGGGTAGAGGGAATTCCCAGTGTAGCGGTGAAATGCGTAGATATTGGGAAGAACACCGGTGGCGAAAGCGCTCTACTGGGCCATTACTGACACTCAGAGACGAAAGCTAAGGTAGCGAATGGG |
| Bacteria\_Bacteroidetes\_Sphingobacteriia\_Sphingobacteriales\_Saprospiraceae | -1.0502 | 0.2661 | -3.9465 | 0.0001 | TACGGAGGGTGCAAGCGTTATCCGGAATCACTGGGTTTAAAGGGTGCGTAGGCGGCTAGATAAGTCAGAGGTGAAAGTTTGCAGCTTAACTGTAAAATTGCCTTTGATACTGTTTAGCTTGAATTAGGTTGAGGTTAGCGGAATGTGACATGTAGCGGTGAAATGCATAGATATGTCATAGAACACCAATTGCGAAGGCAGCTGGCTAGACCTACATTGACGCTGAGGCACGAAAGCGTGGGGAGCGAACAGG |
| Bacteria\_Bacteroidetes\_Flavobacteriia\_Flavobacteriales\_Flavobacteriaceae\_Elizabethkingia | 1.6770 | 0.2860 | 5.8648 | 0.0000 | TACGGAGGGTGCAAGCGTTATCCGGATTTATTGGGTTTAAAGGGTCCGTAGGCGGACTGATAAGTCAGTGGTGAAATCCGACAGCTTAACTGTCGAACTGCCATTGATACTGTTAGTCTTGAGTAAGGTTGAAGTGGCTGGAATAAGTAGTGTAGCGGTGAAATGCATAGATATTACTTAGAACACCAATTGCGAAGGCAGGTCACTAAGTCTTAACTGACGCTGATGGACGAAAGCGTGGGGAGCGAACAGG |
| Bacteria\_Proteobacteria\_Alphaproteobacteria\_Rhodobacterales\_Rhodobacteraceae | -0.6468 | 0.2010 | -3.2173 | 0.0015 | TACGGAGGGGGTTAGCGTTGTTCGGAATTACTGGGCGTAAAGCGCACGTAGGCGGACTATTAAGTCAGGGGTGAAATCCCGGGGCTCAACCCCGGAACTGCCCTTGATACTGGTAGTCTAGAGTTCGAGAGAGGTGAGTGGAATTCCGAGTGTAGAGGTGAAATTCGTAGATATTCGGAGGAACACCAGTGGCGAAGGCGGCTCACTGGCTCGATACTGACGCTGAGGTGCGAAAGTGTGGGGAGCAAACAGG |
| Bacteria\_Proteobacteria\_Alphaproteobacteria\_Rhodobacterales\_Rhodobacteraceae | -0.6839 | 0.2130 | -3.2113 | 0.0016 | TACGGAGGGGGTTAGCGTTGTTCGGAATTACTGGGCGTAAAGCGCACGTAGGCGGATTAGAAAGTTGGGGGTGAAATCCCGGAGCTCAACTCCGGAACTGCCTTCAAAACTCCTAGTCTAGAGTTCGAGAGAGGTGAGTGGAATTCCGAGTGTAGAGGTGAAATTCGTAGATATTCGGAGGAACACCAGTGGCGAAGGCGGCTCACTGGCTCGATACTGACGCTGAGGTGCGAAAGTGTGGGGAGCAAACAGG |
| Bacteria\_Bacteroidetes\_Flavobacteriia\_Flavobacteriales\_Flavobacteriaceae\_Dokdonia | -0.6378 | 0.1787 | -3.5686 | 0.0005 | TACGGAGGGTGCAAGCGTTATCCGGAATCATTGGGTTTAAAGGGTCCGTAGGCGGGCATATAAGTCAGTGGTGAAAGTCTGCAGCTTAACTGTAGAACTGCCATTGATACTGTATGTCTTGAATTATTGTGAAGTGGTTAGAATATGTAGTGTAGCGGTGAAATGCTTAGAGATTACATAGAATACCGATTGCGAAGGCAGATCACTAACAATACATTGACGCTGATGGACGAAAGCGTAGGTAGCGAACAGG |
| Bacteria\_Bacteroidetes\_Sphingobacteriia\_Sphingobacteriales\_Saprospiraceae\_Phaeodactylibacter | -0.6792 | 0.1995 | -3.4040 | 0.0008 | TACGGAGGGTGCAAGCGTTATCCGGAATCACTGGGTTTAAAGGGTGCGTAGGCGGCGCTATAAGTCAGGAGTGAAATCCCTCAGCTAAACTGGGGAACTGCTTTTGATACTGTAGTGCTTGAATCAGGCTGAGGTCAGCGGAATGAGTCATGTAGCGGTGAAATGCATAGATATGACTTAGAACACCAATTGCGAAGGCAGCTGGCTAGACCTGTATTGACGCTGAGGCACGAAAGCGTGGGGAGCGAACAGG |
| Bacteria\_Cyanobacteria/Chloroplast\_Cyanobacteria\_Family\_IV\_GpIV | -1.0689 | 0.3441 | -3.1063 | 0.0022 | TACGGAGGATGCAAGCGTTATCCGGAATTATTGGGCGTAAAGCGTCCGCAGGTGGTTAGTCAAGTCAGCTGTAAAAGACTCAGGCTTAACTTGGGATGTGCAGTTGAAACTGATTGACTAGAGTACGGTAGGGGTAGAGGGAATTCCTAGTGTAGCGGTGAAATGCGTAGATATTAGGAAGAACACCAGTGGCGAAGGCGCTCTACTGGGCCTGTACTGACACTGAGGGACGAAAGCTAGGGGAGCGAAAGGG |
| Bacteria\_Bacteroidetes\_Sphingobacteriia\_Sphingobacteriales\_Saprospiraceae | -0.6550 | 0.2257 | -2.9020 | 0.0041 | TACGGAGGGTGCAAGCGTTATCCGGAATTACTGGGTTTAAAGGGTGCGTAGGCGGTTTAGTAAGTCAGAAGTGAAAAGTCCGGGCTCAACCGGGGCCTTGCTTTTGATACTGCTAGACTTGAAAGGGGCTGAGGTCAGCGGAATGTGACATGTAGCGGTGAAATGCATAGATATGTCATAGAACACCAATTGCGAAGGCAGCTGGCTAGACCTACTTTGACGCTGAGGCACGAAAGCGTGGGGAGCGAACAGG |
| Bacteria\_Proteobacteria\_Alphaproteobacteria\_Rhodobacterales\_Rhodobacteraceae | 0.9831 | 0.2209 | 4.4509 | 0.0000 | TACGGAGGGGGCTAGCGTTGTTCGGAATTACTGGGCGTAAAGCGCGCGTAGGCGGGCTGTTAAGTCGGGGGTGAAATCCCGGGGCTCAACCCCGGAACTGCCCTCGATACTGGCAGCCTAGAGGATGAGAGAGGCGAGTGGAATACCGAGTGTAGAGGTGAAATTCGTAGATATTCGGTGGAACACCAGTGGCGAAGGCGGCTCGCTGGCTCATTTCTGACGCTGAGGTGCGAAAGCGTGGGGAGCAAACAGG |
| Bacteria\_Bacteroidetes\_Flavobacteriia\_Flavobacteriales\_Flavobacteriaceae\_Dokdonia | -0.9882 | 0.2363 | -4.1820 | 0.0000 | TACGGAGGGTGCAAGCGTTATCCGGAATCATTGGGTTTAAAGGGTCCGTAGGCGGGTTTATAAGTCAGTGGTGAAAGTCTGCAGCTTAACTGTAGAATTGCCATTGATACTGTAGATCTTGAATTATTGTGAAGTGGTTAGAATATGTAGTGTAGCGGTGAAATGCTTAGAGATTACATAGAATACCGATTGCGAAGGCAGATCACTAACAATATATTGACGCTGATGGACGAAAGCGTAGGTAGCGAACGGG |
| Bacteria\_Cyanobacteria/Chloroplast\_Cyanobacteria | 2.0090 | 0.5297 | 3.7927 | 0.0002 | TACGGGGGATGCAAGCGTTATCCGGAATCACTGGGCGTAAAGAGTCTGTAGGTGGCTTAATAAGTCTGCTGTTAAATATTAAAGCTTAACTTTGAAACAGCAGTAGAAACTGTTAGGCTTGAGTATGGTAGGGGTAGAGGGAATTCCCAGTGTAGCGGTGAAATGCGTAGATATTGGGAAGAACACCAGTGGCGAAAGCGCTCTACTAGGCCATAACTGACACTCAGAGACGAAAGCTAAGGTAGCGAATGGG |
| Bacteria\_Proteobacteria\_Gammaproteobacteria\_Gammaproteobacteria\_incertae\_sedis\_Candidatus\_Carsonella | 3.8249 | 1.0113 | 3.7823 | 0.0002 | AACGAGAAGGGTTAGCGTTATTCGAATTTATTGGGCGTAAAGGATACGTAGGTTGAAAATTAGTTAAATAAAAAAAAGCTAAAGTTAATTTTAGATTATTTTTATTAAAAATTTTTTCTAGAGTTTAAACGAAGATTGTAGAACTTTAAATGTAACGGTAAAATGTATTGATATTTAAAAGAATTTCAAAAGCGAAAGCAACAATCTATGTTAAAACTGACATTGAGGTATTAAAGCATGGGGAGCAAAGGGG |
| Bacteria\_Bacteroidetes\_Sphingobacteriia\_Sphingobacteriales\_Saprospiraceae\_Phaeodactylibacter | -0.7024 | 0.2162 | -3.2486 | 0.0014 | TACGGAGGGTGCAAGCGTTATCCGGAATCACTGGGTTTAAAGGGTGCGTAGGCGGATAGATAAGTCAGAGGTGAAAGGTGGTCGCTTAACGATCAAATTGCCTTTGATACTGTTTATCTTGAATCAAGTTGAGGTTGGCGGAATGAGTCATGTAGCGGTGAAATGCATAGATATGACTTAGAACACCAATTGCGAAGGCAGCTGGCTAGACTTGTATTGACGCTGAGGCACGAAAGCGTGGGGAGCGAACAGG |
| Bacteria\_Proteobacteria\_Alphaproteobacteria\_Rhodobacterales\_Rhodobacteraceae\_Pseudoruegeria | -0.9084 | 0.2244 | -4.0482 | 0.0001 | TACGGAGGGGGTTAGCGTTGTTCGGAATTACTGGGCGTAAAGCGCGCGTAGGCGGACTATTAAGTCGAGGGTGAAATCCCGGGGCTCAACCCCGGAACTGCCTTCGATACTGGTAGTCTTGAGTTCGAGAGAGGTGAGTGGAATTCCGAGTGTAGAGGTGAAATTCGTAGATATTCGGAGGAACACCAGTGGCGAAGGCGGCTCACTGGCTCGATACTGACGCTGAGGTGCGAAAGTGTGGGGAGCAAACAGG |
| Bacteria\_Bacteroidetes\_Sphingobacteriia\_Sphingobacteriales\_Saprospiraceae\_Lewinella | -2.1888 | 0.4868 | -4.4965 | 0.0000 | TACGGAGGGTGCAAGCGTTATCCGGAATCACTGGGTTTAAAGGGTGCGTAGGCGGGTTGATAAGTCAGAGGTGAAAGGCTACAGCTTAACTGTGGGACTGCCTTTGATACTGTTGATCTTGAATTAGGTTGAGGTTGGCGGAATGTGACAAGTAGCGGTGAAATGCATAGATATGTCATAGAACACCAATTGCGAAGGCAGCTGACTAGACCTATATTGACGCTGAGGCACGAAAGCGTGGGGAGCGAACAGG |
| Bacteria\_Proteobacteria\_Betaproteobacteria\_Burkholderiales\_Comamonadaceae\_Comamonas | 2.0657 | 0.4023 | 5.1343 | 0.0000 | TACGTAGGGTGCAAGCGTTAATCGGAATTACTGGGCGTAAAGCGTGCGCAGGCGGTTTTGTAAGACAGTGGTGAAATCCCCGGGCTCAACCTGGGAACTGCCATTGTGACTGCAAGGCTGGAGTGCGGCAGAGGGGGATGGAATTCCGCGTGTAGCAGTGAAATGCGTAGATATGCGGAGGAACACCGATGGCGAAGGCAATCCCCTGGGCCTGCACTGACGCTCATGCACGAAAGCGTGGGGAGCAAACAGG |
| Bacteria\_Proteobacteria\_Alphaproteobacteria\_Rhodobacterales\_Rhodobacteraceae\_Celeribacter | 0.9998 | 0.2827 | 3.5368 | 0.0005 | TACGGAGGGGGTTAGCGTTGTTCGGAATTACTGGGCGTAAAGCGCACGTAGGCGGATTAGTCAGTCAGAGGTGAAATCCCGGAGCTCAACTCCGGAACTGCCTTTGATACTGCTAGTCTTGAGTTCGAGAGAGGTAAGTGGAATTCCGAGTGTAGAGGTGAAATTCGTAGATATTCGGAGGAACACCAGTGGCGAAGGCGGCTTACTGGCTCGATACTGACGCTGAGGTGCGAAAGTGTGGGGAGCAAACAGG |
| Bacteria\_Proteobacteria\_Gammaproteobacteria\_Gammaproteobacteria\_incertae\_sedis | -1.0014 | 0.3014 | -3.3224 | 0.0011 | TACGGAGGGTGCAAGCGTTAATCGGAATTACTGGGCGTAAAGCGCGCGTAGGCGGCTTCGTCAGTCAGATGTGAAAGCCCAGGGCTCAACCTTGGAATTGCATTTGATACTGCGAGGCTAGAGTATGGTAGAGGGGAGTGGAATTTCCAGTGTAGCGGTGAAATGCGTAGATATTGGAAGGAACACCAGTGGCGAAGGCGACTCCCTGGGCCAATACTGACGCTGAGGTGCGAAAGCGTGGGGAGCAAACAGG |
| Bacteria\_Proteobacteria\_Gammaproteobacteria\_Alteromonadales\_Alteromonadales\_incertae\_sedis\_Teredinibacter | 1.6884 | 0.2959 | 5.7066 | 0.0000 | TACGAGGGGTGCAAGCGTTAATCGGAATTACTGGGCGTAAAGCGCGCGTAGGCGGTTATCTAAGCTAGATGTGAAATCCCAGGGCTTAACCTTGGAACTGCATTTAGAACTGGGTAGCTAGAGTACAGCAGAGGATAGTGGAATTTCAGGTGTAGCGGTGAAATGCGTAGAGATCTGAAGGAACATCAGTGGCGAAGGCGACTGTCTGGGCTGATACTGACGCTGAGGTGCGAAAGCGTGGGGAGCAAACAGG |
| Bacteria\_Bacteroidetes\_Flavobacteriia\_Flavobacteriales\_Flavobacteriaceae\_Tenacibaculum | -1.2500 | 0.3638 | -3.4365 | 0.0007 | TACGGAGGGTGCAAGCGTTATCCGGAATCATTGGGTTTAAAGGGTCCGCAGGCGGTCAATTAAGTCAGAGGTGAAATCCTACAGCTTAACTGTAGAACTGCCTTTGATACTGGTTGACTTGAGTTATACGGAAGTAGGTAGAATGTGTAGTGTAGCGGTGAAATGCATAGATATTACACAGAATACCGATTGCGAAGGCAGCCTACTACGTATATACTGACGCTCATGGACGAAAGCGTGGGGAGCGAACAGG |
| Bacteria\_Proteobacteria\_Alphaproteobacteria\_Rhodobacterales\_Rhodobacteraceae | -0.7310 | 0.2392 | -3.0557 | 0.0026 | TACGGAGGGGGTTAGCGTTGTTCGGAATTACTGGGCGTAAAGCGCGCGTAGGCGGACTGGAAAGTGTGGGGTGAAATCCCGGGGCTCAACCCCGGAACTGCCCTGCAAACTATCAGTCTAGAGTTCGAGAGAGGTGAGTGGAATTCCGAGTGTAGAGGTGAAATTCGTAGATATTCGGAGGAACACCAGTGGCGAAGGCGGCTCACTGGCTCGATACTGACGCTGAGGTGCGAAAGTGTGGGGAGCAAACAGG |
| Bacteria\_Proteobacteria\_Alphaproteobacteria\_Rhizobiales\_Bradyrhizobiaceae\_Bradyrhizobium | -2.7336 | 0.4367 | -6.2604 | 0.0000 | TACGAAGGGGGCTAGCGTTGCTCGGAATCACTGGGCGTAAAGGGTGCGTAGGCGGGTCTTTAAGTCAGGGGTGAAATCCTGGAGCTCAACTCCAGAACTGCCTTTGATACTGAAGATCTTGAGTTCGGGAGAGGTGAGTGGAACTGCGAGTGTAGAGGTGAAATTCGTAGATATTCGCAAGAACACCAGTGGCGAAGGCGGCTCACTGGCCCGATACTGACGCTGAGGCACGAAAGCGTGGGGAGCAAACAGG |
| Bacteria\_Cyanobacteria/Chloroplast\_Cyanobacteria | -1.3932 | 0.4787 | -2.9107 | 0.0040 | TACGGGGGATGCAAGCGTTATCCGGAATCACTGGGCGTAAAGAGTCTGTAGGTGGCTCAATAAGTCTGCTGTTAAATATTAAAGCTTAACTTTAAACAAGCGGTAGAAACTATTGTGCTAGAGTATGGTAGGGGTAGAGGGAATTCCCAGTGTAGCGGTGAAATGCGTAGATATTGGGAAGAACACCAGTGGCGAAAGCGCTCTACTGGGCCATTACTGACACTCAGAGACGAAAGCTAAGGTAGCGAATGGG |
| Bacteria\_Bacteroidetes\_Sphingobacteriia\_Sphingobacteriales\_Saprospiraceae | -0.7010 | 0.2324 | -3.0162 | 0.0029 | TACGGAGGGTGCAAGCGTTATCCGGAATCACTGGGTTTAAAGGGTGCGTAGGCGGTCCGACAAGTCAGAGGTGAAATGCACGAGCTTAACTGGTGAATTGCCTTTGAAACTGTCGAGCTAGAATTATGTTGAGGTTAGCGGAATGAGTCATGTAGCGGTGAAATGCATAGATATGACTTAGAACACCAATTGCGTAGGCAGCTAGCTAGGCATCAATTGACGCTGAGGCACGAAAGCGTGGGGAGCGAACAGG |
| Bacteria\_Bacteroidetes\_Cytophagia\_Cytophagales | -1.3960 | 0.3740 | -3.7328 | 0.0003 | TACGTAGGGTGCAAGCGTTGTCCGGATTTATTGGGTTTAAAGGGTGCGCAGGCGGCCTTTTAAGTTAGTGGTGAAAGCGTCCAGCTTAACTGGATATCCGCCATTGATACTGGAAGGCTTGAGTCCGTCTGAGGAGATTAGAATGCGTGGTGTAGCGGTGAAATGCATAGATATCACGCAGAATACCGATTGCGAAGGCAGATCTCTAAGGCGGAACTGACGCTGAGGCACGAAAGCGTGGGTAGCGAACAGG |
| Bacteria\_Proteobacteria\_Alphaproteobacteria\_Rhodobacterales\_Rhodobacteraceae\_Thalassobius | -0.9228 | 0.2938 | -3.1405 | 0.0020 | TACGGAGGGGGTTAGCGTTGTTCGGAATTACTGGGCGTAAAGCGCGCGTAGGCGGATTAGTAAGTTAGAGGTGAAATCCCGGGGCTCAACCCCGGAACTGCCTTTAATACTGCTAGTCTTGAGTTCGAGAGAGGTGAGTGGAATTCCGAGTGTAGAGGTGAAATTCGTAGATATTCGGAGGAACACCAGTGGCGAAGGCGGCTCACTGGCTCGATACTGACGCTGAGGTGCGAAAGTGTGGGGAGCAAACAGG |
| Bacteria\_Proteobacteria\_Gammaproteobacteria | 1.8426 | 0.4397 | 4.1907 | 0.0000 | TACGGAGGGTGCGAGCGTTAATCGGAATTACTGGGCGTAAAGCGCACGTAGGTGGTTAATTAAGCGAGGTGTGAAATCCCCGGGCTCAACCTGGGAACTGCACTTCGAACTGGTTAACTAGAGTATGGTAGAGGATAGTGGAATTTCAGGTGTAGCGGTGAAATGCGTAGAGATCTGAAGGAACATCAGTGGCGAAGGCGACTGTCTGGACTAATACTGACACTGAGGTGCGAAAGCGTGGGTAGCAAACAGG |
| Bacteria\_Proteobacteria\_Gammaproteobacteria | 2.2573 | 0.4706 | 4.7966 | 0.0000 | TACGGAGGGTGCAAGCGTTAATCGGAATTACTGGGCGTAAAGCGCACGCAGGTGGAATAGTAAGTTGGATGTGAAAGCCCTGGGCTCAACCTAGGAACTGCATTCAAAACTGCTAATCTAGAGTACAGGAGAGGGAAGTGGAATTTCCGGTGTAGCGGTGAAATGCGTAGATATCGGAAGGAACAACAGTGGCGAAGGCGACTTCCTGGACTGATACTGACACTCAGGTGCGAAAGCGTGGGGAGCAAACAGG |
| Bacteria\_Proteobacteria\_Alphaproteobacteria\_Rhodobacterales\_Rhodobacteraceae\_Roseivivax | 1.4658 | 0.3227 | 4.5423 | 0.0000 | TACGGAGGGGGTTAGCGTTGTTCGGAATTACTGGGCGTAAAGCGCGCGTAGGCGGATTGGAAAGTTGGGGGTGAAATCCCGGGGCTCAACCCCGGAACTGCCTTCAAAACTCCCAGTCTAGAGTTCGAGAGAGGTGAGTGGAATTCCGAGTGTAGAGGTGAAATTCGTAGATATTCGGAGGAACACCAGTGGCGAAGGCGGCTCACTGGCTCGATACTGACGCTGAGGTGCGAAAGTGTGGGGAGCAAACAGG |
| Bacteria\_Cyanobacteria/Chloroplast\_Cyanobacteria\_Family\_V\_GpV | -1.0667 | 0.3717 | -2.8700 | 0.0046 | TACGAGGGATGCAAGCGTTATCCGGAATTATTGGGCGTAAAGCGTTCGTAGGTGGCTAGGTAAGTCTGTCGTTAAAGCGTGGAGCTTAACTCCATAACGGCGGTGGAAACTGCCTGGCTAGAGTATGGTAGGGGTAACAGGAATTCCCAGTGTAGCGGTGAAATGCGTAGATATTGGGAAGAACACCAGCGGCGAAAGCGTGTTACTGGGCCATTACTGACACTGAGGGACGAAAGCTAGGGGAGCGAAAGGG |
| Bacteria\_Cyanobacteria/Chloroplast\_Cyanobacteria\_Family\_I\_GpI | -1.2417 | 0.4247 | -2.9238 | 0.0039 | TACGGAGGATGCAAGCGTTATCCGGAATAATTGGGCGTAAAGCGTTCGTAGGTGGTTTTGTAAGTCTGCTGTTAAAGCGTGTAGCTTAACTACATATAGGCAGTGGAAACTACAAAACTTGAGTGCGTTCGGGGTAGAGGGAATTCCTGGTGTAGCGGTGAAATGCGTAGATATCAGGAAGAACACCGGTGGCGAAGGCGCTCTACTAGGCCGTAACTGACACTGAGGGACGAAAGCTAGGGGAGCGAATGGG |
| Bacteria\_Deinococcus-Thermus\_Deinococci\_Deinococcales\_Trueperaceae\_Truepera | -1.3715 | 0.3313 | -4.1404 | 0.0001 | TACAGAGGGTGCGAGCGTTATCCGGAATCACTGGGCGTAAAGGGCACGTAGGCGGTTTGTTAAGTCCGATGTTAAAGCGTGAGGCTCAACCTCATCACGGCGTTGGATACTGGCAAACTTGACTTCTGGAGAGGAAAGTAGAATTACCAGTGTAGCGGTGAAATGCGTAGATACTGGTAGGAATACCCATTGCGAAGGCAGCTTTCTGGACAGATAGTGACGCTGAGGTGCGAAAGTGTGGGGAGCAAACCGG |
| Bacteria\_Bacteroidetes\_Cytophagia\_Cytophagales\_Cytophagaceae\_Ekhidna | -1.1811 | 0.3661 | -3.2257 | 0.0015 | TACGGAGGGTGCAAGCGTTGTCCGGATTTATTGGGTTTAAAGGGTACGTAGGCGGATTTTTAAGTCCGTGGTGAAAGCCTACAGCTTAACTGTAGAACTGCCATGGATACTGGAGATCTTGAATTCAGTTGAGGTAAGCGGAATTTATGATGTAGCGGTGAAATGCATAGATATCATAAAGAACACCTATTGCGAAGGCAGCTTGCTGGACTTGGATTGACGCTAATGTACGAAAGCGTGGGGAGCGAACAGG |
| Bacteria\_Proteobacteria\_Alphaproteobacteria\_Caulobacterales\_Hyphomonadaceae\_Algimonas | -2.6269 | 0.5312 | -4.9454 | 0.0000 | TACGGAGGGGGCTAGCGTTGTTCGGAATTACTGGGCGTAAAGCGTGCGTAGGCGGATTGGAAAGTTAGGTGTGAAATCCCGGGGCTCAACCCCGGAACTGCACTTAAAACTACCAGTCTAGAGTTCTGGAGAGGTAAGTGGAATTCCTAGTGTAGAGGTGAAATTCGTAGATATTAGGAGGAACACCAGAGGCGAAGGCGGCTTACTGGACAGATACTGACGCTGAGGCACGAAAGTGTGGGGAGCAAACAGG |
| Bacteria\_Bacteroidetes\_Sphingobacteriia\_Sphingobacteriales\_Saprospiraceae\_Phaeodactylibacter | -0.9646 | 0.3257 | -2.9617 | 0.0035 | TACGGAAGGTGCAAGCGTTATCCGGAATCACTGGGTTTAAAGGGTGCGTAGGCGGGACGATAAGTCAGAAGTGAAAGGTACCAGCTTAACTGGTAAATTGCTTTTGATACTGTTGTTCTTGAAACAGGTTGAGGTCGGCGGAATGTGGCATGTAACGGTGAAATGTGTAGATATGCCATAGAACACCAATTGCGAAGGCAGCTGGCTAGACCTAGTTTGACGCTGAGGCACGAAAGCGTGGGGAGCGAACAGG |
| Bacteria\_Proteobacteria\_Alphaproteobacteria\_Rhodobacterales\_Rhodobacteraceae | -0.9325 | 0.2666 | -3.4972 | 0.0006 | TACGGAGGGGGCTAGCGTTGTTCGGAATTACTGGGCGTAAAGCGCACGTAGGCGGACCGATCAGTCAGAGGTGAAATCCCAGGGCTCAACCCTGGAACTGCCTTTGATACTGTCGGTCTGGAGTTCGAGAGAGGTGAGTGGAATTCCGAGTGTAGAGGTGAAATTCGTAGATATTCGGAGGAACACCAGTGGCGAAGGCGGCTCACTGGCTCGATACTGACGCTGAGGTGCGAAAGCGTGGGGAGCAAACAGG |
| Bacteria\_Proteobacteria\_Gammaproteobacteria | -2.2415 | 0.4854 | -4.6181 | 0.0000 | TACGGAGGGTGCAAGCGTTAATCGGAATTACTGGGCGTAAAGCGCGCGTAGGTGGTTTTGTAAGTTGGATGTGAAAGCCCTGGGCTCAACCTGGGAACTGCATTCAAAACTGCATCACTAGAGTATGGGAGAGGGAAGTGGAATTTCCGGTGTAGCGGTGAAATGCGTAGAGATCGGAAGGAACACCAATGGCGAAGGCAACTTCCTGGACCAATACTGACACTGAGGTGCGAAAGCGTGGGGAGCAAACAGG |
| Bacteria\_Bacteroidetes\_Flavobacteriia\_Flavobacteriales\_Flavobacteriaceae\_Aureitalea | -2.1108 | 0.6291 | -3.3551 | 0.0010 | TACGGAGGATGCAAGCGTTATCCGGAATCATTGGGTTTAAAGGGTCCGTAGGCGGATAATTAAGTCAGAGGTGAAAGTTTGCGGCTCAACCGTAAAATTGCCTTTGATACTGGTTGTCTTGAATTACTGTGAAGTGGTTAGAATATGTAGTGTAGCGGTGAAATGCATAGATATTACATAGAATACCAATTGCGAAGGCAGATCACTAACAGTATATTGACGCTGAGGGACGAAAGCGTGGGGAGCGAACAGG |
| Bacteria\_Proteobacteria\_Alphaproteobacteria\_Rhodospirillales\_Rhodospirillaceae\_Rhodospirillum | 3.1936 | 1.0178 | 3.1376 | 0.0020 | TACGAAGGGGGCAAGCGTTGTTCGGAATCACTGGGCGTAAAGAGCGCGTAGGCGGTTTGATTAGTCAGAGGTGAAAGCCCAGAGCTCAACTTTGGAATAGCCTTTGATACTGTCAGACTTGAGTTCGTGAGAGGGTGGTGGAATTCCCAGTGTAGAGGTGAAATTCGTAGATATTGGGAGGAACACCAGTGGCGAAGGCGGCCACCTGGCGCGATACTGACGCTGAGGCGCGAAAGCGTGGGGAGCAAACAGG |
| Bacteria\_Bacteroidetes\_Cytophagia\_Cytophagales\_Flammeovirgaceae\_Fulvivirga | -1.2232 | 0.3506 | -3.4892 | 0.0006 | TACGGAGGGTGCAAGCGTTGTCCGGATTTATTGGGTTTAAAGGGTGCGTAGGCGGTCGATTAAGTCAGTGGTGAAATCCTGCAGCTCAACTGTAGAACTGCCATTGATACTGGTTGACTTGAGTACAGACGAGGTAGGCGGAATTTATGATGTAGCGGTGAAATGCATAGATATCATAAAGAACACCGATAGCGAAGGCAGCTTACTAGACTGTAACTGACGCTGAGGCACGAAAGCGTGGGGAGCGAACAGG |
| Bacteria\_Bacteroidetes\_Sphingobacteriia\_Sphingobacteriales\_Saprospiraceae | -1.6369 | 0.3645 | -4.4907 | 0.0000 | TACGGAGGGTGCAAGCGTTATCCGGAATCACTGGGTTTAAAGGGTGCGTAGGCGGCAGTATAAGTCAGGTGTGAAAGCCTTCGGCTAAACCGGAGAATTGCATTTGATACTGTATTGCTTGAATTAGGCTGAGGTCAGCGGAATGTGTCATGTAGCGGTGAAATGCATAGATATGACATAGAACACCAATTGCGAAGGCAGCTGACTAGACCTTGATTGACGCTGAGGCACGAAAGCGTGGGGAGCGAACAGG |
| Bacteria\_Proteobacteria\_Alphaproteobacteria\_Rhodobacterales\_Rhodobacteraceae\_Celeribacter | -1.5380 | 0.3361 | -4.5755 | 0.0000 | TACGGAGGGGGTTAGCGTTGTTCGGAATTACTGGGCGTAAAGCGCACGTAGGCGGACTAGTCAGTCAGAGGTGAAATCCCGGGGCTCAACCCCGGAACTGCCTTTGATACTGCTAGTCTTGAGTTCGAGAGAGGTGAGTGGAATTCCGAGTGTAGAGGTGAAATTCGTAGATATTCGGAGGAACACCAGTGGCGAAGGCGGCTCACTGGCTCGATACTGACGCTGAGGTGCGAAAGTGTGGGGAGCAAACAGG |
| Bacteria\_Proteobacteria\_Alphaproteobacteria\_Rhizobiales\_Bradyrhizobiaceae\_Bradyrhizobium | -1.1081 | 0.2885 | -3.8410 | 0.0002 | TACGAAGGGGGCTAGCGTTGCTCGGAATCACTGGGCGTAAAGGGTGCGTAGGCGGGTCTTTAAGTCAGGGGTGAAATCCTGGAGCTCAACTCCAGAACTGCCTTTGATACTGAGGATCTTGAGTCCGGAAGAGGTGAGTGGAACTGCGAGTGTAGAGGTGAAATTCGTAGATATTCGCAAGAACACCAGTGGCGAAGGCGGCTCACTGGTCCGGTACTGACGCTGAGGCACGAAAGCGTGGGGAGCAAACAGG |
| Bacteria\_Cyanobacteria/Chloroplast\_Cyanobacteria\_Family\_XII\_GpXII | -1.8605 | 0.6405 | -2.9046 | 0.0041 | TACGGAGGAGGCAAGCGTTATCCGGAATGATTGGGCGTAAAGCGTCCGCAGGTGGCCATGTAAGTCTGCTGTCAAAACCCAGGGCTCAACTCTGGTCAGGCAGTGGAAACTACAAAGCTAGAGTCTGGTAGGGGCAAAGGGAATTCCCGGCGTAGCGGTGAAATGCGTAGATTTCGGGAAGAACATCGGTGGCGAAAGCGCTTTGCTAGACCAGAACTGACACTCAGGGACGAAAGCTAGGGGAGCGAATGGG |
| Bacteria\_Bacteroidetes\_Sphingobacteriia\_Sphingobacteriales\_Saprospiraceae | -1.9783 | 0.4953 | -3.9939 | 0.0001 | TACGGAGGGTGCAAGCGTTATCCGGAATCACTGGGTTTAAAGGGTGCGTAGGCGGTCCGACAAGTCAGAGGTGAAATGCACGAGCTTAACTGGTGAATTGCCTTTGAAACTGTCGAGCTAGAATCATGTTGAGGTCAGCGGAATGAGTCATGTAGCGGTGAAATGCATAGATATGACTTAGAACACCAATTGCGTAGGCAGCTGGCTAGGCATAGATTGACGCTGAGGCACGAAAGCGTGGGGAGCGAACAGG |
| Bacteria\_Proteobacteria\_Gammaproteobacteria\_Chromatiales\_Granulosicoccaceae\_Granulosicoccus | -1.3076 | 0.3980 | -3.2856 | 0.0012 | TACGGAGGGTGCAAGCGTTAATCGGAATTACTGGGCGTAAAGCGCGCGTAGGCGGCTTGGTCAGTCAGATGTGAAATCCCCGGGCTCAACCTGGGAACTGCATTTGATACTGCCAGGCTAGAGTATGTTAGAGGAATGCGGAATTCCAGGTGTAGCGGTGAAATGCGTAGATATCTGGAGGAACATCAGTGGCGAAGGCGGCATTCTGGAACAATACTGACGCTGAGGTGCGAAAGCGTGGGGAGCAAACAGG |
| Bacteria\_Actinobacteria\_Actinobacteria\_Acidimicrobiales\_Acidimicrobiaceae | -1.4463 | 0.4152 | -3.4833 | 0.0006 | CACGTAGGGGGCGAGCGTTGTCCGGATTTATTGGGCGTAAAGAGCTCGTAGGCTGTTCAGTAAGTCAGGTGTGAAAACCCAAGGCTCAACCTTGGGACGCCACTTGATACTGCTGTGACTAGAGTCCGGTAGAGGAGATTGGAATTCCTGGTGTAGCGGTGAAATGCGCAGATATCAGGAGGAACACCAACGGCGAAGGCAGATCTCTGGGCCGGTACTGACGCTGAGGAGCGAAAGCGTGGGGAGCGAACAGG |
| Bacteria\_Bacteroidetes\_Sphingobacteriia\_Sphingobacteriales\_Saprospiraceae\_Phaeodactylibacter | -1.1882 | 0.3388 | -3.5068 | 0.0006 | TACGGAGGGTGCAAGCGTTATCCGGAATCACTGGGTTTAAAGGGTGCGTAGGCGGCGTTATAAGTCAGAGGTGAAAGGCCACCGCTTAACGGTGGGACTGCCTTTGATACTGTAGTGCTTGAATAAGGTTGAGGTTAGCGGAATGAGTCATGTAGCGGTGAAATGCATAGATATGACTTAGAACACCAATTGCGAAGGCAGCTGGCTAGACCTTTATTGACGCTGAGGCACGAAAGCGTGGGGAGCGAACAGG |
| Bacteria\_Bacteroidetes\_Sphingobacteriia\_Sphingobacteriales\_Saprospiraceae\_Lewinella | -2.5308 | 0.6155 | -4.1115 | 0.0001 | TACGGAGGGTGCAAGCGTTATCCGGAATCACTGGGTTTAAAGGGTGCGTAGGCGGCCAGATAAGTCAGAAGTGAAAGTTTGCAGCTTAACTGTAAAATTGCTTTTGATACTGTTTGGCTTGAATTAGGTTGAGGTTAGCGGAATGTGACATGTAGCGGTGAAATGCATAGATATGTCATAGAACACCAATTGCGAAGGCAGCTGACTAGACCTATATTGACGCTGAGGCACGAAAGCGTGGGGAGCGAACAGG |
| Bacteria\_Bacteroidetes\_Flavobacteriia\_Flavobacteriales\_Flavobacteriaceae\_Marixanthomonas | -1.8134 | 0.3770 | -4.8101 | 0.0000 | TACGGAGGGTCCGAGCGTTATCCGGAATCATTGGGTTTAAAGGGTCCGTAGGCGGGCAGCTCAGTCAGTGGTGAAAGTTTGCGGCTTAACCGTAAAATTGCCATTGATACTGGTTGTCTTGAATTGGTGTGAAGTGGTTAGAATGAGTAGTGTAGCGGTGAAATGCATAGATATTACTCAGAATACCAATTGCGAAGGCAGATCACTAACACTATATTGACGCTGAGGGACGAAAGCGTGGGGAGCGAACAGG |
| Bacteria\_Proteobacteria\_Alphaproteobacteria\_Sphingomonadales\_Erythrobacteraceae\_Erythrobacter | -1.0266 | 0.3354 | -3.0605 | 0.0025 | TACGGAGGGAGCTAGCGTTGTTCGGAATTACTGGGCGTAAAGCGCGCGTAGGCGGCTTTTTAAGTCAGGGGTGAAATCCCGGAGCTCAACTCCGGAACTGCCCTTGAAACTGGATGGCTAGAATACTGGAGAGGTGAGTGGAATTCCGAGTGTAGAGGTGAAATTCGTAGATATTCGGAAGAACACCAGTGGCGAAGGCGACTCACTGGACAGTTATTGACGCTGAGGTGCGAAAGCGTGGGGAGCAAACAGG |
| Bacteria\_Planctomycetes\_Planctomycetia\_Planctomycetales\_Planctomycetaceae\_Blastopirellula | -1.6611 | 0.5086 | -3.2659 | 0.0013 | TACGAACTGTGCAAACGTTATTCGGAATCACTGGGCTTAAAGGGTGCGTAGGCGGATTTGAAGGTCTGATGTGAAAGCCCACGGCTCAACCGTGGAATTGCGTTGGAAACCACAAGTCTTGAGGAGATCAGAGGTGAGCGGAACTGATGGTGGAGCGGTGAAATGCGTTGATATCATCAGGAACACCGGTGGCGAAAGCGGCTCGCTGGGATCTTTCTGACGCTGAGGCACGAAAGCTAGGGGAGCGAACGGG |
| Bacteria\_Proteobacteria\_Gammaproteobacteria\_Alteromonadales\_Alteromonadales\_incertae\_sedis\_Teredinibacter | 1.4771 | 0.4901 | 3.0137 | 0.0029 | TACGGAGGGTGCGAGCGTTAATCGGAATTACTGGGCGTAAAGCGCGCGTAGGCGGCTGTCTAAGCTAGATGTGAAAGCCCCGGGCTCAACCTGGGAACTGCATTTAGAACTGGGCAGCTAGAGTACAGCAGAGGATAGTGGAATTTCAGGTGTAGCGGTGAAATGCGTAGAGATCTGAAGGAACATCAGTGGCGAAGGCGACTGTCTGGGCTGATACTGACGCTGAGGTGCGAAAGCGTGGGGATCAAACAGG |
| Bacteria\_Bacteroidetes\_Sphingobacteriia\_Sphingobacteriales\_Saprospiraceae | -1.4990 | 0.3161 | -4.7417 | 0.0000 | TACGGAGGGTGCAAGCGTTATCCGGAATCACTGGGTTTAAAGGGTGCGTAGGCGGATAAATAAGTCAGAGGTGAAAGCTCTGAGCTCAACTGAGGAATTGCCTTTGATACTGTTTATCTTGAATGATGTTGAGGTTAGCGGAATGAGTCATGTAGCGGTGAAATGCATAGATATGACTTAGAACACCAATTGCGAAGGCAGCTAGCTAGGCATTTATTGACGCTGAGGCACGAAAGCGTGGGGAGCGAACAGG |
| Bacteria\_Bacteroidetes\_Cytophagia\_Cytophagales | -1.8002 | 0.5589 | -3.2209 | 0.0015 | TACGGAGGGTGCAAGCGTTATCCGGATTTACTGGGTTTAAAGGGTGCGTAGGCGGCTCCTTAAGTCAGTGGTGAAAGCCTGGCGCTTAACGCCAGAACTGCCATTGATACTGGGGAGCTTGAGTCAAGAAGAGGTAAGCAGAATTCATGGTGTAGCGGTGAAATGCTTAGATACCATGAGGAATTCCAATAGCGAAGGCAGCTTACTGGTCTTGTACTGACGCTGAGGCACGAAAGCGTGGGGAGCGAACAGG |
| Bacteria\_Bacteroidetes\_Flavobacteriia\_Flavobacteriales\_Flavobacteriaceae\_Aquimarina | -1.6936 | 0.3810 | -4.4449 | 0.0000 | TACGGAGGATGCAAGCGTTATCCGGAATCATTGGGTTTAAAGGGTCCGTAGGCGGGTTTGTAAGTCAGTGGTGAAAGTTTGCGGCTCAACCGTAAAATTGCCATTGATACTGCAAGTCTTGAATCATTGTGAAGTGGTTAGAATATGTAGTGTAGCGGTGAAATGCATAGATATTACATAGAATACCGATTGCGAAGGCAGATCACTAACAATGTATTGACGCTGATGGACGAAAGCGTGGGTAGCGAACAGG |
| Bacteria\_Proteobacteria\_Betaproteobacteria\_Rhodocyclales\_Rhodocyclaceae\_Azonexus | -2.1132 | 0.4482 | -4.7147 | 0.0000 | TACGTAGGGTGCGAGCGTTAATCGGAATTACTGGGCGTAAAGCGTGCGCAGGCGGTTTTTTAAGATAGGCGTGAAATCCCCGGGCTTAACCTGGGAACTGCGCTTATGACTGGAAGACTAGAGTACGGCAGAGGGGGGTGGAATTCCACGTGTAGCAGTGAAATGCGTAGAGATGTGGAGGAACACCGATGGCGAAGGCAGCCCCCTGGGCCGATACTGACGCTCATGCACGAAAGCGTGGGGAGCAAACAGG |
| Bacteria\_Proteobacteria\_Alphaproteobacteria\_Caulobacterales\_Hyphomonadaceae | -1.3676 | 0.3736 | -3.6607 | 0.0003 | TACGGAGGGGGCTAGCGTTGTTCGGAATTACTGGGCGTAAAGCGTGCGTAGGCGGATTGGAAAGTTAGGTGTGAAATCCCGGAGCTCAACTCCGGAACTGCACTTAAAACTCCCAGTCTAGAGTTCTGGAGAGGTAAGTGGAATTCCTAGTGTAGAGGTGAAATTCATAGATATTAGGAGGAACACCAGAGGCGAAGGCGGCTTACTGGACAGACACTGACGCTGAGGCACGAAAGTGTGGGTAGCAAACAGG |
| Bacteria\_Bacteroidetes\_Sphingobacteriia\_Sphingobacteriales\_Saprospiraceae | -2.1952 | 0.4882 | -4.4967 | 0.0000 | TACGGAGGGTGCAAGCGTTATCCGGAATCACTGGGTTTAAAGGGTGCGTAGGCGGCGTTATAAGTCAGAGGTGAAATCCCACGGCTCAACTGTGGAACTGCCTTTGATACTGTAATGCTTGAATAAGGCTGAGGTTAGCGGAATGTGTCATGTAGCGGTGAAATGCATAGATATGACATAGAACACCAATTGCGAAGGCAGCTGACTAGACCTTTATTGACGCTGAGGCACGAAAGCGTGGGGAGCGAACAGG |
| Bacteria\_Bacteroidetes\_Flavobacteriia\_Flavobacteriales\_Flavobacteriaceae\_Winogradskyella | 2.0626 | 0.4738 | 4.3530 | 0.0000 | TACGGAGGATCCAAGCGTTATCCGGAATCATTGGGTTTAAAGGGTCCGTAGGTTGATAATTAAGTCAGAGGTGAAAGTTTGCGGCTCAACCGTAAAATTGCCTTTGATACTGGTTATCTTGAATCATTGTGAAGTGGTTAGAATATGTAGTGTAGCGGTGAAATGCATAGATATTACATAGAATACCAATTGCGAAGGCAGATCACTAACAATGTATTGACACTGATGGACGAAAGCGTGGGGAGCGAACGGG |
| Bacteria\_Proteobacteria\_Gammaproteobacteria\_Chromatiales\_Ectothiorhodospiraceae\_Thiogranum | 1.7960 | 0.6174 | 2.9088 | 0.0041 | TACGGAGGGTGCGAGCGTTAATCGGAATTACTGGGCGTAAAGCGCGCGTAGGCGGTTTGGTCAGTCGGATGTGAAAGCCCTGGGCTTAACCTGGGAACTGCATTCGATACTGCCTGACTAGAGTATGATAGAGGTAAGTGGAATTCCTGGTGTAGCGGTGAAATGCGTAGATATCAGGAGGAACATCAGTGGCGAAGGCGGCTTACTGGATCAATACTGACGCTGAGGTGCGAAAGCGTGGGGAGCAAACAGG |
| Bacteria\_Proteobacteria\_Alphaproteobacteria\_Caulobacterales\_Hyphomonadaceae | -1.7831 | 0.5589 | -3.1903 | 0.0017 | TACGGAGGGGGCTAGCGTTGTTCGGAATTACTGGGCGTAAAGCGTGCGTAGGCGGACTTTTAAGTTAGGGGTGAAATCCCGAGGCTCAACCTCGGAACTGCCCTTAAAACTGGAAGTCTTGAGTTCTGGAGAGGTAAGTGGAATTGCTAGTGTAGAGGTGAAATTCGTAGATATTAGCAGGAACACCAGAGGCGAAGGCGGCTTACTGGACAGATACTGACGCTGAGGCACGAAAGTGTGGGGAGCAAACAGG |
| Bacteria\_Bacteroidetes\_Sphingobacteriia\_Sphingobacteriales\_Saprospiraceae | -2.6811 | 0.5313 | -5.0466 | 0.0000 | TACGGAGGGTGCAAGCGTTATCCGGAATCACTGGGTTTAAAGGGTGCGTAGGCGGGTATATAAGTCAGAGGTGAAAGCTCACAGCTTAACTGTGGAATTGCCTTTGATACTGTATATCTTGAATAATGTTGAGGTTAGCGGAATGAGTCATGTAGCGGTGAAATGCATAGATATGACTTAGAACACCAATTGCGAAGGCAGCTAGCTAGGCATTCATTGACGCTGAGGCACGAAAGCGTGGGGAGCGAACAGG |
| Bacteria\_Proteobacteria\_Gammaproteobacteria\_Arenicellales\_Arenicellaceae\_Arenicella | -1.8608 | 0.6404 | -2.9055 | 0.0041 | TACGGAGGGTGCAAGCGTTAATCGGAATTACTGGGCGTAAAGCGCGCGTAGGTGGTTTGATAAGTCAGATGTGAAAGCCCCGGGCTTAACCTGGGAACTGCATTTGATACTGTCTTACTAGAGTATGGTAGAGGGAAGTGGAATTCCACATGTAGCGGTGAAATGCGTAGAGATGTGGAGGAACACCAGTGGCGAAGGCGACTTCCTGGACCAATACTGACACTGAGGTGCGAAAGCGTGGGGAGCAAACAGG |
| Bacteria\_Bacteroidetes\_Flavobacteriia\_Flavobacteriales\_Flavobacteriaceae\_Kordia | -1.2854 | 0.4213 | -3.0513 | 0.0026 | TACGGAGGATCCAAGCGTTATCCGGAATCATTGGGTTTAAAGGGTCCGCAGGCTGTTGTTTAAGTCAGAGGTGAAAGTTTGCAGCTCAACTGTAAAATTGCCTTTGATACTGGATGACTTGAGTAATAATGAAGTGGTTAGAATATGTAGTGTAGCGGTGAAATGCATAGATATTACATAGAATACCGATTGCGAAGGCAGATCACTAATTATATACTGACGCTGATGGACGAAAGCGTGGGGAGCGAACAGG |
| Bacteria\_Bacteroidetes\_Flavobacteriia\_Flavobacteriales\_Flavobacteriaceae\_Bizionia | 1.4598 | 0.4189 | 3.4849 | 0.0006 | TACGGAGGATCCAAGCGTTATCCGGAATCATTGGGTTTAAAGGGTCCGTAGGTGGATAATTAAGTCAGAGGTGAAAGTCTGCAGCTCAACTGTAGAATTGCCTTTGATACTGGTTATCTTGAGTTATTATGAAGTAGTTAGAATATGTAGTGTAGCGGTGAAATGCATAGATATTACATAGAATACCAATTGCGAAGGCAGATTACTAATAATATACTGACACTGATGGACGAAAGCGTGGGGAGCGAACAGG |
| Bacteria\_Bacteroidetes\_Sphingobacteriia\_Sphingobacteriales\_Saprospiraceae | -1.1999 | 0.3385 | -3.5446 | 0.0005 | TACGGAGGGTGCAAGCGTTATCCGGAATCACTGGGTTTAAAGGGTGCGTAGGCGGCTTAATAAGTCAGAGGTGAAAGTTTGCAGCTTAACTGTAAAATTGCCTTTGAAACTGTTGAGCTTGAATAGCGTTGAGGTCAGCGGAATGTGACATGTAGCGGTGAAATGCATAGATATGTCATAGAACACCAATTGCGAAGGCAGCTGGCTGGGCGTTTATTGACGCTGAGGCACGAAAGCGTGGGGAGCGAACAGG |
| Bacteria\_Proteobacteria\_Alphaproteobacteria\_Caulobacterales\_Hyphomonadaceae\_Fretibacter | -1.2878 | 0.4487 | -2.8698 | 0.0046 | TACGGAGGGGGCTAGCGTTGTTCGGAATTACTGGGCGTAAAGCGTGCGTAGGCGGATTATTAAGTTAGGTGTGAAATCCCGGGGCTCAACCCCGGAACTGCACTTAAAACTGGTAGTCTTGAGTTCTGGAGAGGTAAGTGGAATTCCTAGTGTAGAGGTGAAATTCGTAGATATTAGGAGGAACACCAGAGGCGAAGGCGGCTTACTGGACAGATACTGACGCTGAGGCACGAAAGTGTGGGGAGCAAACAGG |
| Bacteria\_Bacteroidetes\_Sphingobacteriia\_Sphingobacteriales\_Saprospiraceae | -1.3568 | 0.4451 | -3.0486 | 0.0026 | TACGGAGGGTGCAAGCGTTATCCGGAATCACTGGGTTTAAAGGGTGCGTAGGCGGATATATAAGTCAGAGGTGAAAGCTCACAGCTTAACTGTGGAACTGCCTTTGATACTGTATATCTTGAATTATGTTGAGGTTAGCGGAATGAGTCATGTAGCGGTGAAATGCATAGATATGACTTAGAACACCAATTGCGAAGGCAGCTAGCTAGGCATTGATTGACGCTGAGGCACGAAAGCGTGGGGAGCGAACAGG |
| Bacteria\_Bacteroidetes\_Cytophagia\_Cytophagales | -1.2922 | 0.3977 | -3.2492 | 0.0014 | TACGTAGGGTGCAAGCGTTGTCCGGATTTATTGGGTTTAAAGGGTGCGCAGGCGGCCTATTAAGTTAGTGGTGAAATCGTCCGGCTTAACCGGATACCCGCCATTGATACTGGTAGGCTTGAGTATGTCAGAGGAAGTTGGAATGCGTGGTGTAGCGGTGAAATGCATAGATATCACGCAGAACACCGATTGCGAAGGCAGATTTCTATGGCAAAACTGACGCTCAGGCACGAAAGCGTGGGTAGCGAACAGG |
| Bacteria\_Bacteroidetes\_Sphingobacteriia\_Sphingobacteriales\_Saprospiraceae\_Lewinella | -1.8600 | 0.5555 | -3.3484 | 0.0010 | TACGGAAGGTGCAAGCGTTATCCGGAATCACTGGGTTTAAAGGGTGCGTAGGCGGCCTAATAAGTCAGAAGTGAAAGCTCATCGCTTAACGATGGAATTGCTTTTGATACTGTTAGGCTTGAAACAGGTTGAGGTTAGCGGAATGTGGCATGTAGCGGTGAAATGCTTAGATATGCCATAGAACACCGATTGCGAAGGCAGCTGACTGGACCTTGTTTGACGCTGAGGCACGAAAGCGTGGGGAGCGAACAGG |
| Bacteria\_Actinobacteria\_Actinobacteria | -1.2655 | 0.4008 | -3.1571 | 0.0019 | CACGTAGGGATCGAGCGTTGTCCGGAATTATTGGGCGTAAAGAGCTCGTAGGTGGTTCAGTAAGTCGGATGTGAAAACTCGAGGCTCAACCTCGAGACGCCATCCGATACTGCTGTGACTAGAATTCGGTAGAGGAGTGTGGAATTCCTGGTGTAGCGGTGAAATGCGCAGATATCAGGAGGAACACCAACAGCGAAGGCAGCACTCTGGGCCGACATTGACACTGAGGAGCGAAAGCGTGGGGAGCAAACAGG |
| Bacteria\_Proteobacteria\_Alphaproteobacteria\_Sphingomonadales\_Erythrobacteraceae\_Altererythrobacter | -1.0581 | 0.3549 | -2.9812 | 0.0032 | TACGGAGGGAGCTAGCGTTGTTCGGAATTACTGGGCGTAAAGCGCGCGTAGGCGGTTTTTCAAGTCAGGGGTGAAATCCCGGAGCTCAACTCCGGAACTGCCCTTGAAACTGGATGACTAGAATCCTGGAGAGGCGAGTGGAATTCCGAGTGTAGAGGTGAAATTCGTAGATATTCGGAAGAACACCAGTGGCGAAGGCGACTCGCTGGACAGGTATTGACGCTGAGGTGCGAAAGCGTGGGGAGCAAACAGG |
| Bacteria\_Bacteroidetes\_Sphingobacteriia\_Sphingobacteriales\_Saprospiraceae | -1.7242 | 0.4625 | -3.7283 | 0.0003 | TACGGAGGGTGCAAGCGTTATCCGGAATCACTGGGTTTAAAGGGTGCGTAGGCGGCATTATAAGTCAGAAGTGAAAGCCCACCGCTTAACGGTGGAACTGCTTTTGATACTGTAAAGCTTGAATCAGGCTGAGGTCAGCGGAATGAGTCATGTAGCGGTGAAATGCATAGATATGACTTAGAACACCAATTGCGAAGGCAGCTGACTAGACCTGTATTGACGCTGAGGCACGAAAGCGTGGGGAGCGAACAGG |
| Bacteria\_Proteobacteria\_Gammaproteobacteria\_Chromatiales | -2.0811 | 0.6291 | -3.3084 | 0.0011 | TACTGAAGGTGCAAGCGTTAATCGGAATTACTGGGCGTAAAGCGCGCGTAGGCGGTTTATTAAGTCGGATGTGAAAGCCCTGGGCTCAACCTGGGAACTGCATTCGATACTGGTAAACTAGAGTATGTTAGAGGGAAGTGGAATTCCGGGTGTAGCGGTGAAATGCGTAGATATCCGGAGGAACACCAATGGCGAAGGCAACTTCCTGGGACAATACTGACGCTGAGGTGCGAAAGCGTGGGGAGCAAACGGG |
| Bacteria\_Cyanobacteria/Chloroplast\_Cyanobacteria\_Family\_IV\_GpIV | -2.3408 | 0.7558 | -3.0972 | 0.0023 | TACGGGGGATGCAAGCGTTATCCGGAATTATTGGGCGTAAAGCGTCCGTAGGCGGCTAGACAAGTCTGCTGTTAAAGCGTGCGGCTTAACCGCATACCGGCAGTGGAAACTGTTTAGCTAGAGTGTGGTAGGGGTAGAGGGAATTCCCGGTGTAGCGGTGAAATGCGTAGATATCGGGAAGAACACCAGTGGCGAAAGCGCTCTGCTGGGCCACAACTGACGCTGATGGACGAAAGCTAGGGGAGCGAAAGGG |
| Bacteria\_Proteobacteria\_Alphaproteobacteria\_Caulobacterales\_Hyphomonadaceae\_Ponticaulis | 1.3246 | 0.4212 | 3.1453 | 0.0019 | TACGAAGGGGGCTAGCGTTGTTCGGAATTACTGGGCGTAAAGCGCGCGTAGGCGGACCACCAAGTTGGATGTGAAATCCCGATGCTCAACATCGGAACTGCATTCAAAACTGTTGGTCTAGAGGATGGGAGAGGTCAGTGGAATACCGAGTGTAGAGGTGAAATTCGTAGATATTCGGTGGAACACCAGTGGCGAAGGCGACTGACTGGACCATTTCTGACGCTGAGGTGCGAAAGTGTGGGGAGCAAACAGG |
| Bacteria\_Proteobacteria\_Alphaproteobacteria\_Rhodobacterales\_Rhodobacteraceae | -1.6306 | 0.5668 | -2.8771 | 0.0045 | TACGGAGGGGACTAGCGTTGTTCGGAATTACTGGGCGTAAAGCGCGCGTAGGCGGACTATTAAGTCAGGGGTGAAATCCCGGGGCTCAACCCCGGAACTGCCTTTGATACTGGTAGTCTTGAGTTCGAGAGAGGTGAGTGGAATTCCGAGTGTAGAGGTGAAATTCGTAGATATTCGGAGGAACACCAGTGGCGAAGGCGGCTCACTGGCTCGATACTGACGCTGAGGTGCGAAAGCGTGGGGAGCAAACAGG |
| Bacteria\_Bacteroidetes\_Sphingobacteriia\_Sphingobacteriales\_Saprospiraceae | -1.8804 | 0.5525 | -3.4035 | 0.0008 | TACGGAGGGTGCAAGCGTTATCCGGAATCACTGGGTTTAAAGGGTGCGTAGGCGGCGCAATAAGTCAGAGGTGAAAGCTCACCGCTTAACGGTGGAACTGCCTTTGATACTGTTGTGCTAGAATTTGGTTGAGGTTAGCGGAATGAGTCATGTAGCGGTGAAATGCATAGATATGACTTAGAACACCAATTGCGAAGGCAGCTGGCTAGACCAATATTGACGCTGAGGCACGAAAGCGTGGGGAGCGAACAGG |
| Bacteria\_Proteobacteria\_Alphaproteobacteria\_Rhodobacterales\_Rhodobacteraceae\_Paracoccus | -1.4439 | 0.3902 | -3.7005 | 0.0003 | TACGGAGGGGGCTAGCGTTGTTCGGAATTACTGGGCGTAAAGCGCACGTAGGCGGACTGGAAAGTTGGGGGTGAAATCCCGGGGCTCAACCTCGGAACTGCCTCCAAAACTATCAGTCTGGAGTTCGAGAGAGGTGAGTGGAATACCGAGTGTAGAGGTGAAATTCGTAGATATTCGGTGGAACACCAGTGGCGAAGGCGGCTCACTGGCTCGATACTGACGCTGAGGTGCGAAAGCGTGGGGAGCAAACAGG |
| Bacteria\_Bacteroidetes\_Sphingobacteriia\_Sphingobacteriales\_Saprospiraceae\_Phaeodactylibacter | -1.7983 | 0.5588 | -3.2183 | 0.0015 | TACGGAGGGTGCAAGCGTTATCCGGAATCACTGGGTTTAAAGGGTGCGTAGGTGGCATTATAAGTCAGAGGTGAAAGCCTACCGCTTAACGGTAGAACTGCCTTTGATACTGTAGTGCTTGAATTAGGCTGAGGTCAGCGGAATGTGACATGTAGCGGTGAAATGCATAGATATGTCATAGAACACCAATTGCGAAGGCAGCTGGCTAGACCTATATTGACACTGAGGCACGAAAGCGTGGGGAGCGAACAGG |
| Bacteria\_Bacteroidetes\_Flavobacteriia\_Flavobacteriales\_Flavobacteriaceae\_Croceitalea | -1.1874 | 0.3830 | -3.1003 | 0.0022 | TACGGAGGGTCCGAGCGTTATCCGGAATCATTGGGTTTAAAGGGTCCGTAGGCGGGCGTATAAGTCAGGGGTGAAAGTTTGCGGCTCAACCGTAAAATTGCCTTTGATACTGTACGTCTTGAGTTATAGTGGAGTTGCCGGAACATGTGGTGTAGCGGTGAAATGCATAGATATCACATAGAACACCGATCGCGAAGGCAGGTGACTAACTATATACTGACGCTGATGGACGAAAGCGTGGGGAGCGAACGGG |
| Bacteria\_Bacteroidetes\_Flavobacteriia\_Flavobacteriales\_Cryomorphaceae | -1.4062 | 0.3733 | -3.7671 | 0.0002 | TACGGAGGGTCCAAGCGTTATCCGGATTTATTGGGTTTAAAGGGTTCGTAGGCGGATTTTTAAGTCAGTGGTGAAAGCCCACAGCTCAACTGTGGAACTGCCATTGATACTGGAAATCTTGAGTGTAGTTGAAGTTAGCGGAATGTGTCATGTAGCGGTGAAATGCTTAGATATGACCCAGAACACCGATTGCGAAAGCAGCTAACTAAGTTATTACTGACGCTGAGGAACGAAAGCGTGGGGAGCGAACAGG |
| Bacteria\_Proteobacteria\_Gammaproteobacteria\_Arenicellales\_Arenicellaceae\_Arenicella | -2.1773 | 0.7637 | -2.8511 | 0.0048 | TACGGAGGGTGCAAGCGTTAATCGGAATTACTGGGCGTAAAGCGCGCGTAGGTGGCTTATTAAGTCAGATGTGAAAGCCCCGGGCTTAACCTGGGAACTGCATCCGATACTGGTAAGCTAGAGTATGGTAGAGGGAAGTGGAATTCCACATGTAGCGGTGAAATGCGTAGAGATGTGGAGGAACACCAGTGGCGAAGGCGACTTCCTGGACCAATACTGACACTGAGGTGCGAAAGCGTGGGGAGCAAACAGG |
| Bacteria\_Bacteroidetes\_Sphingobacteriia\_Sphingobacteriales\_Chitinophagaceae\_Vibrionimonas | -2.8605 | 0.7384 | -3.8741 | 0.0001 | TACGGAGGGTGCAAGCGTTATCCGGATTCACTGGGTTTAAAGGGTGCGTAGGCGGGTAGGTAAGTCAGAGGTGAAATCCTGGAGCTTAACTCCAGAACTGCCTTTGATACTATCTATCTTGAATATGGTGGAGGTAAGCGGAATATGTCATGTAGCGGTGAAATGCATAGATATGACATAGAACACCTATTGCGAAGGCAGCTTACTACGCCTATATTGACGCTGAGGCACGAAAGCGTGGGGATCAAACAGG |
| Bacteria\_Bacteroidetes\_Flavobacteriia\_Flavobacteriales\_Cryomorphaceae\_Wandonia | -2.4241 | 0.7526 | -3.2209 | 0.0015 | TACGGAGGGTGCAAGCGTTATCCGGAATCATTGGGTTTAAAGGGTCCGCAGGCGGACGCATAAGTCAGTGGTGAAATCCCATCGCTCAACGATGGAACTGCCATTGATACTGTGTGTCTTGAATTCGGTCGAAGTAGGCGGAATGTGTCATGTAGCGGTGAAATGCATAGATATGACACAGAACACCGATAGCGAAGGCAGCTTACTAGGCCTGAATTGACGCTCAGGGACGAAAGCGTGGGTAGCGAACAGG |
| Bacteria\_Proteobacteria\_Alphaproteobacteria\_Rhodobacterales\_Rhodobacteraceae\_Loktanella | -1.2450 | 0.3977 | -3.1308 | 0.0020 | TACGGAGGGGGTTAGCGTTGTTCGGAATTACTGGGCGTAAAGCGCGCGTAGGCGGATTGGAAAGCTAGAGGTGAAATCCCGGGGCTCAACCCCGGAACTGCCTTTAGAACTATCAGTCTAGAGTTCGAGAGAGGTGAGTGGAATTCCGAGTGTAGAGGTGAAATTCGTAGATATTCGGAGGAACACCAGTGGCGAAGGCGGCTCACTGGCTCGATACTGACGCTGAGGTGCGAAAGTGTGGGGAGCAAACAGG |
| Bacteria\_Bacteroidetes\_Sphingobacteriia\_Sphingobacteriales\_Saprospiraceae\_Portibacter | -1.9641 | 0.6361 | -3.0878 | 0.0023 | TACGGAGGGTGCAAGCGTTATCCGGAATCACTGGGTTTAAAGGGTGCGTAGGCGGCGTTATAAGTCAGAAGTGAAAGCTCCCAGCTTAACTGGGAAATTGCTTTTGATACTGTAGTGCTTGAATCACGTTGAGGTTAGCGGAATGTGACATGTAGCGGTGAAATGCATAGATATGTCATAGAACACCAATTGCGTAGGCAGCTGACTAGGCGTGTATTGACGCTGAGGCACGAAAGCGTGGGGAGCGAACAGG |
| Bacteria\_Actinobacteria\_Actinobacteria\_Acidimicrobiales\_Acidimicrobiaceae\_Ilumatobacter | -1.7446 | 0.5021 | -3.4747 | 0.0006 | CACGTAGGCACCAAGCGTTATCCGGATTTATTGGGCGTAAAGAGCTCGTAGGCGGTTCGGTAAGTCGGGTGTGAAAATCCAAGGCTCAACCTTGGGACGCCACCCGATACTGCTGTGACTCGAGTGCAGTAGGGGAGCAGGGAATTCCTGGTGTAGCGGTGAAATGCGCAGATATCAGGAGGAACACCAGTGGCGAAGGCGCTGCTCTGGGCTGTAACTGACGCTGAGGAGCGAAAGCATGGGTAGCAAACAGG |
| Bacteria\_Proteobacteria\_Alphaproteobacteria\_Rhodobacterales\_Rhodobacteraceae | -2.4006 | 0.7525 | -3.1902 | 0.0017 | TACGGAGGGGACTAGCGTTGTTCGGAATTACTGGGCGTAAAGCGCACGTAGGCGGACCGAAAAGTTGGGGGTGAAATCCCAGGGCTCAACCCTGGAACTGCCTTCAAAACTGCCGGTCTAGAGTTCGAGAGAGGTGAGTGGAATTCCGAGTGTAGAGGTGAAATTCGTAGATATTCGGAGGAACACCAGTGGCGAAGGCGGCTCACTGGCTCGATACTGACGCTGAGGTGCGAAAGCGTGGGGAGCAAACAGG |
| Bacteria\_Bacteroidetes\_Sphingobacteriia\_Sphingobacteriales\_Saprospiraceae\_Portibacter | -2.2650 | 0.7593 | -2.9831 | 0.0032 | TACGGAGGGTGCAAGCGTTATCCGGAATCACTGGGTTTAAAGGGTGCGTAGGCGGCGCTATTAGTCAGAGGTGAAAGCCCACAGCTTAACTGTGGAATTGCCTTTGAAACTGTAGTGCTTGAATCACGTTGAGGTTAGCGGAATGTGACATGTAGCGGTGAAATGCTTAGATATGTCATAGAACACCAATTGCGTAGGCAGCTAACTAGGCGTGTATTGACGCTCAGGCACGAAAGCGTGGGTAGCGAACAGG |
| Bacteria\_Planctomycetes\_Planctomycetia\_Planctomycetales\_Planctomycetaceae\_Blastopirellula | 2.1422 | 0.7438 | 2.8799 | 0.0044 | GACGAACCGTCCGAACGTTATTCGGTATCACTGGGCTTAAAGAGTTCGTAGGCGGGCTAGCAGGTCAGGTGTGAAATCCCACAGCTCAACTGTGGAACTGCGCTTGAAACCGTTAGTCTTGAGGGAGATAGAGGTGAGCGGAACAGATGGTGGAGCGGTGAAATGCGTTGATATCATCTGGAACACCGGTGGCGAAAGCGGCTCACTGGATCTCTTCTGACGCTCAGGAACGAAAGCTAGGGTAGCGAACGGG |

### Genus-level

SI Table 7b. BBDML model estimates from all significant genera (from
corncob).

| taxon | estimate | std\_error | t\_value | p\_value |
| --- | --- | --- | --- | --- |
| Bacteria\_Proteobacteria\_Betaproteobacteria\_Burkholderiales\_Burkholderiaceae\_Burkholderia | 1.0842 | 0.2097 | 5.1696 | 0.0000 |
| Bacteria\_Bacteroidetes\_Sphingobacteriia\_Sphingobacteriales\_Sphingobacteriaceae\_Mucilaginibacter | -1.4392 | 0.1797 | -8.0095 | 0.0000 |
| Bacteria\_Proteobacteria\_Alphaproteobacteria\_Rhizobiales\_Rhizobiaceae\_Rhizobium | -1.3875 | 0.1863 | -7.4479 | 0.0000 |
| Bacteria\_Proteobacteria\_Alphaproteobacteria\_Sphingomonadales\_Sphingomonadaceae\_Sphingomonas | -1.4119 | 0.2020 | -6.9886 | 0.0000 |
| Bacteria\_Proteobacteria\_Gammaproteobacteria\_Pseudomonadales\_Moraxellaceae\_Acinetobacter | 0.7818 | 0.1486 | 5.2607 | 0.0000 |
| Bacteria\_Bacteroidetes\_Flavobacteriia\_Flavobacteriales\_Flavobacteriaceae\_Cloacibacterium | 0.6466 | 0.1374 | 4.7043 | 0.0000 |
| Bacteria\_Proteobacteria\_Alphaproteobacteria\_Rhodobacterales\_Rhodobacteraceae\_Aliiroseovarius | -0.5861 | 0.1396 | -4.2001 | 0.0000 |
| Bacteria\_Proteobacteria\_Gammaproteobacteria\_Arenicellales\_Arenicellaceae\_Arenicella | -0.4312 | 0.1544 | -2.7933 | 0.0058 |
| Bacteria\_Proteobacteria\_Alphaproteobacteria\_Caulobacterales\_Hyphomonadaceae\_Algimonas | -0.7125 | 0.1807 | -3.9432 | 0.0001 |
| Bacteria\_Bacteroidetes\_Flavobacteriia\_Flavobacteriales\_Flavobacteriaceae\_Elizabethkingia | 1.6772 | 0.2860 | 5.8638 | 0.0000 |
| Bacteria\_Bacteroidetes\_Flavobacteriia\_Flavobacteriales\_Flavobacteriaceae\_Dokdonia | -0.5686 | 0.1536 | -3.7009 | 0.0003 |
| Bacteria\_Proteobacteria\_Alphaproteobacteria\_Rhodobacterales\_Rhodobacteraceae\_Pseudoruegeria | -0.8814 | 0.2252 | -3.9144 | 0.0001 |
| Bacteria\_Proteobacteria\_Alphaproteobacteria\_Rhizobiales\_Bradyrhizobiaceae\_Bradyrhizobium | -1.3981 | 0.2260 | -6.1874 | 0.0000 |
| Bacteria\_Proteobacteria\_Alphaproteobacteria\_Rhodobacterales\_Rhodobacteraceae\_Thalassobius | -0.9062 | 0.2943 | -3.0797 | 0.0024 |
| Bacteria\_Proteobacteria\_Alphaproteobacteria\_Rhodobacterales\_Rhodobacteraceae\_Roseivivax | 0.6262 | 0.2252 | 2.7808 | 0.0060 |
| Bacteria\_Deinococcus-Thermus\_Deinococci\_Deinococcales\_Trueperaceae\_Truepera | -1.0073 | 0.2607 | -3.8636 | 0.0002 |
| Bacteria\_Bacteroidetes\_Flavobacteriia\_Flavobacteriales\_Flavobacteriaceae\_Aureitalea | -2.1718 | 0.6263 | -3.4679 | 0.0006 |
| Bacteria\_Proteobacteria\_Alphaproteobacteria\_Rhodospirillales\_Rhodospirillaceae\_Rhodospirillum | 3.1868 | 1.0179 | 3.1309 | 0.0020 |
| Bacteria\_Proteobacteria\_Gammaproteobacteria\_Oceanospirillales\_Hahellaceae\_Endozoicomonas | 0.9086 | 0.2691 | 3.3764 | 0.0009 |
| Bacteria\_Proteobacteria\_Epsilonproteobacteria\_Campylobacterales\_Campylobacteraceae\_Arcobacter | 1.0478 | 0.3434 | 3.0510 | 0.0026 |
| Bacteria\_Bacteroidetes\_Flavobacteriia\_Flavobacteriales\_Flavobacteriaceae\_Marixanthomonas | -1.7163 | 0.3619 | -4.7425 | 0.0000 |
| Bacteria\_Verrucomicrobia\_Opitutae\_Puniceicoccales\_Puniceicoccaceae\_Pelagicoccus | 0.9222 | 0.3358 | 2.7462 | 0.0066 |
| Bacteria\_Proteobacteria\_Betaproteobacteria\_Rhodocyclales\_Rhodocyclaceae\_Azonexus | -2.1218 | 0.4483 | -4.7335 | 0.0000 |
| Bacteria\_Proteobacteria\_Gammaproteobacteria\_Chromatiales\_Ectothiorhodospiraceae\_Thiogranum | 1.2670 | 0.4548 | 2.7858 | 0.0059 |
| Bacteria\_Bacteroidetes\_Flavobacteriia\_Flavobacteriales\_Flavobacteriaceae\_Bizionia | 1.2779 | 0.3967 | 3.2213 | 0.0015 |
| Bacteria\_Proteobacteria\_Alphaproteobacteria\_Caulobacterales\_Hyphomonadaceae\_Fretibacter | -0.6759 | 0.2453 | -2.7553 | 0.0064 |
| Bacteria\_Proteobacteria\_Alphaproteobacteria\_Rhodobacterales\_Rhodobacteraceae\_Paracoccus | -1.4557 | 0.3902 | -3.7308 | 0.0003 |
| Bacteria\_Bacteroidetes\_Flavobacteriia\_Flavobacteriales\_Flavobacteriaceae\_Croceitalea | -1.2329 | 0.3805 | -3.2400 | 0.0014 |
| Bacteria\_Bacteroidetes\_Sphingobacteriia\_Sphingobacteriales\_Chitinophagaceae\_Vibrionimonas | -2.8733 | 0.7384 | -3.8912 | 0.0001 |
| Bacteria\_Bacteroidetes\_Flavobacteriia\_Flavobacteriales\_Cryomorphaceae\_Wandonia | -2.4198 | 0.7527 | -3.2150 | 0.0015 |
| Bacteria\_Proteobacteria\_Alphaproteobacteria\_Rhizobiales\_Rhizobiales\_incertae\_sedis\_Bauldia | 1.7115 | 0.6216 | 2.7532 | 0.0065 |
| Bacteria\_Proteobacteria\_Alphaproteobacteria\_Caulobacterales\_Hyphomonadaceae\_Litorimonas | -1.7630 | 0.6454 | -2.7316 | 0.0069 |
| Bacteria\_Proteobacteria\_Alphaproteobacteria\_Rhizobiales\_Rhodobiaceae\_Anderseniella | 2.0006 | 0.7455 | 2.6836 | 0.0079 |

### Neutral Community Model

ASVs (identified here by their Genus assignment) that had significant
deviations (red dots) from the Sloan Neutral Community Model (error
bars).

### Relative abundance of differentially abundant taxa

*Note different x-axis scale for West and East facets*

---

# Appendix S5: Bacterial morphology

Bacterial genera traits were pulled from BacDive.

## Cell size

Table S5: Trait information for significant genera

| genus | respiration | motility | spore\_forming | gram\_stain | shape | dominant |
| --- | --- | --- | --- | --- | --- | --- |
| Azonexus | aerobic | Y | N | negative | rod | West |
| Bradyrhizobium | aerobic | Y | N | negative | rod | West |
| Elizabethkingia | aerobic | N | N | negative | rod | East |
| Marixanthomonas | aerobic | N | N | negative | rod | West |
| Mucilaginibacter | aerobic | N | NA | negative | rod | West |
| Rhizobium | aerobic | Y | N | negative | rod | West |
| Sphingomonas | aerobic | Y | N | negative | rod | West |

Distributions of cell dimensions and shapes for significant taxa were
compared to the overall distributions for all detected taxa.

Comparitive distributions of minimum cell dimensions

GLM model for minimum dimension distribution:

```
## min_dimension ~ signifigant_taxa
```

| term | estimate | std.error | statistic | p.value |
| --- | --- | --- | --- | --- |
| (Intercept) | 0.5995287 | 0.0100756 | 59.502782 | 0.0000000 |
| signifigant\_taxaTRUE | -0.0820287 | 0.0366264 | -2.239603 | 0.0253541 |

## Cell shape

Chi-square test results for significant taxa cell shapes:

```
## 
##  Pearson's Chi-squared test
## 
## data:  .
## X-squared = 7.5206, df = 10, p-value = 0.6755
```

There is no apparent enrichment in cell shape morphology in
significant taxa

## Cell morphology data

Data extracted from BacDive

| genus | length | width | shape | min\_length | min\_width | signifigant\_taxa | min\_dimension |
| --- | --- | --- | --- | --- | --- | --- | --- |
| Acanthopleuribacter | NA | NA | NA | NA | NA | FALSE | NA |
| Achromobacter | 1.4-2.8 µm | 0.4-1.1 µm | rod | 1.400 | 0.400 | FALSE | 0.400 |
| Achromobacter | 0.9-1.5 µm | 0.4-0.7 µm | rod | 0.900 | 0.400 | FALSE | 0.400 |
| Achromobacter | 0.9-1.5 µm | 0.4-0.7 µm | rod | 0.900 | 0.400 | FALSE | 0.400 |
| Achromobacter | 0.9-1.5 µm | 0.4-0.7 µm | rod | 0.900 | 0.400 | FALSE | 0.400 |
| Achromobacter | 0.9-2.0 µm | 0.4-0.7 µm | rod | 0.900 | 0.400 | FALSE | 0.400 |
| Achromobacter | 2.5-3.0 µm | 0.8-1.2 µm | coccus | 2.500 | 0.800 | FALSE | 0.800 |
| Acidovorax | 3 µm | 0.45 µm | rod | 3.000 | 0.450 | FALSE | 0.450 |
| Acidovorax | 1.5-2.0 µm | 0.3-0.5 µm | rod | 1.500 | 0.300 | FALSE | 0.300 |
| Acidovorax | 1.8 µm | 0.9 µm | rod | 1.800 | 0.900 | FALSE | 0.900 |
| Acinetobacter | 1.5 µm | 1.5 µm | ovoid | 1.500 | 1.500 | FALSE | 1.500 |
| Acinetobacter | 1.5 µm | 1.5 µm | ovoid | 1.500 | 1.500 | FALSE | 1.500 |
| Actibacter | NA | NA | NA | NA | NA | FALSE | NA |
| Aequorivita | 4.25 µm | 2 µm | rod | 4.250 | 2.000 | FALSE | 2.000 |
| Aequorivita | 0.5-20 µm | 0.4-0.5 µm | filament | 0.500 | 0.400 | FALSE | 0.400 |
| Aestuariibacter | NA | NA | NA | NA | NA | FALSE | NA |
| Aestuariispira | NA | NA | NA | NA | NA | FALSE | NA |
| Afipia | NA | NA | rod | NA | NA | FALSE | NA |
| Agarivorans | 1.7 µm | 0.8 µm | rod | 1.700 | 0.800 | FALSE | 0.800 |
| Agarivorans | 1.5-1.8 µm | 0.5-0.8 µm | rod | 1.500 | 0.500 | FALSE | 0.500 |
| Ahrensia | 1.5-3.5 µm | 0.6-0.8 µm | rod | 1.500 | 0.600 | FALSE | 0.600 |
| Albidovulum | NA | NA | NA | NA | NA | FALSE | NA |
| Albimonas | 3 µm | 1 µm | rod | 3.000 | 1.000 | FALSE | 1.000 |
| Alcanivorax | 0.5-1.5 µm | 0.3-0.6 µm | rod | 0.500 | 0.300 | FALSE | 0.300 |
| Alcanivorax | 2.4 µm | 0.5 µm | rod | 2.400 | 0.500 | FALSE | 0.500 |
| Alcanivorax | 1.1 µm | 0.55 µm | rod | 1.100 | 0.550 | FALSE | 0.550 |
| Alcanivorax | 2.05 µm | 0.65 µm | rod | 2.050 | 0.650 | FALSE | 0.650 |
| Alcanivorax | 0.8-2.0 µm | 0.3-0.7 µm | rod | 0.800 | 0.300 | FALSE | 0.300 |
| Algimonas | 2.13 µm | 0.38 µm | rod | 2.130 | 0.380 | FALSE | 0.380 |
| Algimonas | 2.13 µm | 0.37 µm | rod | 2.130 | 0.370 | FALSE | 0.370 |
| Algoriphagus | 0.7-1.9 µm | 0.2-0.5 µm | rod | 0.700 | 0.200 | FALSE | 0.200 |
| Algoriphagus | 1.65 µm | 0.5 µm | rod | 1.650 | 0.500 | FALSE | 0.500 |
| Algoriphagus | 2.8 µm | 0.3 µm | rod | 2.800 | 0.300 | FALSE | 0.300 |
| Algoriphagus | 1.4-2.0 µm | 0.4-0.7 µm | rod | 1.400 | 0.400 | FALSE | 0.400 |
| Algoriphagus | 0.8-1.0 µm | 0.4-0.6 µm | rod | 0.800 | 0.400 | FALSE | 0.400 |
| Algoriphagus | 1.7 µm | 0.65 µm | rod | 1.700 | 0.650 | FALSE | 0.650 |
| Algoriphagus | 2.5 µm | 0.5 µm | rod | 2.500 | 0.500 | FALSE | 0.500 |
| Algoriphagus | 1 µm | 0.3 µm | rod | 1.000 | 0.300 | FALSE | 0.300 |
| Algoriphagus | 1.5-15 µm | 0.5-2.5 µm | rod | 1.500 | 0.500 | FALSE | 0.500 |
| Algoriphagus | 6 µm | 0.6 µm | rod | 6.000 | 0.600 | FALSE | 0.600 |
| Algoriphagus | 6 µm | 0.6 µm | rod | 6.000 | 0.600 | FALSE | 0.600 |
| Algoriphagus | 2.3 µm | 0.35 µm | rod | 2.300 | 0.350 | FALSE | 0.350 |
| Algoriphagus | 6 µm | 0.6 µm | rod | 6.000 | 0.600 | FALSE | 0.600 |
| Algoriphagus | 2.5 µm | 0.5 µm | rod | 2.500 | 0.500 | FALSE | 0.500 |
| Aliiglaciecola | 2 µm | NA | rod | 2.000 | NA | FALSE | NA |
| Aliiroseovarius | 0.9-6.5 µm | 0.4-0.7 µm | ovoid | 0.900 | 0.400 | FALSE | 0.400 |
| Aliiroseovarius | 2.8 µm | 0.9 µm | rod | 2.800 | 0.900 | FALSE | 0.900 |
| Altererythrobacter | 0.98-1-22 µm | 0.45-0.65 µm | rod | 0.980 | 0.450 | FALSE | 0.450 |
| Altererythrobacter | 0.605 µm | 0.5 µm | rod | 0.605 | 0.500 | FALSE | 0.500 |
| Alteromonas | 1.2-1.7 µm | 0.3-0.6 µm | rod | 1.200 | 0.300 | FALSE | 0.300 |
| Alteromonas | 1.7-1.9 µm | 0.9-1.2 µm | rod | 1.700 | 0.900 | FALSE | 0.900 |
| Alteromonas | 1.5-2.5 µm | 0.3-0.8 µm | rod | 1.500 | 0.300 | FALSE | 0.300 |
| Alteromonas | 0.7-0.8 µm | 0.4-0.8 µm | rod | 0.700 | 0.400 | FALSE | 0.400 |
| Alteromonas | 1.2-2.5 µm | 0.5-0.9 µm | coccus | 1.200 | 0.500 | FALSE | 0.500 |
| Alteromonas | 1.2-2.5 µm | 0.75 µm | rod | 1.200 | 0.750 | FALSE | 0.750 |
| Alteromonas | 1.5 µm | 1 µm | rod | 1.500 | 1.000 | FALSE | 1.000 |
| Alteromonas | 3 µm | 0.7-1.2 µm | rod | 3.000 | 0.700 | FALSE | 0.700 |
| Alteromonas | 1.5-2.2 µm | 0.9 µm | rod | 1.500 | 0.900 | FALSE | 0.900 |
| Alteromonas | 1.8 µm | 0.4 µm | rod | 1.800 | 0.400 | FALSE | 0.400 |
| Amphritea | 0.8-1.5 µm | 0.4-0.6 µm | rod | 0.800 | 0.400 | FALSE | 0.400 |
| Amphritea | 1.3-2 µm | 0.6-0.9 µm | rod | 1.300 | 0.600 | FALSE | 0.600 |
| Amphritea | 0.9-1.7 µm | 0.6-0.9 µm | rod | 0.900 | 0.600 | FALSE | 0.600 |
| Amphritea | 1.25 µm | 0.5 µm | rod | 1.250 | 0.500 | FALSE | 0.500 |
| Anderseniella | 3 µm | 0.75 µm | rod | 3.000 | 0.750 | FALSE | 0.750 |
| Anoxybacillus | 2.5-2.7 µm | 0.8 µm | rod | 2.500 | 0.800 | FALSE | 0.800 |
| Anoxybacillus | 5 µm | 0.9 µm | rod | 5.000 | 0.900 | FALSE | 0.900 |
| Anoxybacillus | 1.4-4 µm | 0.3-0.6 µm | rod | 1.400 | 0.300 | FALSE | 0.300 |
| Anoxybacillus | 3.3 µm | 0.5 µm | rod | 3.300 | 0.500 | FALSE | 0.500 |
| Anoxybacillus | 4.3 µm | 1.05 µm | rod | 4.300 | 1.050 | FALSE | 1.050 |
| Anoxybacillus | 7 µm | 0.85 µm | rod | 7.000 | 0.850 | FALSE | 0.850 |
| Aquabacterium | NA | NA | rod | NA | NA | FALSE | NA |
| Aquihabitans | NA | NA | NA | NA | NA | FALSE | NA |
| Aquimarina | 2.5-4.0 µm | 0.4-0.6 µm | rod | 2.500 | 0.400 | FALSE | 0.400 |
| Aquimarina | 3.5-7.0 µm | 0.3-0.5 µm | rod | 3.500 | 0.300 | FALSE | 0.300 |
| Aquimarina | 34.5 µm | 0.3 µm | rod | 34.500 | 0.300 | FALSE | 0.300 |
| Aquimarina | 3-25.5 µm | 0.4-0.6 µm | rod | 3.000 | 0.400 | FALSE | 0.400 |
| Aquimarina | 1.0-2.0 µm | 0.2-0.4 µm | rod | 1.000 | 0.200 | FALSE | 0.200 |
| Aquimarina | 1.8-2 µm | 0.5-0.6 µm | rod | 1.800 | 0.500 | FALSE | 0.500 |
| Arcobacter | 1-2 µm | 0.5-0.8 µm | coccus | 1.000 | 0.500 | FALSE | 0.500 |
| Arcobacter | 0.8-2 µm | 0.4 µm | rod | 0.800 | 0.400 | FALSE | 0.400 |
| Arcobacter | 1.7 µm | 0.2 µm | rod | 1.700 | 0.200 | FALSE | 0.200 |
| Arcobacter | 1.1 µm | 0.5 µm | rod | 1.100 | 0.500 | FALSE | 0.500 |
| Arcobacter | 2.5 µm | 0.5 µm | rod | 2.500 | 0.500 | FALSE | 0.500 |
| Arcobacter | 2 µm | 0.4-0.5 µm | rod | 2.000 | 0.400 | FALSE | 0.400 |
| Arcobacter | 1.5-2.5 µm | 0.5 µm | rod | 1.500 | 0.500 | FALSE | 0.500 |
| Arcobacter | 1.5 µm | 0.2-0.4 µm | rod | 1.500 | 0.200 | FALSE | 0.200 |
| Arcobacter | 1.0-3.0 µm | 0.2-0.4 µm | rod | 1.000 | 0.200 | FALSE | 0.200 |
| Arenicella | 2.7 µm | 0.55 µm | rod | 2.700 | 0.550 | FALSE | 0.550 |
| Arenicella | 3.5 µm | 0.55 µm | rod | 3.500 | 0.550 | FALSE | 0.550 |
| Atopostipes | NA | NA | NA | NA | NA | FALSE | NA |
| Aureicoccus | 2 µm | 2 µm | coccus | 2.000 | 2.000 | FALSE | 2.000 |
| Aureispira | 4.5 µm | 0.75 µm | rod | 4.500 | 0.750 | FALSE | 0.750 |
| Aureitalea | NA | NA | NA | NA | NA | FALSE | NA |
| Azonexus | 1.65 µm | 0.4 µm | rod | 1.650 | 0.400 | TRUE | 0.400 |
| Bacteroides | 0.8-5 µm | 0.8-1 µm | rod | 0.800 | 0.800 | FALSE | 0.800 |
| Bacteroides | 0.4-20 µm | 0.4-0.8 µm | rod | 0.400 | 0.400 | FALSE | 0.400 |
| Bacteroides | 2.1 µm | 0.8 µm | rod | 2.100 | 0.800 | FALSE | 0.800 |
| Bacteroides | 8.5 µm | 1.2 µm | rod | 8.500 | 1.200 | FALSE | 1.200 |
| Bacteroides | 2.5 µm | 1.4 µm | rod | 2.500 | 1.400 | FALSE | 1.400 |
| Bacteroides | 1.4-55.0 µm | 1-1.9 µm | rod | 1.400 | 1.000 | FALSE | 1.000 |
| Bacteroides | 1-2.5 µm | 1-1.4 µm | rod | 1.000 | 1.000 | FALSE | 1.000 |
| Bacteroides | 0.7-2.9 µm | 0.5-1 µm | rod | 0.700 | 0.500 | FALSE | 0.500 |
| Bacteroides | 2.85 µm | 0.5 µm | rod | 2.850 | 0.500 | FALSE | 0.500 |
| Bacteroides | 1.7 µm | 0.75 µm | rod | 1.700 | 0.750 | FALSE | 0.750 |
| Bacteroides | 1.9 µm | 1 µm | rod | 1.900 | 1.000 | FALSE | 1.000 |
| Bacteroides | 1.5-4.5 µm | 0.8 µm | rod | 1.500 | 0.800 | FALSE | 0.800 |
| Bacteroides | 01-05 µm | 0.8 µm | rod | 1.000 | 0.800 | FALSE | 0.800 |
| Balneola | 2.6 µm | 0.7 µm | rod | 2.600 | 0.700 | FALSE | 0.700 |
| Balneola | 02-03 µm | 0.2 µm | rod | 2.000 | 0.200 | FALSE | 0.200 |
| Bauldia | NA | NA | NA | NA | NA | FALSE | NA |
| Bdellovibrio | NA | NA | NA | NA | NA | FALSE | NA |
| Bizionia | 2 µm | 0.4 µm | rod | 2.000 | 0.400 | FALSE | 0.400 |
| Bizionia | 3.25 µm | 0.45 µm | rod | 3.250 | 0.450 | FALSE | 0.450 |
| Bizionia | 2.5 µm | 0.45 µm | rod | 2.500 | 0.450 | FALSE | 0.450 |
| Bizionia | 2.5 µm | 0.4 µm | rod | 2.500 | 0.400 | FALSE | 0.400 |
| Bizionia | 02-03 µm | 0.2-0.4 µm | rod | 2.000 | 0.200 | FALSE | 0.200 |
| Blastopirellula | 1.1-1.5 µm | 0.6-0.8 µm | other | 1.100 | 0.600 | FALSE | 0.600 |
| Blautia | NA | NA | NA | NA | NA | FALSE | NA |
| Bradyrhizobium | 2-4 µm | 0.6 µm | rod | 2.000 | 0.600 | TRUE | 0.600 |
| Bradyrhizobium | 2-3 µm | 0.7 µm | rod | 2.000 | 0.700 | TRUE | 0.700 |
| Bradyrhizobium | 1.35 µm | 1.35 µm | rod | 1.350 | 1.350 | TRUE | 1.350 |
| Bradyrhizobium | 1.35 µm | 0.46 µm | rod | 1.350 | 0.460 | TRUE | 0.460 |
| Bradyrhizobium | 2-3 µm | 0.7 µm | rod | 2.000 | 0.700 | TRUE | 0.700 |
| Bradyrhizobium | 1.5-4.5 µm | 0.5 µm | rod | 1.500 | 0.500 | TRUE | 0.500 |
| Bradyrhizobium | 4.5 µm | 0.8 µm | rod | 4.500 | 0.800 | TRUE | 0.800 |
| Brevundimonas | 1-2 µm | 0.3-0.4 µm | rod | 1.000 | 0.300 | FALSE | 0.300 |
| Brevundimonas | 2.75 µm | 0.4 µm | rod | 2.750 | 0.400 | FALSE | 0.400 |
| Brevundimonas | 1.6 µm | 0.5 µm | rod | 1.600 | 0.500 | FALSE | 0.500 |
| Brevundimonas | 5.1-5.6 µm | 1.2-1.8 µm | rod | 5.100 | 1.200 | FALSE | 1.200 |
| Brevundimonas | 1.5-3.5 µm | 0.3-0.7 µm | rod | 1.500 | 0.300 | FALSE | 0.300 |
| Brevundimonas | 2.35 µm | 0.4 µm | rod | 2.350 | 0.400 | FALSE | 0.400 |
| Brevundimonas | 01-03 µm | 0.4-0.6 µm | rod | 1.000 | 0.400 | FALSE | 0.400 |
| Brevundimonas | 2.75 µm | 0.5 µm | rod | 2.750 | 0.500 | FALSE | 0.500 |
| Brumimicrobium | 1.5-2.5 µm | 0.3-0.4 µm | rod | 1.500 | 0.300 | FALSE | 0.300 |
| Brumimicrobium | 0.8-1.5 µm | 0.3-0.4 µm | rod | 0.800 | 0.300 | FALSE | 0.300 |
| Brumimicrobium | 1.25 µm | 0.4 µm | rod | 1.250 | 0.400 | FALSE | 0.400 |
| Burkholderia | 2 µm | NA | rod | 2.000 | NA | FALSE | NA |
| Caldithrix | 04-12 µm | 0.2-0.3 µm | rod | 4.000 | 0.200 | FALSE | 0.200 |
| Campylobacter | 2.5-5 µm | 0.5-1 µm | rod | 2.500 | 0.500 | FALSE | 0.500 |
| Campylobacter | 1.9-3.3 µm | 0.2-0.4 µm | rod | 1.900 | 0.200 | FALSE | 0.200 |
| Campylobacter | 0.5-1.8 µm | 0.25-0.5 µm | rod | 0.500 | 0.250 | FALSE | 0.250 |
| Campylobacter | 1.2-2.5 µm | 0.2-0.5 µm | rod | 1.200 | 0.200 | FALSE | 0.200 |
| Campylobacter | 2.0-5.0 µm | 0.5-0.8 µm | rod | 2.000 | 0.500 | FALSE | 0.500 |
| Candidatus\_Anammoxoglobus | NA | NA | NA | NA | NA | FALSE | NA |
| Candidatus\_Brocadia | NA | NA | NA | NA | NA | FALSE | NA |
| Caulobacter | 1.5-2.1 µm | 0.5-0.6 µm | rod | 1.500 | 0.500 | FALSE | 0.500 |
| Caulobacter | 1.8-3.2 µm | 1.1-1.3 µm | rod | 1.800 | 1.100 | FALSE | 1.100 |
| Caulobacter | 1.55 µm | 0.6 µm | rod | 1.550 | 0.600 | FALSE | 0.600 |
| Celeribacter | 0.8-0.9 µm | 0.4-0.5 µm | rod | 0.800 | 0.400 | FALSE | 0.400 |
| Celeribacter | 0.8 µm | 0.3 µm | rod | 0.800 | 0.300 | FALSE | 0.300 |
| Celeribacter | 0.8-1.8 µm | 0.4-0.9 µm | rod | 0.800 | 0.400 | FALSE | 0.400 |
| Celerinatantimonas | 3 µm | 0.75 µm | rod | 3.000 | 0.750 | FALSE | 0.750 |
| Cerasicoccus | 0.9 µm | 0.9 µm | coccus | 0.900 | 0.900 | FALSE | 0.900 |
| Cesiribacter | 1.5-3.0 µm | 0.4-0.5 µm | rod | 1.500 | 0.400 | FALSE | 0.400 |
| Chromohalobacter | 2.85 µm | 0.4 µm | rod | 2.850 | 0.400 | FALSE | 0.400 |
| Chromohalobacter | 2.3-4.2 µm | 0.35-0.5 µm | rod | 2.300 | 0.350 | FALSE | 0.350 |
| Chromohalobacter | 3.85 µm | 0.65 µm | rod | 3.850 | 0.650 | FALSE | 0.650 |
| Chryseobacterium | 2 µm | 0.65 µm | rod | 2.000 | 0.650 | FALSE | 0.650 |
| Chryseobacterium | 1.5 µm | 0.5 µm | rod | 1.500 | 0.500 | FALSE | 0.500 |
| Chryseobacterium | 2.4 µm | 0.8 µm | rod | 2.400 | 0.800 | FALSE | 0.800 |
| Chryseobacterium | 01-02 µm | 0.4-0.8 µm | rod | 1.000 | 0.400 | FALSE | 0.400 |
| Chryseobacterium | 3 µm | 0.75 µm | rod | 3.000 | 0.750 | FALSE | 0.750 |
| Chryseobacterium | 1.25 µm | 0.5 µm | rod | 1.250 | 0.500 | FALSE | 0.500 |
| Chryseobacterium | 1.2-1.8 µm | 0.6-0.7 µm | rod | 1.200 | 0.600 | FALSE | 0.600 |
| Chryseobacterium | 1.1-2.0 µm | 0.5-0.8 µm | rod | 1.100 | 0.500 | FALSE | 0.500 |
| Chryseobacterium | 1.6-2.0 µm | 0.6-0.8 µm | rod | 1.600 | 0.600 | FALSE | 0.600 |
| Chryseobacterium | 1.2 µm | 0.6 µm | rod | 1.200 | 0.600 | FALSE | 0.600 |
| Chryseobacterium | 1.45 µm | 0.4 µm | rod | 1.450 | 0.400 | FALSE | 0.400 |
| Chryseobacterium | 0.85 µm | 0.39 µm | rod | 0.850 | 0.390 | FALSE | 0.390 |
| Chryseobacterium | 2.75 µm | 0.45 µm | rod | 2.750 | 0.450 | FALSE | 0.450 |
| Chryseobacterium | 2 µm | 1 µm | rod | 2.000 | 1.000 | FALSE | 1.000 |
| Chryseobacterium | 1 µm | 0.5 µm | rod | 1.000 | 0.500 | FALSE | 0.500 |
| Chryseobacterium | 2 µm | 1 µm | rod | 2.000 | 1.000 | FALSE | 1.000 |
| Chryseobacterium | 2 µm | 1 µm | rod | 2.000 | 1.000 | FALSE | 1.000 |
| Chryseobacterium | 1.4-2.7 µm | 0.5-0.6 µm | rod | 1.400 | 0.500 | FALSE | 0.500 |
| Chryseobacterium | 0.9-4 µm | 0.5-1 µm | ovoid | 0.900 | 0.500 | FALSE | 0.500 |
| Chryseobacterium | 1.8 µm | 0.6 µm | rod | 1.800 | 0.600 | FALSE | 0.600 |
| Chryseobacterium | 2.05 µm | 0.55 µm | rod | 2.050 | 0.550 | FALSE | 0.550 |
| Chryseobacterium | 2.55 µm | 1 µm | rod | 2.550 | 1.000 | FALSE | 1.000 |
| Chryseobacterium | 2.25 µm | 0.7 µm | rod | 2.250 | 0.700 | FALSE | 0.700 |
| Chryseobacterium | 1.75 µm | 0.65 µm | rod | 1.750 | 0.650 | FALSE | 0.650 |
| Chryseobacterium | 1.3 µm | 0.8 µm | rod | 1.300 | 0.800 | FALSE | 0.800 |
| Chryseobacterium | 1.3 µm | 0.9 µm | rod | 1.300 | 0.900 | FALSE | 0.900 |
| Chryseobacterium | 3.6 µm | 1.1 µm | rod | 3.600 | 1.100 | FALSE | 1.100 |
| Chryseobacterium | 2 µm | 1.25 µm | rod | 2.000 | 1.250 | FALSE | 1.250 |
| Chryseobacterium | 1 µm | 0.5 µm | rod | 1.000 | 0.500 | FALSE | 0.500 |
| Chryseobacterium | 0.9 µm | 0.5 µm | rod | 0.900 | 0.500 | FALSE | 0.500 |
| Chryseobacterium | 1.7 µm | 0.7 µm | rod | 1.700 | 0.700 | FALSE | 0.700 |
| Chryseobacterium | 1.85 µm | 0.7 µm | rod | 1.850 | 0.700 | FALSE | 0.700 |
| Chryseobacterium | 1.9 µm | 0.75 µm | rod | 1.900 | 0.750 | FALSE | 0.750 |
| Chryseobacterium | 1.2-2 µm | 0.7-1.2 µm | rod | 1.200 | 0.700 | FALSE | 0.700 |
| Chryseobacterium | 2-3.5 µm | 0.7-0.8 µm | rod | 2.000 | 0.700 | FALSE | 0.700 |
| Chryseobacterium | 1.25 µm | 0.65 µm | rod | 1.250 | 0.650 | FALSE | 0.650 |
| Chryseobacterium | 1.65 µm | 0.8 µm | rod | 1.650 | 0.800 | FALSE | 0.800 |
| Chryseobacterium | 1-2.5 µm | 0.6-0.7 µm | rod | 1.000 | 0.600 | FALSE | 0.600 |
| Cloacibacterium | 01-02 µm | 0.2-0.3 µm | rod | 1.000 | 0.200 | FALSE | 0.200 |
| Clostridium\_sensu\_stricto | NA | NA | NA | NA | NA | FALSE | NA |
| Clostridium\_XlVa | NA | NA | NA | NA | NA | FALSE | NA |
| Cohaesibacter | 1-6 µm | 0.6-1 µm | rod | 1.000 | 0.600 | FALSE | 0.600 |
| Cohaesibacter | 1.8 µm | 0.45 µm | rod | 1.800 | 0.450 | FALSE | 0.450 |
| Cohaesibacter | 1.9 µm | 0.65 µm | rod | 1.900 | 0.650 | FALSE | 0.650 |
| Cohaesibacter | 2 µm | 0.3 µm | rod | 2.000 | 0.300 | FALSE | 0.300 |
| Comamonas | 2 µm | 1 µm | rod | 2.000 | 1.000 | FALSE | 1.000 |
| Comamonas | 0.6-0.7 µm | 0.3-0.7 µm | coccus | 0.600 | 0.300 | FALSE | 0.300 |
| Comamonas | 1.9-2.1 µm | 0.4-0.7 µm | rod | 1.900 | 0.400 | FALSE | 0.400 |
| Comamonas | 0.9-1.5 µm | 0.35 µm | rod | 0.900 | 0.350 | FALSE | 0.350 |
| Comamonas | 1.35 µm | 0.35 µm | rod | 1.350 | 0.350 | FALSE | 0.350 |
| Comamonas | 1.35 µm | 0.6-0.8 µm | rod | 1.350 | 0.600 | FALSE | 0.600 |
| Comamonas | 0.9-1.5 µm | 0.5 µm | rod | 0.900 | 0.500 | FALSE | 0.500 |
| Comamonas | 1.0-2.0 µm | 0.5-1.0 µm | rod | 1.000 | 0.500 | FALSE | 0.500 |
| Comamonas | 2.0-2.5 µm | 1.0-2.0 µm | rod | 2.000 | 1.000 | FALSE | 1.000 |
| Coraliomargarita | 0.4-0.7 µm | NA | coccus | 0.400 | NA | FALSE | NA |
| Corallibacter | NA | NA | NA | NA | NA | FALSE | NA |
| Corallomonas | 1 µm | 0.35 µm | rod | 1.000 | 0.350 | FALSE | 0.350 |
| Croceitalea | 1.4-3.1 µm | 0.4-0.6 µm | rod | 1.400 | 0.400 | FALSE | 0.400 |
| Croceitalea | 1-2.8 µm | 0.3-0.5 µm | rod | 1.000 | 0.300 | FALSE | 0.300 |
| Crocinitomix | 0.9-2.5 µm | 0.2-0.4 µm | rod | 0.900 | 0.200 | FALSE | 0.200 |
| Cryomorpha | NA | NA | NA | NA | NA | FALSE | NA |
| Curvibacter | NA | NA | NA | NA | NA | FALSE | NA |
| Dasania | NA | NA | NA | NA | NA | FALSE | NA |
| Dechloromonas | 1.7 µm | 0.5 µm | rod | 1.700 | 0.500 | FALSE | 0.500 |
| Desulfatibacillum | 1.2-4.5 µm | 0.68 µm | rod | 1.200 | 0.680 | FALSE | 0.680 |
| Desulfatibacillum | 2.0-5.5 µm | 0.6-2.2 µm | rod | 2.000 | 0.600 | FALSE | 0.600 |
| Desulfatitalea | 2.75 µm | 0.55 µm | rod | 2.750 | 0.550 | FALSE | 0.550 |
| Desulfobulbus | 1.6-2.2 µm | 0.9-1.0 µm | oval | 1.600 | 0.900 | FALSE | 0.900 |
| Desulfobulbus | 2.25 µm | 1.25 µm | rod | 2.250 | 1.250 | FALSE | 1.250 |
| Desulfopila | 1.0-2.0 µm | 0.3-0.5 µm | rod | 1.000 | 0.300 | FALSE | 0.300 |
| Desulfopila | 1.9-3.8 µm | 0.7-1.2 µm | rod | 1.900 | 0.700 | FALSE | 0.700 |
| Desulfosarcina | 1.0-1.5 µm | 0.5 µm | rod | 1.000 | 0.500 | FALSE | 0.500 |
| Desulfosarcina | 6.0-27.5 µm | 0.8 µm | rod | 6.000 | 0.800 | FALSE | 0.800 |
| Desulfospira | NA | NA | NA | NA | NA | FALSE | NA |
| Dokdonia | 2.0-3.2 µm | 0.5 µm | rod | 2.000 | 0.500 | FALSE | 0.500 |
| Dokdonia | 2.5-4 µm | 0.5-0.7 µm | rod | 2.500 | 0.500 | FALSE | 0.500 |
| Dokdonia | 2.5-4 µm | 0.5-0.7 µm | rod | 2.500 | 0.500 | FALSE | 0.500 |
| Dokdonia | 2.5-4 µm | 0.5-0.7 µm | rod | 2.500 | 0.500 | FALSE | 0.500 |
| Draconibacterium | NA | NA | NA | NA | NA | FALSE | NA |
| Dysgonomonas | NA | NA | NA | NA | NA | FALSE | NA |
| Ekhidna | NA | NA | rod | NA | NA | FALSE | NA |
| Elizabethkingia | NA | NA | NA | NA | NA | TRUE | NA |
| Endozoicomonas | 2-3 µm | 0.5-0.8 µm | rod | 2.000 | 0.500 | FALSE | 0.500 |
| Endozoicomonas | 2 µm | 0.7 µm | rod | 2.000 | 0.700 | FALSE | 0.700 |
| Endozoicomonas | 2 µm | 0.7 µm | rod | 2.000 | 0.700 | FALSE | 0.700 |
| Endozoicomonas | 2 µm | 0.6 µm | rod | 2.000 | 0.600 | FALSE | 0.600 |
| Endozoicomonas | 6.5 µm | 0.6 µm | rod | 6.500 | 0.600 | FALSE | 0.600 |
| Enhydrobacter | NA | NA | NA | NA | NA | FALSE | NA |
| Enterovibrio | 0.5-1.0 µm | 0.55 µm | rod | 0.500 | 0.550 | FALSE | 0.500 |
| Erythrobacter | 0.8-1.6 µm | 0.2-0.7 µm | rod | 0.800 | 0.200 | FALSE | 0.200 |
| Erythrobacter | 1.3-2.5 µm | 0.6-1.0 µm | rod | 1.300 | 0.600 | FALSE | 0.600 |
| Erythrobacter | 1.0-1.3 µm | 0.2-0.3 µm | rod | 1.000 | 0.200 | FALSE | 0.200 |
| Escherichia/Shigella | NA | NA | NA | NA | NA | FALSE | NA |
| Euzebyella | 1.2-3.4 µm | 0.3-0.4 µm | rod | 1.200 | 0.300 | FALSE | 0.300 |
| Fabibacter | NA | NA | NA | NA | NA | FALSE | NA |
| Faecalibacterium | NA | NA | NA | NA | NA | FALSE | NA |
| Fangia | 1.1 µm | 0.525 µm | rod | 1.100 | 0.525 | FALSE | 0.525 |
| Ferrimonas | 1.5 µm | 0.9 µm | ovoid | 1.500 | 0.900 | FALSE | 0.900 |
| Ferrimonas | 1.35 µm | 0.5 µm | rod | 1.350 | 0.500 | FALSE | 0.500 |
| Ferrimonas | 1.7 µm | 0.35 µm | rod | 1.700 | 0.350 | FALSE | 0.350 |
| Filomicrobium | 1.75 µm | 0.75 µm | rod | 1.750 | 0.750 | FALSE | 0.750 |
| Flavobacterium | 2.5 µm | 0.9 µm | rod | 2.500 | 0.900 | FALSE | 0.900 |
| Flavobacterium | 1.75 µm | 0.35 µm | rod | 1.750 | 0.350 | FALSE | 0.350 |
| Flavobacterium | 2.5 µm | 0.9 µm | rod | 2.500 | 0.900 | FALSE | 0.900 |
| Flavobacterium | 1.25 µm | 0.9 µm | rod | 1.250 | 0.900 | FALSE | 0.900 |
| Flavobacterium | 2.5 µm | 0.8 µm | rod | 2.500 | 0.800 | FALSE | 0.800 |
| Flavobacterium | 5.25 µm | 0.3 µm | rod | 5.250 | 0.300 | FALSE | 0.300 |
| Flavobacterium | 6.75 µm | 1 µm | rod | 6.750 | 1.000 | FALSE | 1.000 |
| Flavobacterium | 4 µm | 1.5 µm | rod | 4.000 | 1.500 | FALSE | 1.500 |
| Flavobacterium | 2 µm | 0.5 µm | rod | 2.000 | 0.500 | FALSE | 0.500 |
| Flavobacterium | 1.5-3 µm | 0.3-0.5 µm | rod | 1.500 | 0.300 | FALSE | 0.300 |
| Flavobacterium | 1.8 µm | 0.5 µm | rod | 1.800 | 0.500 | FALSE | 0.500 |
| Flavobacterium | 5.8-6.3 µm | 0.7-0.8 µm | rod | 5.800 | 0.700 | FALSE | 0.700 |
| Flavobacterium | 2.5 µm | 0.5 µm | rod | 2.500 | 0.500 | FALSE | 0.500 |
| Flavobacterium | 3.5 µm | 0.5 µm | rod | 3.500 | 0.500 | FALSE | 0.500 |
| Flavobacterium | 1.1-2.3 µm | 0.2-0.3 µm | rod | 1.100 | 0.200 | FALSE | 0.200 |
| Flavobacterium | 1.6-12.5 µm | 0.3-0.6 µm | rod | 1.600 | 0.300 | FALSE | 0.300 |
| Flavobacterium | 1.5-2.5 µm | 0.35-0.55 µm | rod | 1.500 | 0.350 | FALSE | 0.350 |
| Flavobacterium | 3-10 µm | 0.3-0.5 µm | rod | 3.000 | 0.300 | FALSE | 0.300 |
| Flavobacterium | 02-03 µm | 0.5 µm | rod | 2.000 | 0.500 | FALSE | 0.500 |
| Flavobacterium | 1.5-2.5 µm | 0.3 µm | rod | 1.500 | 0.300 | FALSE | 0.300 |
| Flavobacterium | 04-06 µm | 1.25 µm | rod | 4.000 | 1.250 | FALSE | 1.250 |
| Flavobacterium | 3.5 µm | 0.5 µm | rod | 3.500 | 0.500 | FALSE | 0.500 |
| Flavobacterium | 2 µm | 0.6 µm | rod | 2.000 | 0.600 | FALSE | 0.600 |
| Flavobacterium | 1.6 µm | 0.4-0.5 µm | rod | 1.600 | 0.400 | FALSE | 0.400 |
| Flavobacterium | 03-04 µm | 0.4-0.5 µm | rod | 3.000 | 0.400 | FALSE | 0.400 |
| Flavobacterium | 03-04 µm | 0.5-0.7 µm | rod | 3.000 | 0.500 | FALSE | 0.500 |
| Flavobacterium | 1-5 µm | 0.3-0.4 µm | rod | 1.000 | 0.300 | FALSE | 0.300 |
| Flavobacterium | 1-5 µm | 0.5-0.7 µm | rod | 1.000 | 0.500 | FALSE | 0.500 |
| Flavobacterium | 2.5-6 µm | 0.4-0.7 µm | rod | 2.500 | 0.400 | FALSE | 0.400 |
| Flexithrix | NA | NA | NA | NA | NA | FALSE | NA |
| Fluviicola | 1.8-2.5 µm | 0.3-0.6 µm | rod | 1.800 | 0.300 | FALSE | 0.300 |
| Fluviicola | 1.8-2.2 µm | 0.45-0.65 µm | rod | 1.800 | 0.450 | FALSE | 0.450 |
| Fluviicola | 1.6 µm | 0.45 µm | rod | 1.600 | 0.450 | FALSE | 0.450 |
| Francisella | NA | NA | NA | NA | NA | FALSE | NA |
| Fretibacter | 2.95 µm | 0.45 µm | rod | 2.950 | 0.450 | FALSE | 0.450 |
| Fulvivirga | 5.5 µm | 0.4 µm | rod | 5.500 | 0.400 | FALSE | 0.400 |
| Fusicatenibacter | 8.25 µm | 0.75 µm | rod | 8.250 | 0.750 | FALSE | 0.750 |
| Fusobacterium | NA | NA | NA | NA | NA | FALSE | NA |
| Gaetbulimicrobium | NA | NA | NA | NA | NA | FALSE | NA |
| Gangjinia | NA | NA | NA | NA | NA | FALSE | NA |
| Gemmiger | NA | NA | NA | NA | NA | FALSE | NA |
| Geobacillus | 3.2-4.5 µm | 0.3-0.8 µm | rod | 3.200 | 0.300 | FALSE | 0.300 |
| Geobacillus | 4.4-5.8 µm | 1.1-1.4 µm | rod | 4.400 | 1.100 | FALSE | 1.100 |
| Geobacillus | 4.7-8.0 µm | 0.9-1.3 µm | rod | 4.700 | 0.900 | FALSE | 0.900 |
| Geobacillus | 4.0-7.0 µm | 0.6-0.8 µm | rod | 4.000 | 0.600 | FALSE | 0.600 |
| Geobacillus | 2.0-6.0 µm | 0.7-1.5 µm | rod | 2.000 | 0.700 | FALSE | 0.700 |
| Geobacillus | 6.0-12.0 µm | 0.9-1.5 µm | rod | 6.000 | 0.900 | FALSE | 0.900 |
| Geobacillus | 2.0-3.5 µm | 0.6-1.0 µm | rod | 2.000 | 0.600 | FALSE | 0.600 |
| Geobacter | 2.75 µm | 0.8 µm | rod | 2.750 | 0.800 | FALSE | 0.800 |
| Gilvibacter | 3.0-5.0 µm | 0.5-0.7 µm | rod | 3.000 | 0.500 | FALSE | 0.500 |
| Gimesia | 1.2-1.8 µm | 0.5-0.9 µm | other | 1.200 | 0.500 | FALSE | 0.500 |
| Gimesia | 1.2-1.4 µm | 0.7-0.9 µm | other | 1.200 | 0.700 | FALSE | 0.700 |
| Gimesia | 1.2-1.6 µm | 0.6-0.8 µm | other | 1.200 | 0.600 | FALSE | 0.600 |
| Gimesia | 1.6-2.2 µm | 1.1-1.5 µm | other | 1.600 | 1.100 | FALSE | 1.100 |
| Gimesia | 1.1-1.5 µm | 0.9-1.1 µm | other | 1.100 | 0.900 | FALSE | 0.900 |
| Glaciecola | NA | NA | NA | NA | NA | FALSE | NA |
| Gracilimonas | 1.1-1.8 µm | 0.4-0.5 µm | rod | 1.100 | 0.400 | FALSE | 0.400 |
| Gracilimonas | 2.7 µm | 0.3 µm | rod | 2.700 | 0.300 | FALSE | 0.300 |
| Gracilimonas | 7.25 µm | 0.4 µm | rod | 7.250 | 0.400 | FALSE | 0.400 |
| Gracilimonas | 15.35 µm | 0.25 µm | rod | 15.350 | 0.250 | FALSE | 0.250 |
| Granulosicoccus | NA | 1 µm | coccus | NA | 1.000 | FALSE | NA |
| Grimontia | 1.8-2.8 µm | 0.5-0.7 µm | spiral | 1.800 | 0.500 | FALSE | 0.500 |
| Gynuella | 2.8-3.1 µm | 1.5-1.8 µm | rod | 2.800 | 1.500 | FALSE | 1.500 |
| Haematobacter | NA | NA | NA | NA | NA | FALSE | NA |
| Haliangium | NA | NA | NA | NA | NA | FALSE | NA |
| Haliea | 1.6 µm | 0.5 µm | rod | 1.600 | 0.500 | FALSE | 0.500 |
| Haliscomenobacter | NA | NA | NA | NA | NA | FALSE | NA |
| Halobacteriovorax | 0.5-1 µm | 0.3 µm | vibrio | 0.500 | 0.300 | FALSE | 0.300 |
| Halochromatium | 4 µm | 2.5 µm | rod | 4.000 | 2.500 | FALSE | 2.500 |
| Haloferula | NA | NA | NA | NA | NA | FALSE | NA |
| Halomonas | 1.6 µm | 1 µm | rod | 1.600 | 1.000 | FALSE | 1.000 |
| Halomonas | 3 µm | 1.3 µm | rod | 3.000 | 1.300 | FALSE | 1.300 |
| Halomonas | 1.5-6 µm | 0.5-0.8 µm | rod | 1.500 | 0.500 | FALSE | 0.500 |
| Halomonas | 2.5 µm | 1 µm | rod | 2.500 | 1.000 | FALSE | 1.000 |
| Halomonas | 2.5 µm | 0.95 µm | rod | 2.500 | 0.950 | FALSE | 0.950 |
| Halomonas | 1.4 µm | 0.5 µm | rod | 1.400 | 0.500 | FALSE | 0.500 |
| Halomonas | 1.85 µm | 0.45 µm | rod | 1.850 | 0.450 | FALSE | 0.450 |
| Halomonas | 2.35 µm | 0.8 µm | rod | 2.350 | 0.800 | FALSE | 0.800 |
| Halomonas | 1.2 µm | 0.7 µm | rod | 1.200 | 0.700 | FALSE | 0.700 |
| Halomonas | 0.8-1.1 µm | 0.5-0.6 µm | rod | 0.800 | 0.500 | FALSE | 0.500 |
| Halomonas | 1.3-1.7 µm | 0.8-1 µm | rod | 1.300 | 0.800 | FALSE | 0.800 |
| Halomonas | 4 µm | 0.75 µm | rod | 4.000 | 0.750 | FALSE | 0.750 |
| Halomonas | 3 µm | 0.75 µm | rod | 3.000 | 0.750 | FALSE | 0.750 |
| Halomonas | 3 µm | 1 µm | coccus | 3.000 | 1.000 | FALSE | 1.000 |
| Halomonas | 3 µm | 1.3 µm | rod | 3.000 | 1.300 | FALSE | 1.300 |
| Halomonas | 1.0-1.6 µm | 0.6-0.8 µm | rod | 1.000 | 0.600 | FALSE | 0.600 |
| Halomonas | 1.2 µm | 0.35 µm | rod | 1.200 | 0.350 | FALSE | 0.350 |
| Halomonas | 1.8-2.4 µm | 0.6-0.8 µm | rod | 1.800 | 0.600 | FALSE | 0.600 |
| Halomonas | 1.6-2 µm | 0.5-0.7 µm | rod | 1.600 | 0.500 | FALSE | 0.500 |
| Halomonas | 1.2-1.6 µm | 0.6-0.8 µm | rod | 1.200 | 0.600 | FALSE | 0.600 |
| Halomonas | 2.25 µm | 0.75 µm | rod | 2.250 | 0.750 | FALSE | 0.750 |
| Halomonas | 2.5 µm | 1.1 µm | rod | 2.500 | 1.100 | FALSE | 1.100 |
| Halomonas | 3.25 µm | 0.86 µm | rod | 3.250 | 0.860 | FALSE | 0.860 |
| Halomonas | 1.3 µm | 0.75 µm | rod | 1.300 | 0.750 | FALSE | 0.750 |
| Halomonas | 2.5 µm | 1.5 µm | rod | 2.500 | 1.500 | FALSE | 1.500 |
| Halomonas | 2.5 µm | 1 µm | rod | 2.500 | 1.000 | FALSE | 1.000 |
| Halomonas | 2.5 µm | 1 µm | rod | 2.500 | 1.000 | FALSE | 1.000 |
| Hanstruepera | NA | NA | NA | NA | NA | FALSE | NA |
| Hellea | 2.7-5.6 µm | 0.28-0.48 µm | rod | 2.700 | 0.280 | FALSE | 0.280 |
| Hirschia | 2.45 µm | 0.6 µm | coccus | 2.450 | 0.600 | FALSE | 0.600 |
| Hirschia | 1.2-1.3 µm | 0.6-1.1 µm | rod | 1.200 | 0.600 | FALSE | 0.600 |
| Hirschia | 0.5-6 µm | 0.5-1 µm | rod | 0.500 | 0.500 | FALSE | 0.500 |
| Hoeflea | 0.7-2 µm | 0.3-0.5 µm | rod | 0.700 | 0.300 | FALSE | 0.300 |
| Hoeflea | 2.5 µm | 0.8 µm | rod | 2.500 | 0.800 | FALSE | 0.800 |
| Hydrogenophilus | 2.6 µm | 0.4 µm | rod | 2.600 | 0.400 | FALSE | 0.400 |
| Hyphomicrobium | 1.75 µm | 0.65 µm | rod | 1.750 | 0.650 | FALSE | 0.650 |
| Hyphomonas | NA | NA | NA | NA | NA | FALSE | NA |
| Iamia | 1.45 µm | 0.4 µm | rod | 1.450 | 0.400 | FALSE | 0.400 |
| Idiomarina | 0.8-1.4 µm | 0.2 µm | rod | 0.800 | 0.200 | FALSE | 0.200 |
| Idiomarina | 4.75 µm | 0.45 µm | rod | 4.750 | 0.450 | FALSE | 0.450 |
| Idiomarina | 1.0-2.4 µm | 0.3-0.4 µm | vibrio | 1.000 | 0.300 | FALSE | 0.300 |
| Idiomarina | 2.5 µm | 0.75 µm | rod | 2.500 | 0.750 | FALSE | 0.750 |
| Idiomarina | 3.5 µm | 0.75 µm | rod | 3.500 | 0.750 | FALSE | 0.750 |
| Ignavibacterium | 8.75 µm | 0.25 µm | rod | 8.750 | 0.250 | FALSE | 0.250 |
| Ilumatobacter | 1.4 µm | 0.5 µm | rod | 1.400 | 0.500 | FALSE | 0.500 |
| Ilumatobacter | 0.85 µm | 0.55 µm | rod | 0.850 | 0.550 | FALSE | 0.550 |
| Ilyobacter | NA | NA | NA | NA | NA | FALSE | NA |
| Jannaschia | 1.0-2.0 µm | 0.6-0.9 µm | rod | 1.000 | 0.600 | FALSE | 0.600 |
| Jannaschia | 1.75 µm | 0.65 µm | rod | 1.750 | 0.650 | FALSE | 0.650 |
| Jannaschia | 1.1-2.3 µm | 0.7-1.2 µm | rod | 1.100 | 0.700 | FALSE | 0.700 |
| Jannaschia | 2.25 µm | 0.6 µm | ovoid | 2.250 | 0.600 | FALSE | 0.600 |
| Jannaschia | 2 µm | 1 µm | rod | 2.000 | 1.000 | FALSE | 1.000 |
| Jannaschia | 1.75 µm | 0.85 µm | rod | 1.750 | 0.850 | FALSE | 0.850 |
| Jannaschia | 1.5 µm | 0.5 µm | rod | 1.500 | 0.500 | FALSE | 0.500 |
| Jannaschia | 1.5 µm | 0.5 µm | rod | 1.500 | 0.500 | FALSE | 0.500 |
| Kangiella | 1.75 µm | 0.75 µm | rod | 1.750 | 0.750 | FALSE | 0.750 |
| Kangiella | 0.95 µm | 0.4 µm | rod | 0.950 | 0.400 | FALSE | 0.400 |
| Kangiella | 2.5-3.5 µm | 0.75 µm | rod | 2.500 | 0.750 | FALSE | 0.750 |
| Kofleria | NA | NA | NA | NA | NA | FALSE | NA |
| Kordia | 2.45 µm | 0.75 µm | rod | 2.450 | 0.750 | FALSE | 0.750 |
| Kordiimonas | 1.0-2.9 µm | 0.4-0.7 µm | rod | 1.000 | 0.400 | FALSE | 0.400 |
| Kordiimonas | 1.8-3 µm | 0.7-0.9 µm | rod | 1.800 | 0.700 | FALSE | 0.700 |
| Kordiimonas | 3 µm | 0.45 µm | rod | 3.000 | 0.450 | FALSE | 0.450 |
| Kordiimonas | 0.95 µm | 0.75 µm | rod | 0.950 | 0.750 | FALSE | 0.750 |
| Kordiimonas | 4.5 µm | 0.65 µm | rod | 4.500 | 0.650 | FALSE | 0.650 |
| Kordiimonas | 1.35 µm | 0.25 µm | rod | 1.350 | 0.250 | FALSE | 0.250 |
| Lactobacillus | 6.25 µm | 0.5 µm | rod | 6.250 | 0.500 | FALSE | 0.500 |
| Lactobacillus | 20 µm | 0.75 µm | rod | 20.000 | 0.750 | FALSE | 0.750 |
| Lactobacillus | 11 µm | 0.75 µm | rod | 11.000 | 0.750 | FALSE | 0.750 |
| Lactobacillus | 16 µm | 1 µm | rod | 16.000 | 1.000 | FALSE | 1.000 |
| Lactobacillus | 5.75 µm | 1 µm | rod | 5.750 | 1.000 | FALSE | 1.000 |
| Leeuwenhoekiella | 1.4-4.1 µm | 0.4-0.7 µm | rod | 1.400 | 0.400 | FALSE | 0.400 |
| Leeuwenhoekiella | 1.5-4 µm | 0.4-0.7 µm | rod | 1.500 | 0.400 | FALSE | 0.400 |
| Leeuwenhoekiella | 2.3 µm | 0.45 µm | rod | 2.300 | 0.450 | FALSE | 0.450 |
| Legionella | 0.87 µm | 0.61 µm | rod | 0.870 | 0.610 | FALSE | 0.610 |
| Legionella | 1.5 µm | 0.54 µm | rod | 1.500 | 0.540 | FALSE | 0.540 |
| Legionella | 2 µm | 0.66 µm | rod | 2.000 | 0.660 | FALSE | 0.660 |
| Legionella | 1.55 µm | 0.7 µm | rod | 1.550 | 0.700 | FALSE | 0.700 |
| Leisingera | 1.8 µm | 0.9 µm | rod | 1.800 | 0.900 | FALSE | 0.900 |
| Leisingera | 1.4 µm | 1 µm | ovoid | 1.400 | 1.000 | FALSE | 1.000 |
| Lentilitoribacter | NA | NA | NA | NA | NA | FALSE | NA |
| Lewinella | 2-3.1 µm | 0.6-0.8 µm | rod | 2.000 | 0.600 | FALSE | 0.600 |
| Lewinella | 2-3.2 µm | 0.8-1 µm | rod | 2.000 | 0.800 | FALSE | 0.800 |
| Lewinella | 11.8 µm | 1.15 µm | rod | 11.800 | 1.150 | FALSE | 1.150 |
| Lewinella | 0.8 µm | 0.4 µm | rod | 0.800 | 0.400 | FALSE | 0.400 |
| Lewinella | 0.8 µm | 0.45 µm | rod | 0.800 | 0.450 | FALSE | 0.450 |
| Lewinella | 1.2-2 µm | 0.3-0.5 µm | rod | 1.200 | 0.300 | FALSE | 0.300 |
| Lishizhenia | 2.5 µm | 0.35 µm | rod | 2.500 | 0.350 | FALSE | 0.350 |
| Litoreibacter | 1.3 µm | 0.6 µm | coccus | 1.300 | 0.600 | FALSE | 0.600 |
| Litoreibacter | 1.5 µm | 0.8 µm | ovoid | 1.500 | 0.800 | FALSE | 0.800 |
| Litoreibacter | 1.9 µm | 0.5 µm | rod | 1.900 | 0.500 | FALSE | 0.500 |
| Litoreibacter | 1.9 µm | 0.5 µm | rod | 1.900 | 0.500 | FALSE | 0.500 |
| Litoribrevibacter | 1.5-2.5 µm | 0.4-0.6 µm | rod | 1.500 | 0.400 | FALSE | 0.400 |
| Litorilinea | 100 µm | 0.5 µm | rod | 100.000 | 0.500 | FALSE | 0.500 |
| Litorimonas | 1.5-1.9 µm | 0.6-0.8 µm | rod | 1.500 | 0.600 | FALSE | 0.600 |
| Loktanella | 1.9 µm | 0.8 µm | rod | 1.900 | 0.800 | FALSE | 0.800 |
| Loktanella | 2.5 µm | 0.75 µm | rod | 2.500 | 0.750 | FALSE | 0.750 |
| Loktanella | 3.5 µm | 1 µm | rod | 3.500 | 1.000 | FALSE | 1.000 |
| Luteolibacter | 0.9-1.5 µm | 0.2-0.5 µm | rod | 0.900 | 0.200 | FALSE | 0.200 |
| Luteolibacter | 1.35 µm | 0.65 µm | rod | 1.350 | 0.650 | FALSE | 0.650 |
| Lutibacter | 1-5.7 µm | 0.3-0.8 µm | rod | 1.000 | 0.300 | FALSE | 0.300 |
| Lutibacter | 0.5-10.0 µm | 0.2-0.4 µm | rod | 0.500 | 0.200 | FALSE | 0.200 |
| Lutibacter | 2-6 µm | 0.5 µm | rod | 2.000 | 0.500 | FALSE | 0.500 |
| Lutibacter | 1.2 µm | 0.3 µm | rod | 1.200 | 0.300 | FALSE | 0.300 |
| Lutibacter | 2.6 µm | 0.85 µm | rod | 2.600 | 0.850 | FALSE | 0.850 |
| Lutimaribacter | 2.2-2.5 µm | 0.5-0.8 µm | rod | 2.200 | 0.500 | FALSE | 0.500 |
| Lutimaribacter | 1.1 µm | 0.65 µm | rod | 1.100 | 0.650 | FALSE | 0.650 |
| Lutimonas | 2 µm | 0.85 µm | rod | 2.000 | 0.850 | FALSE | 0.850 |
| Magnetovibrio | 2 µm | 0.3 µm | spiral | 2.000 | 0.300 | FALSE | 0.300 |
| Malonomonas | NA | NA | NA | NA | NA | FALSE | NA |
| Maricaulis | NA | NA | NA | NA | NA | FALSE | NA |
| Maricurvus | NA | NA | NA | NA | NA | FALSE | NA |
| Marinobacter | 1.3-2.2 µm | 0.6-1.2 µm | rod | 1.300 | 0.600 | FALSE | 0.600 |
| Marinobacter | 1.5-2 µm | 0.5-1 µm | rod | 1.500 | 0.500 | FALSE | 0.500 |
| Marinobacter | 2.0-2.5 µm | 0.3-0.5 µm | rod | 2.000 | 0.300 | FALSE | 0.300 |
| Marinobacter | 1.3-2.6 µm | 0.4 µm | rod | 1.300 | 0.400 | FALSE | 0.400 |
| Marinobacter | 1-2.5 µm | 0.5 µm | rod | 1.000 | 0.500 | FALSE | 0.500 |
| Marinobacter | 1.75 µm | 0.35 µm | rod | 1.750 | 0.350 | FALSE | 0.350 |
| Marinobacter | 3 µm | 0.6 µm | rod | 3.000 | 0.600 | FALSE | 0.600 |
| Marinobacter | 2.7 µm | 0.4 µm | rod | 2.700 | 0.400 | FALSE | 0.400 |
| Marinobacter | 1.05 µm | 0.65 µm | ovoid | 1.050 | 0.650 | FALSE | 0.650 |
| Marinobacter | 1-2.5 µm | 0.4-0.8 µm | rod | 1.000 | 0.400 | FALSE | 0.400 |
| Marinobacter | 1.5-3 µm | 0.5-0.8 µm | rod | 1.500 | 0.500 | FALSE | 0.500 |
| Marinobacter | 1.9 µm | 0.65 µm | rod | 1.900 | 0.650 | FALSE | 0.650 |
| Marinobacter | 2.25 µm | 0.7 µm | rod | 2.250 | 0.700 | FALSE | 0.700 |
| Marinobacter | 1.1 µm | 0.45 µm | rod | 1.100 | 0.450 | FALSE | 0.450 |
| Marinobacter | 2.15 µm | 0.75 µm | rod | 2.150 | 0.750 | FALSE | 0.750 |
| Marinobacter | 1.5-2.8 µm | 0.5-0.7 µm | rod | 1.500 | 0.500 | FALSE | 0.500 |
| Marinobacter | 1.7-2.4 µm | 0.6-0.8 µm | rod | 1.700 | 0.600 | FALSE | 0.600 |
| Marinobacter | 1.6-3.0 µm | 0.3-0.5 µm | rod | 1.600 | 0.300 | FALSE | 0.300 |
| Marinobacter | 1.2-4.5 µm | 0.5-1.3 µm | rod | 1.200 | 0.500 | FALSE | 0.500 |
| Marinobacter | 3 µm | 1 µm | rod | 3.000 | 1.000 | FALSE | 1.000 |
| Marinobacter | 1.5 µm | 0.4 µm | rod | 1.500 | 0.400 | FALSE | 0.400 |
| Marinobacter | 6.6 µm | 1.85 µm | rod | 6.600 | 1.850 | FALSE | 1.850 |
| Marinobacter | 1.5-3.0 µm | 0.6-0.8 µm | rod | 1.500 | 0.600 | FALSE | 0.600 |
| Marinobacter | 1.6-2.5 µm | 0.45-0.55 µm | rod | 1.600 | 0.450 | FALSE | 0.450 |
| Marinobacter | 1.5-3.0 µm | 0.6-0.9 µm | rod | 1.500 | 0.600 | FALSE | 0.600 |
| Marinobacter | 1.0-1.3 µm | 0.4-0.5 µm | rod | 1.000 | 0.400 | FALSE | 0.400 |
| Marinobacter | 1.8-2.5 µm | 0.3-0.4 µm | rod | 1.800 | 0.300 | FALSE | 0.300 |
| Marinobacter | 2.5-3.5 µm | 0.3-0.5 µm | rod | 2.500 | 0.300 | FALSE | 0.300 |
| Marinobacter | 2-3 µm | 0.3-0.6 µm | rod | 2.000 | 0.300 | FALSE | 0.300 |
| Marinobacterium | 1.1-1.3 µm | 0.6-0.7 µm | rod | 1.100 | 0.600 | FALSE | 0.600 |
| Marinobacterium | 1.9-2.1 µm | 0.6-0.8 µm | rod | 1.900 | 0.600 | FALSE | 0.600 |
| Marinobacterium | 1.9-2.4 µm | 0.7-1 µm | rod | 1.900 | 0.700 | FALSE | 0.700 |
| Marinobacterium | 0.7-8 µm | 0.4-0.8 µm | rod | 0.700 | 0.400 | FALSE | 0.400 |
| Marinobacterium | 1.0-2.0 µm | 0.4-0.6 µm | rod | 1.000 | 0.400 | FALSE | 0.400 |
| Marinobacterium | 3.5 µm | 1 µm | rod | 3.500 | 1.000 | FALSE | 1.000 |
| Marinobacterium | 0.85 µm | 0.55 µm | rod | 0.850 | 0.550 | FALSE | 0.550 |
| Marinobacterium | 1.5 µm | 0.4 µm | rod | 1.500 | 0.400 | FALSE | 0.400 |
| Marinobacterium | 2 µm | 0.65 µm | rod | 2.000 | 0.650 | FALSE | 0.650 |
| Marinobacterium | 1.3 µm | 0.7 µm | rod | 1.300 | 0.700 | FALSE | 0.700 |
| Marinobacterium | 0.7 µm | 0.35 µm | rod | 0.700 | 0.350 | FALSE | 0.350 |
| Marinobacterium | 1.6-2.3 µm | 0.5-0.7 µm | rod | 1.600 | 0.500 | FALSE | 0.500 |
| Marinomonas | 3.5-4.0 µm | 0.8-1.0 µm | rod | 3.500 | 0.800 | FALSE | 0.800 |
| Marinomonas | 1.0-1.2 µm | 0.2-0.4 µm | rod | 1.000 | 0.200 | FALSE | 0.200 |
| Marinomonas | 1.2-3.9 µm | 0.5-0.9 µm | rod | 1.200 | 0.500 | FALSE | 0.500 |
| Marinomonas | 1.2-2.6 µm | 0.3-0.4 µm | rod | 1.200 | 0.300 | FALSE | 0.300 |
| Marinomonas | 2 µm | 0.5 µm | rod | 2.000 | 0.500 | FALSE | 0.500 |
| Marinomonas | 1.2 µm | 0.6-0.8 µm | rod | 1.200 | 0.600 | FALSE | 0.600 |
| Marinoscillum | 0.35 µm | NA | rod | 0.350 | NA | FALSE | NA |
| Maritimimonas | NA | NA | NA | NA | NA | FALSE | NA |
| Marivita | 2.25 µm | 0.6 µm | rod | 2.250 | 0.600 | FALSE | 0.600 |
| Marivita | 2.7 µm | 0.8 µm | rod | 2.700 | 0.800 | FALSE | 0.800 |
| Marixanthomonas | 2.5 µm | 0.45 µm | rod | 2.500 | 0.450 | TRUE | 0.450 |
| Mesoflavibacter | NA | NA | NA | NA | NA | FALSE | NA |
| Mesonia | 0.8-1.8 µm | 0.5-0.7 µm | rod | 0.800 | 0.500 | FALSE | 0.500 |
| Mesonia | 1-2.1 µm | 0.4-0.5 µm | rod | 1.000 | 0.400 | FALSE | 0.400 |
| Mesonia | 1.6-2.3 µm | 0.4-0.5 µm | rod | 1.600 | 0.400 | FALSE | 0.400 |
| Mesorhizobium | 0.5-1.2 µm | 0.4-0.5 µm | rod | 0.500 | 0.400 | FALSE | 0.400 |
| Mesorhizobium | 0.8-1.5 µm | 0.4-0.5 µm | rod | 0.800 | 0.400 | FALSE | 0.400 |
| Mesorhizobium | 1.4 µm | 0.6 µm | rod | 1.400 | 0.600 | FALSE | 0.600 |
| Mesorhizobium | 0.9-2.0 µm | 0.4-0.6 µm | rod | 0.900 | 0.400 | FALSE | 0.400 |
| Mesorhizobium | 1.13 µm | 0.38 µm | rod | 1.130 | 0.380 | FALSE | 0.380 |
| Mesorhizobium | 2 µm | 1 µm | rod | 2.000 | 1.000 | FALSE | 1.000 |
| Mesorhizobium | 1.5-2.5 µm | 0.6-0.7 µm | rod | 1.500 | 0.600 | FALSE | 0.600 |
| Mesorhizobium | 1.5-3.0 µm | 0.5-0.7 µm | rod | 1.500 | 0.500 | FALSE | 0.500 |
| Mesorhizobium | 1.5-2.0 µm | 1 µm | rod | 1.500 | 1.000 | FALSE | 1.000 |
| Mesorhizobium | 2 µm | 1 µm | rod | 2.000 | 1.000 | FALSE | 1.000 |
| Mesorhizobium | 1.5-3.0 µm | 0.3-0.5 µm | rod | 1.500 | 0.300 | FALSE | 0.300 |
| Mesorhizobium | 02-03 µm | 0.5 µm | rod | 2.000 | 0.500 | FALSE | 0.500 |
| Mesorhizobium | 1 µm | 0.5 µm | rod | 1.000 | 0.500 | FALSE | 0.500 |
| Mesorhizobium | 01-03 µm | 0.3-0.6 µm | rod | 1.000 | 0.300 | FALSE | 0.300 |
| Mesorhizobium | 01-03 µm | 0.3-0.6 µm | rod | 1.000 | 0.300 | FALSE | 0.300 |
| Mesorhizobium | 1.4 µm | 0.6 µm | rod | 1.400 | 0.600 | FALSE | 0.600 |
| Mesorhizobium | 1.5-2.0 µm | 0.3-0.6 µm | rod | 1.500 | 0.300 | FALSE | 0.300 |
| Mesorhizobium | 2.59 µm | 0.34 µm | rod | 2.590 | 0.340 | FALSE | 0.340 |
| Mesorhizobium | 2.61 µm | 0.33 µm | rod | 2.610 | 0.330 | FALSE | 0.330 |
| Mesorhizobium | 2 µm | 0.45 µm | rod | 2.000 | 0.450 | FALSE | 0.450 |
| Mesorhizobium | 1.435 µm | 0.45 µm | rod | 1.435 | 0.450 | FALSE | 0.450 |
| Mesorhizobium | 2.7 µm | 0.3 µm | rod | 2.700 | 0.300 | FALSE | 0.300 |
| Mesorhizobium | 1.65 µm | 0.53 µm | rod | 1.650 | 0.530 | FALSE | 0.530 |
| Mesorhizobium | 3.1 µm | 1.5 µm | rod | 3.100 | 1.500 | FALSE | 1.500 |
| Mesorhizobium | 1-2.5 µm | 0.3-0.6 µm | rod | 1.000 | 0.300 | FALSE | 0.300 |
| Mesorhizobium | 2 µm | 0.45 µm | rod | 2.000 | 0.450 | FALSE | 0.450 |
| Mesorhizobium | 01-03 µm | 0.3-0.5 µm | rod | 1.000 | 0.300 | FALSE | 0.300 |
| Mesorhizobium | 1.2-1.5 µm | 0.2-0.4 µm | rod | 1.200 | 0.200 | FALSE | 0.200 |
| Methylobacterium | 1.25 µm | 0.9 µm | rod | 1.250 | 0.900 | FALSE | 0.900 |
| Methylobacterium | 2.5 µm | 1.1 µm | rod | 2.500 | 1.100 | FALSE | 1.100 |
| Methylobacterium | 2.5 µm | 1.2 µm | rod | 2.500 | 1.200 | FALSE | 1.200 |
| Methylobacterium | 4.1 µm | 1.3 µm | rod | 4.100 | 1.300 | FALSE | 1.300 |
| Methylobacterium | 1.8 µm | 1.3 µm | rod | 1.800 | 1.300 | FALSE | 1.300 |
| Methylobacterium | 1.99 µm | 0.9 µm | rod | 1.990 | 0.900 | FALSE | 0.900 |
| Methylobacterium | 1.6-5.4 µm | 0.8-1.1 µm | rod | 1.600 | 0.800 | FALSE | 0.800 |
| Methylobacterium | 2.9 µm | 1.15 µm | rod | 2.900 | 1.150 | FALSE | 1.150 |
| Methylobacterium | 2.25 µm | 0.64 µm | rod | 2.250 | 0.640 | FALSE | 0.640 |
| Methylobacterium | 3.3 µm | 1.25 µm | ovoid | 3.300 | 1.250 | FALSE | 1.250 |
| Methylobacterium | 3.6 µm | 1.4 µm | ovoid | 3.600 | 1.400 | FALSE | 1.400 |
| Methylobacterium | 3.65 µm | 1.4 µm | ovoid | 3.650 | 1.400 | FALSE | 1.400 |
| Methylobacterium | 3.55 µm | 1.7 µm | rod | 3.550 | 1.700 | FALSE | 1.700 |
| Methylobacterium | 3.4 µm | 1.1 µm | rod | 3.400 | 1.100 | FALSE | 1.100 |
| Methylobacterium | 2 µm | 1 µm | rod | 2.000 | 1.000 | FALSE | 1.000 |
| Methylobacterium | 2.25 µm | 1 µm | rod | 2.250 | 1.000 | FALSE | 1.000 |
| Methylobacterium | 2.425 µm | 0.7 µm | rod | 2.425 | 0.700 | FALSE | 0.700 |
| Methylobacterium | 1.5-5 µm | 1-1.2 µm | rod | 1.500 | 1.000 | FALSE | 1.000 |
| Methylobacterium | 3.5 µm | 1.15 µm | rod | 3.500 | 1.150 | FALSE | 1.150 |
| Methylobacterium | 4 µm | 1.25 µm | rod | 4.000 | 1.250 | FALSE | 1.250 |
| Methylobacterium | 6.25 µm | 1.6 µm | rod | 6.250 | 1.600 | FALSE | 1.600 |
| Methylobacterium | 2.25 µm | 1.25 µm | rod | 2.250 | 1.250 | FALSE | 1.250 |
| Methyloligella | NA | NA | NA | NA | NA | FALSE | NA |
| Methylophaga | 1 µm | 0.3 µm | rod | 1.000 | 0.300 | FALSE | 0.300 |
| Methylophaga | 1.5 µm | 0.6 µm | rod | 1.500 | 0.600 | FALSE | 0.600 |
| Methylophaga | 1.5 µm | 0.7 µm | rod | 1.500 | 0.700 | FALSE | 0.700 |
| Methylophaga | 1.6 µm | 0.2 µm | rod | 1.600 | 0.200 | FALSE | 0.200 |
| Methylotenera | 0.8-1.2 µm | 0.3-0.7 µm | ovoid | 0.800 | 0.300 | FALSE | 0.300 |
| Microscilla | 10-100 µm | NA | rod | 10.000 | NA | FALSE | NA |
| Modicisalibacter | 2.5 µm | 0.8 µm | rod | 2.500 | 0.800 | FALSE | 0.800 |
| Mucilaginibacter | 1.7-2.1 µm | 0.4-0.6 µm | rod | 1.700 | 0.400 | TRUE | 0.400 |
| Mucilaginibacter | 0.7-1.5 µm | 0.3-0.6 µm | rod | 0.700 | 0.300 | TRUE | 0.300 |
| Mucilaginibacter | 0.7-1.4 µm | 0.2-0.5 µm | rod | 0.700 | 0.200 | TRUE | 0.200 |
| Mucilaginibacter | 0.6-0.7 µm | 0.4-0.5 µm | rod | 0.600 | 0.400 | TRUE | 0.400 |
| Mucilaginibacter | 1-1.5 µm | 0.2-0.5 µm | rod | 1.000 | 0.200 | TRUE | 0.200 |
| Mucilaginibacter | 2.7-3.2 µm | 0.5-0.8 µm | rod | 2.700 | 0.500 | TRUE | 0.500 |
| Mucilaginibacter | 1.8-3.2 µm | 0.6-0.9 µm | pleomorphic | 1.800 | 0.600 | TRUE | 0.600 |
| Mucilaginibacter | 1.5-2.8 µm | 0.4-0.8 µm | rod | 1.500 | 0.400 | TRUE | 0.400 |
| Mucilaginibacter | 1.9-2.9 µm | 0.82-0.84 µm | rod | 1.900 | 0.820 | TRUE | 0.820 |
| Mucilaginibacter | 0.8-3.5 µm | 0.3-0.7 µm | rod | 0.800 | 0.300 | TRUE | 0.300 |
| Mucilaginibacter | 1.6 µm | 0.46 µm | rod | 1.600 | 0.460 | TRUE | 0.460 |
| Mucilaginibacter | 2.5 µm | 0.5 µm | rod | 2.500 | 0.500 | TRUE | 0.500 |
| Mucilaginibacter | 1.305 µm | 0.385 µm | rod | 1.305 | 0.385 | TRUE | 0.385 |
| Mucilaginibacter | 1.75 µm | 0.5 µm | rod | 1.750 | 0.500 | TRUE | 0.500 |
| Mucilaginibacter | 1.5 µm | 0.6 µm | rod | 1.500 | 0.600 | TRUE | 0.600 |
| Mucilaginibacter | 1.1-1.5 µm | 0.6-0.8 µm | rod | 1.100 | 0.600 | TRUE | 0.600 |
| Mucilaginibacter | 01-04 µm | 0.3-0.5 µm | rod | 1.000 | 0.300 | TRUE | 0.300 |
| Mucilaginibacter | 01-02 µm | 0.4-0.6 µm | rod | 1.000 | 0.400 | TRUE | 0.400 |
| Mucilaginibacter | 01-03 µm | 0.3-0.5 µm | rod | 1.000 | 0.300 | TRUE | 0.300 |
| Mucilaginibacter | 2 µm | 0.6 µm | rod | 2.000 | 0.600 | TRUE | 0.600 |
| Mucilaginibacter | 0.8-1.2 µm | 0.2-0.4 µm | rod | 0.800 | 0.200 | TRUE | 0.200 |
| Mucilaginibacter | 1.1-1.5 µm | 0.3-1.1 µm | rod | 1.100 | 0.300 | TRUE | 0.300 |
| Mucilaginibacter | 1.1-1.3 µm | 0.3-0.4 µm | rod | 1.100 | 0.300 | TRUE | 0.300 |
| Mucilaginibacter | 1.25-2.3 µm | 0.65-0.75 µm | rod | 1.250 | 0.650 | TRUE | 0.650 |
| Mucilaginibacter | 2.15 µm | 0.65 µm | rod | 2.150 | 0.650 | TRUE | 0.650 |
| Mucilaginibacter | 20.75 µm | 0.45 µm | rod | 20.750 | 0.450 | TRUE | 0.450 |
| Muricauda | 1.4-3.3 µm | 0.2-0.3 µm | rod | 1.400 | 0.200 | FALSE | 0.200 |
| Muricauda | 1.7-2.5 µm | 0.4-0.8 µm | rod | 1.700 | 0.400 | FALSE | 0.400 |
| Muricauda | 1.1-3.0 µm | 0.5-0.8 µm | rod | 1.100 | 0.500 | FALSE | 0.500 |
| Muricauda | 0.7-1 µm | 0.3-0.5 µm | rod | 0.700 | 0.300 | FALSE | 0.300 |
| Muricauda | 1.1-5.3 µm | 0.2-0.6 µm | rod | 1.100 | 0.200 | FALSE | 0.200 |
| Muricauda | 1.8-6.0 µm | 0.2-0.4 µm | rod | 1.800 | 0.200 | FALSE | 0.200 |
| Muricauda | 1.7 µm | 0.28 µm | rod | 1.700 | 0.280 | FALSE | 0.280 |
| Muricauda | 5.25 µm | 0.3 µm | rod | 5.250 | 0.300 | FALSE | 0.300 |
| Muricauda | 4.25 µm | 0.35 µm | rod | 4.250 | 0.350 | FALSE | 0.350 |
| Muricauda | 4.25 µm | 0.3 µm | rod | 4.250 | 0.300 | FALSE | 0.300 |
| Muricauda | 11.5 µm | 0.4 µm | rod | 11.500 | 0.400 | FALSE | 0.400 |
| Muricauda | 3 µm | 0.35 µm | rod | 3.000 | 0.350 | FALSE | 0.350 |
| Muricauda | 1.5 µm | 1.5 µm | rod | 1.500 | 1.500 | FALSE | 1.500 |
| Muricauda | 2.7 µm | 0.475 µm | rod | 2.700 | 0.475 | FALSE | 0.475 |
| Muricauda | 2.25 µm | 0.4 µm | rod | 2.250 | 0.400 | FALSE | 0.400 |
| Muricauda | 1.1-2.7 µm | 0.3-0.6 µm | rod | 1.100 | 0.300 | FALSE | 0.300 |
| Namhaeicola | NA | NA | NA | NA | NA | FALSE | NA |
| Nannocystis | NA | NA | NA | NA | NA | FALSE | NA |
| Neisseria | 0.5-0.8 µm | 0.4 µm | coccus | 0.500 | 0.400 | FALSE | 0.400 |
| Neisseria | 0.5 µm | 0.5 µm | coccus | 0.500 | 0.500 | FALSE | 0.500 |
| Neisseria | 1.55 µm | 1.55 µm | rod | 1.550 | 1.550 | FALSE | 1.550 |
| Neisseria | 4 µm | 1.25 µm | rod | 4.000 | 1.250 | FALSE | 1.250 |
| Neptuniibacter | 1.9 µm | 0.65 µm | rod | 1.900 | 0.650 | FALSE | 0.650 |
| Neptunomonas | 1.5 µm | 0.75 µm | rod | 1.500 | 0.750 | FALSE | 0.750 |
| Neptunomonas | 1.1 µm | 0.55 µm | rod | 1.100 | 0.550 | FALSE | 0.550 |
| Neptunomonas | 1.6-1.8 µm | 0.6-1 µm | rod | 1.600 | 0.600 | FALSE | 0.600 |
| Neptunomonas | 1.7 µm | 0.9 µm | rod | 1.700 | 0.900 | FALSE | 0.900 |
| Nisaea | 2.5 µm | 0.9 µm | rod | 2.500 | 0.900 | FALSE | 0.900 |
| Nisaea | 2.5 µm | 0.9 µm | rod | 2.500 | 0.900 | FALSE | 0.900 |
| Nitrosomonas | NA | NA | NA | NA | NA | FALSE | NA |
| Nonlabens | 03-05 µm | 0.5-0.7 µm | rod | 3.000 | 0.500 | FALSE | 0.500 |
| Nonlabens | 2.5 µm | 0.2-0.4 µm | rod | 2.500 | 0.200 | FALSE | 0.200 |
| Nonlabens | 0.5-10.0 µm | 0.5-0.6 µm | rod | 0.500 | 0.500 | FALSE | 0.500 |
| Novosphingobium | 1.0-2.0 µm | 0.1-0.5 µm | rod | 1.000 | 0.100 | FALSE | 0.100 |
| Novosphingobium | 1.0-1.5 µm | 0.3-0.5 µm | rod | 1.000 | 0.300 | FALSE | 0.300 |
| Novosphingobium | 2.0 µm | 1.5 µm | rod | 2.000 | 1.500 | FALSE | 1.500 |
| Novosphingobium | 1 µm | 0.7 µm | rod | 1.000 | 0.700 | FALSE | 0.700 |
| Novosphingobium | 0.8-1.5 µm | 0.5-0.6 µm | rod | 0.800 | 0.500 | FALSE | 0.500 |
| Novosphingobium | 1.0-3.0 µm | 0.5-0.8 µm | rod | 1.000 | 0.500 | FALSE | 0.500 |
| Novosphingobium | 0.8-1 µm | 0.3-0.5 µm | rod | 0.800 | 0.300 | FALSE | 0.300 |
| Novosphingobium | 0.8-2 µm | 0.3-0.5 µm | rod | 0.800 | 0.300 | FALSE | 0.300 |
| Novosphingobium | 1.3-1.9 µm | 0.25-0.55 µm | rod | 1.300 | 0.250 | FALSE | 0.250 |
| Novosphingobium | 1-1.3 µm | 0.2-0.4 µm | rod | 1.000 | 0.200 | FALSE | 0.200 |
| Novosphingobium | 1.0-1.5 µm | 0.5-0.7 µm | rod | 1.000 | 0.500 | FALSE | 0.500 |
| Novosphingobium | 0.8-2.0 µm | 0.5-0.8 µm | rod | 0.800 | 0.500 | FALSE | 0.500 |
| Novosphingobium | 0.9-4.2 µm | 0.6-0.8 µm | rod | 0.900 | 0.600 | FALSE | 0.600 |
| Novosphingobium | 2 µm | 0.8 µm | rod | 2.000 | 0.800 | FALSE | 0.800 |
| Novosphingobium | 2 µm | 0.9 µm | rod | 2.000 | 0.900 | FALSE | 0.900 |
| Novosphingobium | 1.3 µm | 0.7 µm | rod | 1.300 | 0.700 | FALSE | 0.700 |
| Novosphingobium | 1.7 µm | 0.8 µm | rod | 1.700 | 0.800 | FALSE | 0.800 |
| Novosphingobium | 1 µm | 1 µm | rod | 1.000 | 1.000 | FALSE | 1.000 |
| Novosphingobium | 1.6 µm | 0.8 µm | rod | 1.600 | 0.800 | FALSE | 0.800 |
| Novosphingobium | 1.25 µm | 0.65 µm | rod | 1.250 | 0.650 | FALSE | 0.650 |
| Novosphingobium | 1.4 µm | 0.58 µm | rod | 1.400 | 0.580 | FALSE | 0.580 |
| Novosphingobium | 1.5 µm | 0.6 µm | rod | 1.500 | 0.600 | FALSE | 0.600 |
| Novosphingobium | 1.4 µm | 0.8 µm | rod | 1.400 | 0.800 | FALSE | 0.800 |
| Novosphingobium | 2.9 µm | 0.45 µm | rod | 2.900 | 0.450 | FALSE | 0.450 |
| Novosphingobium | 1.05 µm | 0.4 µm | rod | 1.050 | 0.400 | FALSE | 0.400 |
| Novosphingobium | 0.97-1.95 µm | 0.36-0.45 µm | rod | 0.970 | 0.360 | FALSE | 0.360 |
| Oceanibaculum | 1.5-2.5 µm | 0.8-1 µm | rod | 1.500 | 0.800 | FALSE | 0.800 |
| Oceanibaculum | 2.4 µm | 1.05 µm | rod | 2.400 | 1.050 | FALSE | 1.050 |
| Oceanibaculum | 1.9 µm | 0.6 µm | rod | 1.900 | 0.600 | FALSE | 0.600 |
| Oceanicaulis | 1.1 µm | 0.65 µm | rod | 1.100 | 0.650 | FALSE | 0.650 |
| Oceanicola | 1.6 µm | 0.75 µm | rod | 1.600 | 0.750 | FALSE | 0.750 |
| Oceanococcus | NA | NA | NA | NA | NA | FALSE | NA |
| Oceanospirillum | NA | NA | NA | NA | NA | FALSE | NA |
| Odoribacter | 9.2 µm | 1.15 µm | rod | 9.200 | 1.150 | FALSE | 1.150 |
| Oleibacter | 2.15 µm | 0.45 µm | rod | 2.150 | 0.450 | FALSE | 0.450 |
| Oleiphilus | NA | NA | NA | NA | NA | FALSE | NA |
| Olleya | 2.25 µm | 0.4 µm | rod | 2.250 | 0.400 | FALSE | 0.400 |
| Ornithobacterium | NA | NA | NA | NA | NA | FALSE | NA |
| Owenweeksia | NA | NA | NA | NA | NA | FALSE | NA |
| Parachlamydia | NA | NA | NA | NA | NA | FALSE | NA |
| Paracoccus | 0.9-1.2 µm | 0.7-1.1 µm | rod | 0.900 | 0.700 | FALSE | 0.700 |
| Paracoccus | 1.2-1.5 µm | 0.7-0.9 µm | oval | 1.200 | 0.700 | FALSE | 0.700 |
| Paracoccus | 1.2-1.4 µm | 0.8-1.0 µm | rod | 1.200 | 0.800 | FALSE | 0.800 |
| Paracoccus | 0.4-1.5 µm | 0.4-0.7 µm | pleomorphic | 0.400 | 0.400 | FALSE | 0.400 |
| Paracoccus | 0.7-1.7 µm | 0.4-0.6 µm | rod | 0.700 | 0.400 | FALSE | 0.400 |
| Paracoccus | 1.2-1.4 µm | 0.6-0.7 µm | rod | 1.200 | 0.600 | FALSE | 0.600 |
| Paracoccus | 0.9 µm | 0.45 µm | rod | 0.900 | 0.450 | FALSE | 0.450 |
| Paracoccus | 1 µm | 0.65 µm | rod | 1.000 | 0.650 | FALSE | 0.650 |
| Paracoccus | 1.65 µm | 0.5 µm | rod | 1.650 | 0.500 | FALSE | 0.500 |
| Paracoccus | 0.9 µm | 0.45 µm | rod | 0.900 | 0.450 | FALSE | 0.450 |
| Paracoccus | 1 µm | 0.7 µm | rod | 1.000 | 0.700 | FALSE | 0.700 |
| Paracoccus | 1 µm | 1.75 µm | coccus | 1.000 | 1.750 | FALSE | 1.000 |
| Paracoccus | 0.7-2.5 µm | 0.85 µm | rod | 0.700 | 0.850 | FALSE | 0.700 |
| Paracoccus | 1.9 µm | 0.3-0.6 µm | rod | 1.900 | 0.300 | FALSE | 0.300 |
| Paracoccus | 1.75 µm | 0.6 µm | rod | 1.750 | 0.600 | FALSE | 0.600 |
| Paracoccus | 1 µm | 1.15 µm | ovoid | 1.000 | 1.150 | FALSE | 1.000 |
| Paracoccus | 1.1 µm | 1 µm | coccus | 1.100 | 1.000 | FALSE | 1.000 |
| Paracoccus | 4 µm | 0.6 µm | rod | 4.000 | 0.600 | FALSE | 0.600 |
| Parvibaculum | 1.2-1.4 µm | 0.5-0.6 µm | rod | 1.200 | 0.500 | FALSE | 0.500 |
| Parvibaculum | 1.4 µm | 0.4 µm | rod | 1.400 | 0.400 | FALSE | 0.400 |
| Parvibaculum | 1.4 µm | 0.45 µm | rod | 1.400 | 0.450 | FALSE | 0.450 |
| Parvularcula | 0.6-2.0 µm | 0.3-0.8 µm | rod | 0.600 | 0.300 | FALSE | 0.300 |
| Parvularcula | 2 µm | 0.55 µm | rod | 2.000 | 0.550 | FALSE | 0.550 |
| Parvularcula | 1.45 µm | 0.85 µm | rod | 1.450 | 0.850 | FALSE | 0.850 |
| Pelagibacterium | 1.0-1.5 µm | 0.4-0.8 µm | rod | 1.000 | 0.400 | FALSE | 0.400 |
| Pelagibacterium | 0.8-1.3 µm | 0.3-0.6 µm | rod | 0.800 | 0.300 | FALSE | 0.300 |
| Pelagibacterium | 0.7-1.1 µm | 0.6-0.7 µm | rod | 0.700 | 0.600 | FALSE | 0.600 |
| Pelagibacterium | 1.2-2.5 µm | 0.5-0.6 µm | rod | 1.200 | 0.500 | FALSE | 0.500 |
| Pelagibius | 1.85 µm | 0.75 µm | rod | 1.850 | 0.750 | FALSE | 0.750 |
| Pelagicoccus | NA | NA | NA | NA | NA | FALSE | NA |
| Peredibacter | NA | NA | NA | NA | NA | FALSE | NA |
| Perspicuibacter | NA | NA | NA | NA | NA | FALSE | NA |
| Phaeocystidibacter | 1.25 µm | 0.3 µm | rod | 1.250 | 0.300 | FALSE | 0.300 |
| Phaeodactylibacter | NA | NA | NA | NA | NA | FALSE | NA |
| Phenylobacterium | 1.2-1.6 µm | 0.5-0.6 µm | rod | 1.200 | 0.500 | FALSE | 0.500 |
| Phenylobacterium | 0.8-1 µm | 0.2-0.3 µm | rod | 0.800 | 0.200 | FALSE | 0.200 |
| Phenylobacterium | 1.4-2.9 µm | 0.6-1.7 µm | rod | 1.400 | 0.600 | FALSE | 0.600 |
| Phenylobacterium | 0.4-1.3 µm | 0.4-0.5 µm | rod | 0.400 | 0.400 | FALSE | 0.400 |
| Phenylobacterium | 2.5-4.0 µm | 0.4-0.6 µm | rod | 2.500 | 0.400 | FALSE | 0.400 |
| Phenylobacterium | 0.7-1.3 µm | 0.5-0.6 µm | ovoid | 0.700 | 0.500 | FALSE | 0.500 |
| Phenylobacterium | 0.8-1.8 µm | 0.5 µm | rod | 0.800 | 0.500 | FALSE | 0.500 |
| Phenylobacterium | 2 µm | 0.5 µm | rod | 2.000 | 0.500 | FALSE | 0.500 |
| Phycisphaera | NA | NA | NA | NA | NA | FALSE | NA |
| Phyllobacterium | 0.9-3.5 µm | 0.5-0.9 µm | rod | 0.900 | 0.500 | FALSE | 0.500 |
| Phyllobacterium | 1.15 µm | 0.55 µm | rod | 1.150 | 0.550 | FALSE | 0.550 |
| Pibocella | NA | NA | NA | NA | NA | FALSE | NA |
| Pirellula | NA | NA | NA | NA | NA | FALSE | NA |
| Planctomicrobium | NA | NA | NA | NA | NA | FALSE | NA |
| Plesiocystis | NA | NA | NA | NA | NA | FALSE | NA |
| Polaribacter | 1.1-1.6 µm | 0.4-0.5 µm | rod | 1.100 | 0.400 | FALSE | 0.400 |
| Polaribacter | 1.39 µm | 0.37 µm | rod | 1.390 | 0.370 | FALSE | 0.370 |
| Polaribacter | 1.7-2.0 µm | 0.6-1.0 µm | rod | 1.700 | 0.600 | FALSE | 0.600 |
| Polaribacter | 0.8-5 µm | 0.4-0.6 µm | rod | 0.800 | 0.400 | FALSE | 0.400 |
| Ponticaulis | NA | NA | NA | NA | NA | FALSE | NA |
| Portibacter | NA | NA | NA | NA | NA | FALSE | NA |
| Prevotella | 3 µm | 0.8 µm | rod | 3.000 | 0.800 | FALSE | 0.800 |
| Prevotella | 1.6 µm | 0.65 µm | rod | 1.600 | 0.650 | FALSE | 0.650 |
| Prevotella | 0.8-3 µm | 0.7 µm | rod | 0.800 | 0.700 | FALSE | 0.700 |
| Prevotella | 1.7 µm | 0.75 µm | rod | 1.700 | 0.750 | FALSE | 0.750 |
| Prevotella | 2 µm | 0.7 µm | rod | 2.000 | 0.700 | FALSE | 0.700 |
| Prevotella | 1.1 µm | 0.4 µm | rod | 1.100 | 0.400 | FALSE | 0.400 |
| Prevotella | 0.75 µm | 0.45 µm | rod | 0.750 | 0.450 | FALSE | 0.450 |
| Prevotella | 1.16 µm | 0.33 µm | rod | 1.160 | 0.330 | FALSE | 0.330 |
| Prevotella | 0.95 µm | 0.83 µm | rod | 0.950 | 0.830 | FALSE | 0.830 |
| Prevotella | 0.8-6 µm | 0.7-0.8 µm | rod | 0.800 | 0.700 | FALSE | 0.700 |
| Prevotella | 5.4 µm | 0.8 µm | coccus | 5.400 | 0.800 | FALSE | 0.800 |
| Prevotella | 1.95 µm | 0.4 µm | rod | 1.950 | 0.400 | FALSE | 0.400 |
| Prevotella | 1.3 µm | 0.6 µm | ovoid | 1.300 | 0.600 | FALSE | 0.600 |
| Prevotella | 9.15 µm | 0.65 µm | rod | 9.150 | 0.650 | FALSE | 0.650 |
| Prevotella | 1.25 µm | 0.65 µm | rod | 1.250 | 0.650 | FALSE | 0.650 |
| Propionigenium | NA | NA | NA | NA | NA | FALSE | NA |
| Propionivibrio | NA | NA | NA | NA | NA | FALSE | NA |
| Pseudoalteromonas | 0.5-3.5 µm | 0.2-0.7 µm | rod | 0.500 | 0.200 | FALSE | 0.200 |
| Pseudoalteromonas | 1.8-3 µm | 0.8-1.3 µm | rod | 1.800 | 0.800 | FALSE | 0.800 |
| Pseudoalteromonas | 1.4-2.6 µm | 0.5-0.8 µm | rod | 1.400 | 0.500 | FALSE | 0.500 |
| Pseudoalteromonas | 1.3-4.0 µm | 0.4-1.3 µm | rod | 1.300 | 0.400 | FALSE | 0.400 |
| Pseudoalteromonas | 1.35 µm | 0.5 µm | rod | 1.350 | 0.500 | FALSE | 0.500 |
| Pseudoalteromonas | 1.25 µm | 0.65 µm | rod | 1.250 | 0.650 | FALSE | 0.650 |
| Pseudoalteromonas | 1.1-2.8 µm | 0.4-0.7 µm | rod | 1.100 | 0.400 | FALSE | 0.400 |
| Pseudoalteromonas | 1.5-2.2 µm | 0.4-0.5 µm | rod | 1.500 | 0.400 | FALSE | 0.400 |
| Pseudoalteromonas | 01-02 µm | 0.5-0.8 µm | rod | 1.000 | 0.500 | FALSE | 0.500 |
| Pseudoalteromonas | 0.4-0.7 µm | 0.7-0.9 µm | rod | 0.400 | 0.700 | FALSE | 0.400 |
| Pseudoalteromonas | 1.5-3.0 µm | 0.5-0.8 µm | rod | 1.500 | 0.500 | FALSE | 0.500 |
| Pseudobacteriovorax | NA | NA | NA | NA | NA | FALSE | NA |
| Pseudofulvibacter | 1.8-2.3 µm | 0.4-0.6 µm | rod | 1.800 | 0.400 | FALSE | 0.400 |
| Pseudofulvibacter | 1.2-2 µm | 0.3 µm | rod | 1.200 | 0.300 | FALSE | 0.300 |
| Pseudofulvibacter | 2.9 µm | 0.45 µm | rod | 2.900 | 0.450 | FALSE | 0.450 |
| Pseudohongiella | 1.3-1.7 µm | 0.5-0.7 µm | rod | 1.300 | 0.500 | FALSE | 0.500 |
| Pseudomonas | NA | NA | NA | NA | NA | FALSE | NA |
| Pseudoruegeria | 0.4-10.0 µm | 0.4-0.8 µm | rod | 0.400 | 0.400 | FALSE | 0.400 |
| Pseudoruegeria | 2.4-2.5 µm | 0.5-0.6 µm | rod | 2.400 | 0.500 | FALSE | 0.500 |
| Pseudoruegeria | 2.8 µm | 0.65 µm | rod | 2.800 | 0.650 | FALSE | 0.650 |
| Pseudoteredinibacter | NA | NA | NA | NA | NA | FALSE | NA |
| Pseudozobellia | NA | NA | NA | NA | NA | FALSE | NA |
| Psychrobacter | 0.7-1.3 µm | 0.5-0.8 µm | coccus | 0.700 | 0.500 | FALSE | 0.500 |
| Psychrobacter | 1.15 µm | 0.65 µm | coccus | 1.150 | 0.650 | FALSE | 0.650 |
| Psychrobacter | 1.45 µm | 1 µm | coccus | 1.450 | 1.000 | FALSE | 1.000 |
| Psychrobacter | 1.75 µm | 0.8 µm | ovoid | 1.750 | 0.800 | FALSE | 0.800 |
| Psychrobacter | 1.6-1.9 µm | 0.9-1.1 µm | ovoid | 1.600 | 0.900 | FALSE | 0.900 |
| Psychrobacter | 1.0-2.0 µm | 0.5-1.0 µm | ovoid | 1.000 | 0.500 | FALSE | 0.500 |
| Psychrobacter | 1.62 µm | 0.73 µm | rod | 1.620 | 0.730 | FALSE | 0.730 |
| Psychrobacter | 0.9-1.3 µm | 0.5-0.8 µm | ovoid | 0.900 | 0.500 | FALSE | 0.500 |
| Psychrobacter | 1.55 µm | 1.25 µm | ovoid | 1.550 | 1.250 | FALSE | 1.250 |
| Psychrobium | NA | NA | NA | NA | NA | FALSE | NA |
| Psychroflexus | 0.6-2.0 µm | 0.2-0.5 µm | rod | 0.600 | 0.200 | FALSE | 0.200 |
| Psychroflexus | 1.2-2.2 µm | 0.3-0.6 µm | rod | 1.200 | 0.300 | FALSE | 0.300 |
| Psychroflexus | 0.3-10.0 µm | 0.2-0.5 µm | rod | 0.300 | 0.200 | FALSE | 0.200 |
| Psychroflexus | 3.0-6.0 µm | 0.4-1.0 µm | rod | 3.000 | 0.400 | FALSE | 0.400 |
| Psychroflexus | 3 µm | 0.55 µm | rod | 3.000 | 0.550 | FALSE | 0.550 |
| Psychroflexus | 2.25 µm | 0.21 µm | rod | 2.250 | 0.210 | FALSE | 0.210 |
| Psychrosphaera | NA | NA | rod | NA | NA | FALSE | NA |
| Ralstonia | 1.5-3.0 µm | 1.0 µm | rod | 1.500 | 1.000 | FALSE | 1.000 |
| Reichenbachiella | NA | NA | NA | NA | NA | FALSE | NA |
| Reinekea | 2.1-3.1 µm | 0.15-0.3 µm | rod | 2.100 | 0.150 | FALSE | 0.150 |
| Reinekea | 2.0-4.0 µm | 0.2-0.4 µm | rod | 2.000 | 0.200 | FALSE | 0.200 |
| Reinekea | 2 µm | 0.5 µm | rod | 2.000 | 0.500 | FALSE | 0.500 |
| Reinekea | 1.6 µm | 0.45 µm | rod | 1.600 | 0.450 | FALSE | 0.450 |
| Rhizobium | 1.58 µm | 0.5 µm | rod | 1.580 | 0.500 | TRUE | 0.500 |
| Rhizobium | 3.5 µm | 0.9 µm | rod | 3.500 | 0.900 | TRUE | 0.900 |
| Rhizobium | 2.4 µm | 1 µm | rod | 2.400 | 1.000 | TRUE | 1.000 |
| Rhizobium | 2 µm | 0.55 µm | rod | 2.000 | 0.550 | TRUE | 0.550 |
| Rhizobium | 01-03 µm | 0.3-0.5 µm | rod | 1.000 | 0.300 | TRUE | 0.300 |
| Rhizobium | 2-2.5 µm | 0.5-0.7 µm | rod | 2.000 | 0.500 | TRUE | 0.500 |
| Rhizobium | 1.2 µm | 0.65 µm | rod | 1.200 | 0.650 | TRUE | 0.650 |
| Rhodopirellula | 1.7-2.3 µm | 1.1-1.5 µm | other | 1.700 | 1.100 | FALSE | 1.100 |
| Rhodopirellula | 1.9-2.7 µm | 0.8-1.2 µm | other | 1.900 | 0.800 | FALSE | 0.800 |
| Rhodopirellula | 2.1-2.7 µm | 1.7-2.1 µm | pleomorphic | 2.100 | 1.700 | FALSE | 1.700 |
| Rhodopirellula | 1.75 µm | 1.75 µm | ovoid | 1.750 | 1.750 | FALSE | 1.750 |
| Rhodopseudomonas | 2-4.5 µm | 0.8-1.5 µm | rod | 2.000 | 0.800 | FALSE | 0.800 |
| Rhodopseudomonas | 2.75 µm | 0.9 µm | rod | 2.750 | 0.900 | FALSE | 0.900 |
| Rhodopseudomonas | 4 µm | 0.9 µm | rod | 4.000 | 0.900 | FALSE | 0.900 |
| Rhodospirillum | NA | NA | NA | NA | NA | TRUE | NA |
| Rhodovibrio | NA | NA | NA | NA | NA | FALSE | NA |
| Rhodovulum | 1.75 µm | 0.6 µm | rod | 1.750 | 0.600 | FALSE | 0.600 |
| Rhodovulum | 1.75 µm | 0.55 µm | rod | 1.750 | 0.550 | FALSE | 0.550 |
| Rhodovulum | 1.5-1.8 µm | 0.5-1 µm | rod | 1.500 | 0.500 | FALSE | 0.500 |
| Rhodovulum | 1.2 µm | 0.9 µm | rod | 1.200 | 0.900 | FALSE | 0.900 |
| Rhodovulum | 1 µm | 0.5 µm | rod | 1.000 | 0.500 | FALSE | 0.500 |
| Rhodovulum | 01-02 µm | 0.6-0.8 µm | ovoid | 1.000 | 0.600 | FALSE | 0.600 |
| Rickettsia | 1.5 µm | 0.3 µm | rod | 1.500 | 0.300 | FALSE | 0.300 |
| Rickettsia | 1.4 µm | 0.4 µm | rod | 1.400 | 0.400 | FALSE | 0.400 |
| Rickettsia | 0.6-2 µm | 0.4 µm | rod | 0.600 | 0.400 | FALSE | 0.400 |
| Rickettsia | 0.832 µm | 0.427 µm | rod | 0.832 | 0.427 | FALSE | 0.427 |
| Rivicola | NA | NA | NA | NA | NA | FALSE | NA |
| Robiginitalea | NA | NA | NA | NA | NA | FALSE | NA |
| Romboutsia | NA | NA | NA | NA | NA | FALSE | NA |
| Roseburia | NA | NA | NA | NA | NA | FALSE | NA |
| Roseibacillus | NA | NA | NA | NA | NA | FALSE | NA |
| Roseibium | 2.0-4.0 µm | 0.5-0.8 µm | rod | 2.000 | 0.500 | FALSE | 0.500 |
| Roseibium | 1.0-2.5 µm | 0.5-1.0 µm | rod | 1.000 | 0.500 | FALSE | 0.500 |
| Roseibium | 1.95 µm | 0.6 µm | rod | 1.950 | 0.600 | FALSE | 0.600 |
| Roseicyclus | 1-2.5 µm | 0.4-0.8 µm | rod | 1.000 | 0.400 | FALSE | 0.400 |
| Roseivirga | 2.0-9.0 µm | 0.4-1.0 µm | rod | 2.000 | 0.400 | FALSE | 0.400 |
| Roseivirga | 1-2 µm | 0.5-0.8 µm | rod | 1.000 | 0.500 | FALSE | 0.500 |
| Roseivirga | 2 µm | 0.5 µm | rod | 2.000 | 0.500 | FALSE | 0.500 |
| Roseivirga | 1.5 µm | 0.5 µm | rod | 1.500 | 0.500 | FALSE | 0.500 |
| Roseivirga | 2.65 µm | 0.4 µm | rod | 2.650 | 0.400 | FALSE | 0.400 |
| Roseivirga | 3.25 µm | 0.6 µm | rod | 3.250 | 0.600 | FALSE | 0.600 |
| Roseivivax | 1.5 µm | 0.85 µm | rod | 1.500 | 0.850 | FALSE | 0.850 |
| Roseovarius | 0.9-2.6 µm | 0.3-1.0 µm | rod | 0.900 | 0.300 | FALSE | 0.300 |
| Roseovarius | 8.1-10.9 µm | 2.5-3.4 µm | rod | 8.100 | 2.500 | FALSE | 2.500 |
| Roseovarius | 2-5.5 µm | 0.3-0.4 µm | rod | 2.000 | 0.300 | FALSE | 0.300 |
| Roseovarius | 1.2-1.8 µm | 0.7-0.9 µm | rod | 1.200 | 0.700 | FALSE | 0.700 |
| Roseovarius | 1.42-3.37 µm | 0.58-0.84 µm | ovoid | 1.420 | 0.580 | FALSE | 0.580 |
| Roseovarius | 1.34 µm | 0.53 µm | rod | 1.340 | 0.530 | FALSE | 0.530 |
| Roseovarius | 2.5 µm | 0.6 µm | ovoid | 2.500 | 0.600 | FALSE | 0.600 |
| Roseovarius | 2.15 µm | 0.6 µm | rod | 2.150 | 0.600 | FALSE | 0.600 |
| Rubinisphaera | 1.4-1.8 µm | 0.7-0.9 µm | other | 1.400 | 0.700 | FALSE | 0.700 |
| Rubricoccus | NA | NA | NA | NA | NA | FALSE | NA |
| Rubritalea | 1.1-1.4 µm | 0.9-1 µm | rod | 1.100 | 0.900 | FALSE | 0.900 |
| Ruegeria | 1-3 µm | 0.5-1 µm | rod | 1.000 | 0.500 | FALSE | 0.500 |
| Ruegeria | 2.0-5.0 µm | 0.6-1.2 µm | rod | 2.000 | 0.600 | FALSE | 0.600 |
| Ruegeria | 3.5 µm | 1.1 µm | coccus | 3.500 | 1.100 | FALSE | 1.100 |
| Ruegeria | 0.75 µm | 0.4 µm | rod | 0.750 | 0.400 | FALSE | 0.400 |
| Ruegeria | 3.25 µm | 0.75 µm | rod | 3.250 | 0.750 | FALSE | 0.750 |
| Ruminococcus | NA | 0.9-1.3 µm | coccus | NA | 0.900 | FALSE | NA |
| Ruminococcus2 | NA | NA | NA | NA | NA | FALSE | NA |
| Saccharicrinis | NA | NA | NA | NA | NA | FALSE | NA |
| Saccharophagus | NA | NA | NA | NA | NA | FALSE | NA |
| Salinibacter | NA | NA | NA | NA | NA | FALSE | NA |
| Salinirepens | 18 µm | 0.55 µm | rod | 18.000 | 0.550 | FALSE | 0.550 |
| Salinisphaera | 1.2-1.6 µm | 0.6-0.7 µm | rod | 1.200 | 0.600 | FALSE | 0.600 |
| Salinisphaera | 1.4 µm | 1 µm | rod | 1.400 | 1.000 | FALSE | 1.000 |
| Salinisphaera | 1.75 µm | 0.6 µm | rod | 1.750 | 0.600 | FALSE | 0.600 |
| Salinisphaera | 1.15 µm | 0.5 µm | rod | 1.150 | 0.500 | FALSE | 0.500 |
| Salinisphaera | 0.9 µm | 0.4 µm | rod | 0.900 | 0.400 | FALSE | 0.400 |
| Salinisphaera | 0.7 µm | 0.45 µm | rod | 0.700 | 0.450 | FALSE | 0.450 |
| Salinispira | NA | NA | NA | NA | NA | FALSE | NA |
| Salisaeta | 22.5 µm | NA | rod | 22.500 | NA | FALSE | NA |
| Sandaracinus | 4.5 µm | 0.85 µm | rod | 4.500 | 0.850 | FALSE | 0.850 |
| Saprospira | NA | NA | NA | NA | NA | FALSE | NA |
| Sebaldella | NA | NA | NA | NA | NA | FALSE | NA |
| Sediminibacter | 1.0-2.5 µm | 0.5-0.7 µm | rod | 1.000 | 0.500 | FALSE | 0.500 |
| Seonamhaeicola | 1-4 µm | 0.3-0.8 µm | rod | 1.000 | 0.300 | FALSE | 0.300 |
| Seonamhaeicola | 2-8 µm | 0.5-0.7 µm | rod | 2.000 | 0.500 | FALSE | 0.500 |
| Seonamhaeicola | 1.0-12.0 µm | 0.3-0.5 µm | rod | 1.000 | 0.300 | FALSE | 0.300 |
| Shewanella | 1.5-2.5 µm | 0.5-1.0 µm | rod | 1.500 | 0.500 | FALSE | 0.500 |
| Shewanella | 1.7-4 µm | 0.5-0.7 µm | rod | 1.700 | 0.500 | FALSE | 0.500 |
| Shewanella | 1.1 µm | 0.6 µm | rod | 1.100 | 0.600 | FALSE | 0.600 |
| Shewanella | 1.1 µm | 0.6 µm | rod | 1.100 | 0.600 | FALSE | 0.600 |
| Shewanella | 3.5 µm | 0.65 µm | rod | 3.500 | 0.650 | FALSE | 0.650 |
| Shewanella | 3.5 µm | 0.65 µm | rod | 3.500 | 0.650 | FALSE | 0.650 |
| Shewanella | 1.25 µm | 0.65 µm | rod | 1.250 | 0.650 | FALSE | 0.650 |
| Shewanella | 1-1.2 µm | 0.5-0.7 µm | rod | 1.000 | 0.500 | FALSE | 0.500 |
| Shewanella | 1-1.2 µm | 0.5-0.7 µm | rod | 1.000 | 0.500 | FALSE | 0.500 |
| Shewanella | 3 µm | 0.5 µm | rod | 3.000 | 0.500 | FALSE | 0.500 |
| Shewanella | 3 µm | 0.75 µm | rod | 3.000 | 0.750 | FALSE | 0.750 |
| Shewanella | 2.75 µm | 0.95 µm | rod | 2.750 | 0.950 | FALSE | 0.950 |
| Shewanella | 2.85 µm | 0.65 µm | rod | 2.850 | 0.650 | FALSE | 0.650 |
| Shewanella | 1.75 µm | 0.65 µm | rod | 1.750 | 0.650 | FALSE | 0.650 |
| Shewanella | 2.5 µm | 0.75 µm | rod | 2.500 | 0.750 | FALSE | 0.750 |
| Shewanella | 1.75 µm | 0.75 µm | rod | 1.750 | 0.750 | FALSE | 0.750 |
| Shewanella | 2.25 µm | 0.5 µm | rod | 2.250 | 0.500 | FALSE | 0.500 |
| Shewanella | 1.5-3.5 µm | 0.5-0.8 µm | rod | 1.500 | 0.500 | FALSE | 0.500 |
| Shewanella | 1.6-3.8 µm | 0.4-0.8 µm | rod | 1.600 | 0.400 | FALSE | 0.400 |
| Shewanella | 1.7 µm | 0.5 µm | rod | 1.700 | 0.500 | FALSE | 0.500 |
| Shewanella | 2 µm | 0.8-1 µm | rod | 2.000 | 0.800 | FALSE | 0.800 |
| Shewanella | 2.5-4 µm | 0.7-0.8 µm | rod | 2.500 | 0.700 | FALSE | 0.700 |
| Shewanella | 1.5 µm | 0.7 µm | rod | 1.500 | 0.700 | FALSE | 0.700 |
| Shewanella | 3.5 µm | 0.55 µm | rod | 3.500 | 0.550 | FALSE | 0.550 |
| Shewanella | 2.5 µm | 0.7 µm | rod | 2.500 | 0.700 | FALSE | 0.700 |
| Shewanella | 1.8 µm | 0.7 µm | rod | 1.800 | 0.700 | FALSE | 0.700 |
| Shewanella | 02-03 µm | 0.55-0.65 µm | rod | 2.000 | 0.550 | FALSE | 0.550 |
| Shewanella | 3.2-4 µm | 0.4-0.6 µm | rod | 3.200 | 0.400 | FALSE | 0.400 |
| Shewanella | 2-2.5 µm | 0.8-1 µm | rod | 2.000 | 0.800 | FALSE | 0.800 |
| Shewanella | 2.5-3 µm | 0.8-1 µm | rod | 2.500 | 0.800 | FALSE | 0.800 |
| Shewanella | 1.7 µm | 0.7 µm | rod | 1.700 | 0.700 | FALSE | 0.700 |
| Shewanella | 2.25 µm | 0.6 µm | rod | 2.250 | 0.600 | FALSE | 0.600 |
| Shewanella | 3 µm | 0.6 µm | rod | 3.000 | 0.600 | FALSE | 0.600 |
| Shewanella | 1.5 µm | 0.7 µm | rod | 1.500 | 0.700 | FALSE | 0.700 |
| Shimia | 1.85 µm | 0.6 µm | rod | 1.850 | 0.600 | FALSE | 0.600 |
| Shimia | 1.5-2 µm | 0.7-1 µm | rod | 1.500 | 0.700 | FALSE | 0.700 |
| Shimia | 2 µm | 0.8 µm | pleomorphic | 2.000 | 0.800 | FALSE | 0.800 |
| Shimia | 0.8-3.6 µm | 0.3-0.6 µm | rod | 0.800 | 0.300 | FALSE | 0.300 |
| Shimia | 2.2 µm | 0.95 µm | rod | 2.200 | 0.950 | FALSE | 0.950 |
| Simkania | NA | NA | NA | NA | NA | FALSE | NA |
| Sinobacterium | 2 µm | 0.6 µm | rod | 2.000 | 0.600 | FALSE | 0.600 |
| Solitalea | 2.2-12 µm | 0.4-0.5 µm | rod | 2.200 | 0.400 | FALSE | 0.400 |
| Solitalea | 15.65 µm | 0.55 µm | rod | 15.650 | 0.550 | FALSE | 0.550 |
| Sphingobium | 1.2-1.7 µm | 0.7-1.0 µm | rod | 1.200 | 0.700 | FALSE | 0.700 |
| Sphingobium | 0.8-1.0 µm | 0.5-0.7 µm | rod | 0.800 | 0.500 | FALSE | 0.500 |
| Sphingobium | 1.0-1.2 µm | 0.8-0.9 µm | rod | 1.000 | 0.800 | FALSE | 0.800 |
| Sphingobium | 1.0-1.2 µm | 0.6-0.8 µm | rod | 1.000 | 0.600 | FALSE | 0.600 |
| Sphingobium | 1.5 µm | 0.6-0.7 µm | rod | 1.500 | 0.600 | FALSE | 0.600 |
| Sphingobium | 2 µm | 0.5 µm | rod | 2.000 | 0.500 | FALSE | 0.500 |
| Sphingobium | 1.6 µm | 0.4 µm | rod | 1.600 | 0.400 | FALSE | 0.400 |
| Sphingobium | 1-1.2 µm | 0.6-0.8 µm | rod | 1.000 | 0.600 | FALSE | 0.600 |
| Sphingobium | 0.95 µm | 0.4 µm | rod | 0.950 | 0.400 | FALSE | 0.400 |
| Sphingobium | 1.75 µm | 0.45 µm | rod | 1.750 | 0.450 | FALSE | 0.450 |
| Sphingobium | 1.4 µm | 0.5 µm | rod | 1.400 | 0.500 | FALSE | 0.500 |
| Sphingobium | 1-1.2 µm | 0.6-0.8 µm | rod | 1.000 | 0.600 | FALSE | 0.600 |
| Sphingobium | 1.15 µm | 0.55 µm | rod | 1.150 | 0.550 | FALSE | 0.550 |
| Sphingobium | 2.15 µm | 1 µm | rod | 2.150 | 1.000 | FALSE | 1.000 |
| Sphingobium | 1.8 µm | 0.55 µm | rod | 1.800 | 0.550 | FALSE | 0.550 |
| Sphingobium | 0.95 µm | 0.44 µm | rod | 0.950 | 0.440 | FALSE | 0.440 |
| Sphingobium | 2.6 µm | 1.3 µm | rod | 2.600 | 1.300 | FALSE | 1.300 |
| Sphingobium | 1.15 µm | 0.5 µm | rod | 1.150 | 0.500 | FALSE | 0.500 |
| Sphingomonas | 1.8 µm | 0.55 µm | rod | 1.800 | 0.550 | TRUE | 0.550 |
| Sphingomonas | 1.4 µm | 0.45 µm | spiral | 1.400 | 0.450 | TRUE | 0.450 |
| Sphingomonas | 2.25 µm | 0.45 µm | rod | 2.250 | 0.450 | TRUE | 0.450 |
| Sphingomonas | 1.5 µm | 0.6 µm | rod | 1.500 | 0.600 | TRUE | 0.600 |
| Sphingomonas | 1.5 µm | 0.55 µm | rod | 1.500 | 0.550 | TRUE | 0.550 |
| Sphingomonas | 1.3 µm | 0.5 µm | rod | 1.300 | 0.500 | TRUE | 0.500 |
| Sphingomonas | 1.5 µm | 0.55 µm | rod | 1.500 | 0.550 | TRUE | 0.550 |
| Sphingomonas | 1.4 µm | 0.4 µm | rod | 1.400 | 0.400 | TRUE | 0.400 |
| Sphingomonas | 1.2 µm | 0.7 µm | rod | 1.200 | 0.700 | TRUE | 0.700 |
| Sphingomonas | 2.35 µm | 0.25 µm | rod | 2.350 | 0.250 | TRUE | 0.250 |
| Sphingomonas | 0.8 µm | 0.5 µm | rod | 0.800 | 0.500 | TRUE | 0.500 |
| Sphingomonas | 2 µm | 0.35 µm | rod | 2.000 | 0.350 | TRUE | 0.350 |
| Sphingomonas | 1.65 µm | 0.7 µm | rod | 1.650 | 0.700 | TRUE | 0.700 |
| Sphingomonas | 1.75 µm | 0.75 µm | rod | 1.750 | 0.750 | TRUE | 0.750 |
| Sphingomonas | 1.2 µm | 0.5 µm | rod | 1.200 | 0.500 | TRUE | 0.500 |
| Sphingomonas | 1.2 µm | 0.7 µm | rod | 1.200 | 0.700 | TRUE | 0.700 |
| Sphingomonas | 0.85 µm | 0.45 µm | rod | 0.850 | 0.450 | TRUE | 0.450 |
| Sphingomonas | 0.8-1.5 µm | 0.4-0.6 µm | rod | 0.800 | 0.400 | TRUE | 0.400 |
| Sphingomonas | 1.7 µm | 0.75 µm | rod | 1.700 | 0.750 | TRUE | 0.750 |
| Sphingomonas | 1.0-1.5 µm | 0.4-0.6 µm | rod | 1.000 | 0.400 | TRUE | 0.400 |
| Sphingomonas | 01-03 µm | 0.5-1 µm | rod | 1.000 | 0.500 | TRUE | 0.500 |
| Sphingomonas | 0.9 µm | 0.55 µm | rod | 0.900 | 0.550 | TRUE | 0.550 |
| Sphingomonas | 2.25 µm | 0.45 µm | rod | 2.250 | 0.450 | TRUE | 0.450 |
| Sphingomonas | 1.6 µm | 0.6 µm | rod | 1.600 | 0.600 | TRUE | 0.600 |
| Sphingomonas | 1.7 µm | 0.7 µm | rod | 1.700 | 0.700 | TRUE | 0.700 |
| Sphingomonas | 1.25 µm | 0.5 µm | rod | 1.250 | 0.500 | TRUE | 0.500 |
| Sphingomonas | 0.5 µm | 0.25 µm | rod | 0.500 | 0.250 | TRUE | 0.250 |
| Sphingomonas | 2.5 µm | 0.5 µm | rod | 2.500 | 0.500 | TRUE | 0.500 |
| Spirochaeta | 8.0-18.0 µm | 0.23 µm | rod | 8.000 | 0.230 | FALSE | 0.230 |
| Spongiibacterium | NA | NA | NA | NA | NA | FALSE | NA |
| Sporocytophaga | NA | NA | NA | NA | NA | FALSE | NA |
| Staphylococcus | NA | NA | NA | NA | NA | FALSE | NA |
| Stenotrophomonas | 1.5-2.5 µm | 0.8 µm | rod | 1.500 | 0.800 | FALSE | 0.800 |
| Stenotrophomonas | 0.92-1.55 µm | 0.45-0.65 µm | rod | 0.920 | 0.450 | FALSE | 0.450 |
| Stenotrophomonas | 1.9-3.1 µm | 0.8-1.2 µm | rod | 1.900 | 0.800 | FALSE | 0.800 |
| Stenotrophomonas | 1.5 µm | 0.5 µm | rod | 1.500 | 0.500 | FALSE | 0.500 |
| Stenotrophomonas | 1.5-3 µm | 0.5-0.8 µm | rod | 1.500 | 0.500 | FALSE | 0.500 |
| Sulfurimonas | 2.1 µm | 0.66 µm | spiral | 2.100 | 0.660 | FALSE | 0.660 |
| Sulfurimonas | 1.5-2.5 µm | 0.6-0.8 µm | rod | 1.500 | 0.600 | FALSE | 0.600 |
| Sulfurovum | 1.05 µm | 0.4 µm | rod | 1.050 | 0.400 | FALSE | 0.400 |
| Sulfurovum | 0.6-1.4 µm | 0.4 µm | rod | 0.600 | 0.400 | FALSE | 0.400 |
| Sulfurovum | 0.5-1.2 µm | 0.4-0.8 µm | ovoid | 0.500 | 0.400 | FALSE | 0.400 |
| Sutterella | 1.7-2.1 µm | 0.7 µm | coccus | 1.700 | 0.700 | FALSE | 0.700 |
| Tamlana | 1.2-3.5 µm | 0.6-1 µm | rod | 1.200 | 0.600 | FALSE | 0.600 |
| Tenacibaculum | 4.8-8 µm | 0.5-0.6 µm | rod | 4.800 | 0.500 | FALSE | 0.500 |
| Tenacibaculum | 1.5-2.3 µm | 0.2-0.4 µm | rod | 1.500 | 0.200 | FALSE | 0.200 |
| Tenacibaculum | 0.8-10 µm | 0.2-0.5 µm | rod | 0.800 | 0.200 | FALSE | 0.200 |
| Tenacibaculum | 0.8-6 µm | 0.3-0.5 µm | rod | 0.800 | 0.300 | FALSE | 0.300 |
| Tenacibaculum | 1.0-4.0 µm | 0.2-0.4 µm | rod | 1.000 | 0.200 | FALSE | 0.200 |
| Tenacibaculum | 0.7-10 µm | 0.1-0.3 µm | rod | 0.700 | 0.100 | FALSE | 0.100 |
| Tenacibaculum | 0.4-10 µm | 0.1-0.4 µm | rod | 0.400 | 0.100 | FALSE | 0.100 |
| Tenacibaculum | 0.4-10 µm | 0.5 µm | rod | 0.400 | 0.500 | FALSE | 0.400 |
| Tenacibaculum | 14 µm | 0.5 µm | rod | 14.000 | 0.500 | FALSE | 0.500 |
| Tenacibaculum | 4.5 µm | 0.4 µm | rod | 4.500 | 0.400 | FALSE | 0.400 |
| Tenacibaculum | 6 µm | 0.3-0.5 µm | rod | 6.000 | 0.300 | FALSE | 0.300 |
| Tenacibaculum | 02-35 µm | 0.3 µm | rod | 2.000 | 0.300 | FALSE | 0.300 |
| Tenacibaculum | 6.5 µm | 0.2-0.4 µm | rod | 6.500 | 0.200 | FALSE | 0.200 |
| Tenacibaculum | 1.0-9.0 µm | 0.3-0.5 µm | rod | 1.000 | 0.300 | FALSE | 0.300 |
| Tenacibaculum | 02-40 µm | 0.3 µm | rod | 2.000 | 0.300 | FALSE | 0.300 |
| Tenacibaculum | 3.25 µm | 0.5 µm | rod | 3.250 | 0.500 | FALSE | 0.500 |
| Tenacibaculum | 16 µm | 0.3 µm | rod | 16.000 | 0.300 | FALSE | 0.300 |
| Tenacibaculum | 2.5 µm | 0.5 µm | rod | 2.500 | 0.500 | FALSE | 0.500 |
| Tenacibaculum | 16 µm | 0.5 µm | rod | 16.000 | 0.500 | FALSE | 0.500 |
| Tenacibaculum | 2-20 µm | 0.4-0.5 µm | rod | 2.000 | 0.400 | FALSE | 0.400 |
| Tenacibaculum | 1.5-30 µm | 0.5 µm | rod | 1.500 | 0.500 | FALSE | 0.500 |
| Terasakiella | 2-3 µm | 0.5-0.6 µm | spiral | 2.000 | 0.500 | FALSE | 0.500 |
| Terasakiella | 1.5-4 µm | 0.3-0.5 µm | helical | 1.500 | 0.300 | FALSE | 0.300 |
| Teredinibacter | 3-6 µm | 0.4-0.6 µm | rod | 3.000 | 0.400 | FALSE | 0.400 |
| Teredinibacter | 3-6 µm | 0.4-0.6 µm | rod | 3.000 | 0.400 | FALSE | 0.400 |
| Teredinibacter | 3-6 µm | 0.4-0.6 µm | rod | 3.000 | 0.400 | FALSE | 0.400 |
| Thalassobaculum | 2.0-8.0 µm | 1.0-1.2 µm | rod | 2.000 | 1.000 | FALSE | 1.000 |
| Thalassobaculum | 1.4 µm | 0.4 µm | rod | 1.400 | 0.400 | FALSE | 0.400 |
| Thalassobius | 1.8-2.2 µm | 0.9-1.1 µm | rod | 1.800 | 0.900 | FALSE | 0.900 |
| Thalassolituus | NA | NA | coccus | NA | NA | FALSE | NA |
| Thalassospira | 1.7-2.8 µm | 0.5-0.8 µm | curved | 1.700 | 0.500 | FALSE | 0.500 |
| Thalassospira | 1.3 µm | 0.45 µm | rod | 1.300 | 0.450 | FALSE | 0.450 |
| Thalassospira | 1.45 µm | 0.25 µm | spiral | 1.450 | 0.250 | FALSE | 0.250 |
| Thalassospira | 1.55 µm | 0.55 µm | rod | 1.550 | 0.550 | FALSE | 0.550 |
| Thalassospira | 5.5 µm | 1.55 µm | rod | 5.500 | 1.550 | FALSE | 1.550 |
| Thalassotalea | 0.5-6.0 µm | 0.3-1.0 µm | pleomorphic | 0.500 | 0.300 | FALSE | 0.300 |
| Thalassotalea | 1.5-2.5 µm | 0.5-1 µm | rod | 1.500 | 0.500 | FALSE | 0.500 |
| Thalassotalea | 1-2 µm | 0.8-1 µm | rod | 1.000 | 0.800 | FALSE | 0.800 |
| Thalassotalea | 1-2.1 µm | 0.5-0.6 µm | rod | 1.000 | 0.500 | FALSE | 0.500 |
| Thalassotalea | 2.3-2.7 µm | 0.4-0.8 µm | rod | 2.300 | 0.400 | FALSE | 0.400 |
| Thalassotalea | 2.2-2.6 µm | 0.3-0.6 µm | rod | 2.200 | 0.300 | FALSE | 0.300 |
| Thalassotalea | 01-02 µm | 0.5-0.8 µm | rod | 1.000 | 0.500 | FALSE | 0.500 |
| Thalassotalea | 1.5-2.0 µm | 0.5-0.8 µm | rod | 1.500 | 0.500 | FALSE | 0.500 |
| Thalassotalea | 1.0-2.0 µm | 0.4-0.7 µm | rod | 1.000 | 0.400 | FALSE | 0.400 |
| Thermomarinilinea | NA | NA | NA | NA | NA | FALSE | NA |
| Thiogranum | NA | NA | NA | NA | NA | FALSE | NA |
| Thiohalobacter | 1.5 µm | 0.4 µm | rod | 1.500 | 0.400 | FALSE | 0.400 |
| Thioprofundum | NA | NA | NA | NA | NA | FALSE | NA |
| Thiothrix | 5.35 µm | 1.6 µm | rod | 5.350 | 1.600 | FALSE | 1.600 |
| Thiothrix | 4.85 µm | 1.55 µm | rod | 4.850 | 1.550 | FALSE | 1.550 |
| Tolumonas | 2.4-3.6 µm | 0.9 µm | rod | 2.400 | 0.900 | FALSE | 0.900 |
| Tolumonas | 2.5-3.2 µm | 0.9-1.2 µm | rod | 2.500 | 0.900 | FALSE | 0.900 |
| Tropicibacter | 0.8-1.4 µm | 0.8-1.3 µm | rod | 0.800 | 0.800 | FALSE | 0.800 |
| Tropicibacter | 2.25 µm | 0.4 µm | rod | 2.250 | 0.400 | FALSE | 0.400 |
| Truepera | NA | NA | NA | NA | NA | FALSE | NA |
| Vampirovibrio | NA | NA | NA | NA | NA | FALSE | NA |
| Vibrio | 0.9 µm | 0.5 µm | rod | 0.900 | 0.500 | FALSE | 0.500 |
| Vibrio | 1-1.5 µm | 0.5-0.7 µm | rod | 1.000 | 0.500 | FALSE | 0.500 |
| Vibrio | 1.5-1.8 µm | 0.6-1.2 µm | rod | 1.500 | 0.600 | FALSE | 0.600 |
| Vibrio | 3 µm | 1 µm | ovoid | 3.000 | 1.000 | FALSE | 1.000 |
| Vibrio | 1.5 µm | 0.75 µm | rod | 1.500 | 0.750 | FALSE | 0.750 |
| Vibrio | 1.5 µm | 0.75 µm | rod | 1.500 | 0.750 | FALSE | 0.750 |
| Vibrio | 1.6 µm | 0.75 µm | rod | 1.600 | 0.750 | FALSE | 0.750 |
| Vibrio | 01-02 µm | 0.9 µm | rod | 1.000 | 0.900 | FALSE | 0.900 |
| Vibrio | 2.5 µm | 0.45 µm | rod | 2.500 | 0.450 | FALSE | 0.450 |
| Vibrio | 2.5 µm | 1 µm | rod | 2.500 | 1.000 | FALSE | 1.000 |
| Vibrio | 2 µm | 1 µm | rod | 2.000 | 1.000 | FALSE | 1.000 |
| Vibrio | 2.1 µm | 0.55 µm | rod | 2.100 | 0.550 | FALSE | 0.550 |
| Vibrionimonas | NA | NA | NA | NA | NA | TRUE | NA |
| Wandonia | NA | NA | NA | NA | NA | TRUE | NA |
| Wenxinia | 1.3 µm | 0.75 µm | ovoid | 1.300 | 0.750 | FALSE | 0.750 |
| Winogradskyella | 1.6-1.7 µm | 0.5-0.6 µm | rod | 1.600 | 0.500 | FALSE | 0.500 |
| Winogradskyella | 1-9 µm | 0.2-0.5 µm | rod | 1.000 | 0.200 | FALSE | 0.200 |
| Winogradskyella | 1.8-2.2 µm | 0.7-1.1 µm | rod | 1.800 | 0.700 | FALSE | 0.700 |
| Winogradskyella | 1.5-2.1 µm | 0.6-0.8 µm | rod | 1.500 | 0.600 | FALSE | 0.600 |
| Winogradskyella | 1.5-2 µm | 0.4-0.5 µm | rod | 1.500 | 0.400 | FALSE | 0.400 |
| Winogradskyella | 0.8-1.4 µm | 0.4-0.6 µm | rod | 0.800 | 0.400 | FALSE | 0.400 |
| Winogradskyella | 3 µm | 0.7-0.8 µm | rod | 3.000 | 0.700 | FALSE | 0.700 |
| Winogradskyella | 0.75 µm | 0.5 µm | rod | 0.750 | 0.500 | FALSE | 0.500 |
| Winogradskyella | 3.45 µm | 0.45 µm | rod | 3.450 | 0.450 | FALSE | 0.450 |
| Winogradskyella | 1 µm | 0.4 µm | rod | 1.000 | 0.400 | FALSE | 0.400 |
| Winogradskyella | 0.8-1.8 µm | 0.4-0.9 µm | rod | 0.800 | 0.400 | FALSE | 0.400 |
| Winogradskyella | 0.8-1.8 µm | 0.3-0.4 µm | rod | 0.800 | 0.300 | FALSE | 0.300 |
| Winogradskyella | 2.1 µm | 0.25 µm | rod | 2.100 | 0.250 | FALSE | 0.250 |
| Woodsholea | 3.5 µm | 0.7 µm | rod | 3.500 | 0.700 | FALSE | 0.700 |
| Xanthomonas | NA | NA | rod | NA | NA | FALSE | NA |
| Zeaxanthinibacter | 2.5-6.0 µm | 0.3-0.7 µm | rod | 2.500 | 0.300 | FALSE | 0.300 |
| Zeaxanthinibacter | 2.55 µm | 0.3 µm | rod | 2.550 | 0.300 | FALSE | 0.300 |

# Appendix S6: Sample metadata

| Sample | Location | GPS | East or West of Wallace Line |
| --- | --- | --- | --- |
| Komodo\_01 | Komodo | -8.547633 119.813276 | East |
| Komodo\_02 | Komodo | -8.547633 119.813276 | East |
| Komodo\_03 | Komodo | -8.547633 119.813276 | East |
| Komodo\_04 | Komodo | -8.547633 119.813277 | East |
| Komodo\_05 | Komodo | -8.547633 119.813278 | East |
| Komodo\_06 | Komodo | -8.547633 119.813279 | East |
| Komodo\_07 | Komodo | -8.547633 119.813280 | East |
| Komodo\_08 | Komodo | -8.547633 119.813281 | East |
| Komodo\_09 | Komodo | -8.547633 119.813282 | East |
| Komodo\_10 | Komodo | -8.547633 119.813283 | East |
| Komodo\_11 | Komodo | -8.547633 119.813284 | East |
| Komodo\_12 | Komodo | -8.547633 119.813285 | East |
| Komodo\_13 | Komodo | -8.547633 119.813286 | East |
| Komodo\_14 | Komodo | -8.547633 119.813287 | East |
| Komodo\_15 | Komodo | -8.547633 119.813288 | East |
| Komodo\_16 | Komodo | -8.547633 119.813289 | East |
| Alor\_01 | Alor | -8.250097 124.458291 | East |
| Alor\_02 | Alor | -8.250097 124.458291 | East |
| Alor\_03 | Alor | -8.250097 124.458291 | East |
| Alor\_04 | Alor | -8.250097 124.458291 | East |
| Alor\_05 | Alor | -8.250097 124.458291 | East |
| Alor\_06 | Alor | -8.250097 124.458291 | East |
| Alor\_07 | Alor | -8.250097 124.458291 | East |
| Alor\_08 | Alor | -8.250097 124.458291 | East |
| Alor\_09 | Alor | -8.250097 124.458291 | East |
| Alor\_10 | Alor | -8.250097 124.458291 | East |
| Alor\_11 | Alor | -8.250097 124.458291 | East |
| Alor\_12 | Alor | -8.250097 124.458291 | East |
| Alor\_13 | Alor | -8.250097 124.458291 | East |
| Alor\_14 | Alor | -8.250097 124.458291 | East |
| Alor\_15 | Alor | -8.250097 124.458291 | East |
| Alor\_16 | Alor | -8.250097 124.458291 | East |
| Wakatobi\_01 | Wakatobi | -5.756483 124.160527 | East |
| Wakatobi\_02 | Wakatobi | -5.756483 124.160527 | East |
| Wakatobi\_03 | Wakatobi | -5.756483 124.160527 | East |
| Wakatobi\_04 | Wakatobi | -5.756483 124.160527 | East |
| Wakatobi\_05 | Wakatobi | -5.756483 124.160527 | East |
| Wakatobi\_06 | Wakatobi | -5.756483 124.160527 | East |
| Wakatobi\_07 | Wakatobi | -5.756483 124.160527 | East |
| Wakatobi\_08 | Wakatobi | -5.756483 124.160527 | East |
| Wakatobi\_09 | Wakatobi | -5.756483 124.160527 | East |
| Wakatobi\_10 | Wakatobi | -5.756483 124.160527 | East |
| Wakatobi\_11 | Wakatobi | -5.756483 124.160527 | East |
| Wakatobi\_12 | Wakatobi | -5.756483 124.160527 | East |
| Wakatobi\_13 | Wakatobi | -5.756483 124.160527 | East |
| Wakatobi\_14 | Wakatobi | -5.756483 124.160527 | East |
| Wakatobi\_15 | Wakatobi | -5.756483 124.160527 | East |
| Wakatobi\_16 | Wakatobi | -5.756483 124.160527 | East |
| Karimunjawa\_01 | Karimunjawa | -5.860486 110.408675 | West |
| Karimunjawa\_02 | Karimunjawa | -5.860486 110.408675 | West |
| Karimunjawa\_03 | Karimunjawa | -5.860486 110.408675 | West |
| Karimunjawa\_04 | Karimunjawa | -5.860486 110.408675 | West |
| Karimunjawa\_05 | Karimunjawa | -5.860486 110.408675 | West |
| Karimunjawa\_06 | Karimunjawa | -5.860486 110.408675 | West |
| Karimunjawa\_07 | Karimunjawa | -5.860486 110.408675 | West |
| Karimunjawa\_08 | Karimunjawa | -5.860486 110.408675 | West |
| Karimunjawa\_09 | Karimunjawa | -5.860486 110.408675 | West |
| Karimunjawa\_10 | Karimunjawa | -5.860486 110.408675 | West |
| Karimunjawa\_11 | Karimunjawa | -5.860486 110.408675 | West |
| Karimunjawa\_12 | Karimunjawa | -5.860486 110.408675 | West |
| Karimunjawa\_13 | Karimunjawa | -5.860486 110.408675 | West |
| Karimunjawa\_14 | Karimunjawa | -5.860486 110.408675 | West |
| Karimunjawa\_15 | Karimunjawa | -5.860486 110.408675 | West |
| Karimunjawa\_16 | Karimunjawa | -5.860486 110.408675 | West |
| Tual\_01 | Tual | -5.765438 132.669491 | East |
| Tual\_02 | Tual | -5.765438 132.669491 | East |
| Tual\_03 | Tual | -5.765438 132.669491 | East |
| Tual\_04 | Tual | -5.765438 132.669491 | East |
| Tual\_05 | Tual | -5.765438 132.669491 | East |
| Tual\_06 | Tual | -5.765438 132.669491 | East |
| Tual\_07 | Tual | -5.765438 132.669491 | East |
| Tual\_08 | Tual | -5.765438 132.669491 | East |
| Tual\_09 | Tual | -5.765438 132.669491 | East |
| Tual\_10 | Tual | -5.765438 132.669491 | East |
| Tual\_11 | Tual | -5.765438 132.669491 | East |
| Tual\_12 | Tual | -5.765438 132.669491 | East |
| Tual\_13 | Tual | -5.765438 132.669491 | East |
| Tual\_14 | Tual | -5.765438 132.669491 | East |
| Tual\_15 | Tual | -5.765438 132.669491 | East |
| Tual\_16 | Tual | -5.765438 132.669491 | East |
| Halmahera\_01 | Halmahera | 1.753725 128.023338 | East |
| Halmahera\_02 | Halmahera | 1.753725 128.023338 | East |
| Halmahera\_03 | Halmahera | 1.753725 128.023338 | East |
| Halmahera\_04 | Halmahera | 1.753725 128.023338 | East |
| Halmahera\_05 | Halmahera | 1.753725 128.023338 | East |
| Halmahera\_06 | Halmahera | 1.753725 128.023338 | East |
| Halmahera\_07 | Halmahera | 1.753725 128.023338 | East |
| Halmahera\_08 | Halmahera | 1.753725 128.023338 | East |
| Halmahera\_09 | Halmahera | 1.753725 128.023338 | East |
| Halmahera\_10 | Halmahera | 1.753725 128.023338 | East |
| Halmahera\_11 | Halmahera | 1.753725 128.023338 | East |
| Halmahera\_12 | Halmahera | 1.753725 128.023338 | East |
| Halmahera\_13 | Halmahera | 1.753725 128.023338 | East |
| Halmahera\_14 | Halmahera | 1.753725 128.023338 | East |
| Halmahera\_15 | Halmahera | 1.753725 128.023338 | East |
| Halmahera\_16 | Halmahera | 1.753725 128.023338 | East |
| Belitung\_01 | Belitung | -2.952949 108.271793 | West |
| Belitung\_02 | Belitung | -2.952949 108.271793 | West |
| Belitung\_03 | Belitung | -2.952949 108.271793 | West |
| Belitung\_04 | Belitung | -2.952949 108.271793 | West |
| Belitung\_05 | Belitung | -2.952949 108.271793 | West |
| Belitung\_06 | Belitung | -2.952949 108.271793 | West |
| Belitung\_07 | Belitung | -2.952949 108.271793 | West |
| Belitung\_08 | Belitung | -2.952949 108.271793 | West |
| Belitung\_09 | Belitung | -2.952949 108.271793 | West |
| Belitung\_10 | Belitung | -2.952949 108.271793 | West |
| Belitung\_11 | Belitung | -2.952949 108.271793 | West |
| Belitung\_12 | Belitung | -2.952949 108.271793 | West |
| Belitung\_13 | Belitung | -2.952949 108.271793 | West |
| Belitung\_14 | Belitung | -2.952949 108.271793 | West |
| Belitung\_15 | Belitung | -2.952949 108.271793 | West |
| Belitung\_16 | Belitung | -2.952949 108.271793 | West |
| Bangka\_01 | Bangka | 1.743565 125.161338 | West |
| Bangka\_02 | Bangka | 1.743565 125.161338 | East |
| Bangka\_03 | Bangka | 1.743565 125.161338 | East |
| Bangka\_04 | Bangka | 1.743565 125.161338 | East |
| Bangka\_05 | Bangka | 1.743565 125.161338 | East |
| Bangka\_06 | Bangka | 1.743565 125.161338 | East |
| Bangka\_07 | Bangka | 1.743565 125.161338 | East |
| Bangka\_08 | Bangka | 1.743565 125.161338 | East |
| Bangka\_09 | Bangka | 1.743565 125.161338 | East |
| Bangka\_10 | Bangka | 1.743565 125.161338 | East |
| Bangka\_11 | Bangka | 1.743565 125.161338 | East |
| Bangka\_12 | Bangka | 1.743565 125.161338 | East |
| Bangka\_13 | Bangka | 1.743565 125.161338 | East |
| Bangka\_14 | Bangka | 1.743565 125.161338 | East |
| Bangka\_15 | Bangka | 1.743565 125.161338 | East |
| Bangka\_16 | Bangka | 1.743565 125.161338 | East |
| Bali\_01 | Bali | -8.160796 115.023966 | West |
| Bali\_02 | Bali | -8.160796 115.023966 | West |
| Bali\_03 | Bali | -8.160796 115.023966 | West |
| Bali\_04 | Bali | -8.160796 115.023966 | West |
| Bali\_05 | Bali | -8.160796 115.023966 | West |
| Bali\_06 | Bali | -8.160796 115.023966 | West |
| Bali\_07 | Bali | -8.160796 115.023966 | West |
| Bali\_08 | Bali | -8.160796 115.023966 | West |
| Bali\_09 | Bali | -8.160796 115.023966 | West |
| Bali\_10 | Bali | -8.160796 115.023966 | West |
| Bali\_11 | Bali | -8.160796 115.023966 | West |
| Bali\_12 | Bali | -8.160796 115.023966 | West |
| Bali\_13 | Bali | -8.160796 115.023966 | West |
| Bali\_14 | Bali | -8.160796 115.023966 | West |
| Bali\_15 | Bali | -8.160796 115.023966 | West |
| Bali\_16 | Bali | -8.160796 115.023966 | West |
| Derawan\_01 | Derawan | 2.289539 118.256755 | West |
| Derawan\_02 | Derawan | 2.289539 118.256755 | West |
| Derawan\_03 | Derawan | 2.289539 118.256755 | West |
| Derawan\_04 | Derawan | 2.289539 118.256755 | West |
| Derawan\_05 | Derawan | 2.289539 118.256755 | West |
| Derawan\_06 | Derawan | 2.289539 118.256755 | West |
| Derawan\_07 | Derawan | 2.289539 118.256755 | West |
| Derawan\_08 | Derawan | 2.289539 118.256755 | West |
| Derawan\_09 | Derawan | 2.289539 118.256755 | West |
| Derawan\_10 | Derawan | 2.289539 118.256755 | West |
| Derawan\_11 | Derawan | 2.289539 118.256755 | West |
| Derawan\_12 | Derawan | 2.289539 118.256755 | West |
| Derawan\_13 | Derawan | 2.289539 118.256755 | West |
| Derawan\_14 | Derawan | 2.289539 118.256755 | West |
| Derawan\_15 | Derawan | 2.289539 118.256755 | West |
| Derawan\_16 | Derawan | 2.289539 118.256755 | West |
| Banggai\_01 | Banggai | -1.923214 123.145655 | East |
| Banggai\_02 | Banggai | -1.923214 123.145655 | East |
| Banggai\_03 | Banggai | -1.923214 123.145655 | East |
| Banggai\_04 | Banggai | -1.923214 123.145655 | East |
| Banggai\_05 | Banggai | -1.923214 123.145655 | East |
| Banggai\_06 | Banggai | -1.923214 123.145655 | East |
| Banggai\_07 | Banggai | -1.923214 123.145655 | East |
| Banggai\_08 | Banggai | -1.923214 123.145655 | East |
| Banggai\_09 | Banggai | -1.923214 123.145655 | East |
| Banggai\_10 | Banggai | -1.923214 123.145655 | East |
| Banggai\_11 | Banggai | -1.923214 123.145655 | East |
| Banggai\_12 | Banggai | -1.923214 123.145655 | East |
| Banggai\_13 | Banggai | -1.923214 123.145655 | East |
| Banggai\_14 | Banggai | -1.923214 123.145655 | East |
| Banggai\_15 | Banggai | -1.923214 123.145655 | East |
| Banggai\_16 | Banggai | -1.923214 123.145655 | East |
| Pari\_01 | Pari | -5.863506 106.608923 | West |
| Pari\_02 | Pari | -5.863506 106.608924 | West |
| Pari\_03 | Pari | -5.863506 106.608925 | West |
| Pari\_04 | Pari | -5.863506 106.608926 | West |
| Pari\_05 | Pari | -5.863506 106.608927 | West |
| Pari\_06 | Pari | -5.863506 106.608928 | West |
| Pari\_07 | Pari | -5.863506 106.608929 | West |
| Pari\_08 | Pari | -5.863506 106.608930 | West |
| Pari\_09 | Pari | -5.863506 106.608931 | West |
| Pari\_10 | Pari | -5.863506 106.608932 | West |
| Pari\_11 | Pari | -5.863506 106.608933 | West |
| Pari\_12 | Pari | -5.863506 106.608934 | West |
| Pari\_13 | Pari | -5.863506 106.608935 | West |
| Pari\_14 | Pari | -5.863506 106.608936 | West |
| Pari\_15 | Pari | -5.863506 106.608937 | West |
| Pari\_16 | Pari | -5.863506 106.608938 | West |
| Blank\_01 | N/A | N/A | N/A |
| Blank\_02 | N/A | N/A | N/A |
| Blank\_03 | N/A | N/A | N/A |
| Blank\_04 | N/A | N/A | N/A |
| Blank\_05 | N/A | N/A | N/A |
| Blank\_06 | N/A | N/A | N/A |
| Blank\_07 | N/A | N/A | N/A |
| Blank\_08 | N/A | N/A | N/A |
